# Long-term effects of functional appliances in treated versus untreated patients with Class II malocclusion: A systematic review and meta-analysis

**DOI:** 10.1371/journal.pone.0221624

**Published:** 2019-09-06

**Authors:** Giorgio Cacciatore, Alessandro Ugolini, Chiarella Sforza, Oghenekome Gbinigie, Annette Plüddemann

**Affiliations:** 1 Department for Continuing Education, University of Oxford, Oxford, England, United Kingdom; 2 Department of Orthodontics, Università degli Studi di Genova, Genova, Italy; 3 Department of Biomedical Sciences for Health, Università degli Studi di Milano, Milan, Italy; 4 Nuffield Department of Primary Care Health Sciences, University of Oxford, Oxford, England, United Kingdom; Harvard School of Dental Medicine, UNITED STATES

## Abstract

**Objective:**

To assess the cephalometric skeletal and soft-tissue of functional appliances in treated versus untreated Class II subjects in the long-term (primarily at the end of growth, secondarily at least 3 years after retention).

**Search methods:**

Unrestricted electronic search of 24 databases and additional manual searches up to March 2018.

**Selection criteria:**

Randomised and non-randomised controlled trials reporting on cephalometric skeletal and soft-tissue measurements of Class II patients (aged 16 years or under) treated with functional appliances, worn alone or in combination with multi-bracket therapy, compared to untreated Class II subjects.

**Data collection and analysis:**

Mean differences (MDs) and 95% confidence intervals (95% CIs) were calculated with the random-effects model. Data were analysed at 2 primary time points (above 18 years of age, at the end of growth according to the Cervical Vertebral Maturation method) and a secondary time point (at least 3 years after retention). The risk of bias and quality of evidence were assessed according to the ROBINS tool and GRADE system, respectively.

**Results:**

Eight non-randomised studies published in 12 papers were included. Functional appliances produced a significant improvement of the maxillo-mandibular relationship, at almost all time points (Wits appraisal at the end of growth, MD -3.52 mm, 95% CI -5.11 to -1.93, P < 0.0001). The greatest increase in mandibular length was recorded in patients aged 18 years and above (Co-Gn, MD 3.20 mm, 95% CI 1.32 to 5.08, P = 0.0009), although the improvement of the mandibular projection was negligible or not significant. The quality of evidence was ‘very low’ for most of the outcomes at both primary time points.

**Conclusions:**

Functional appliances may be effective in correcting skeletal Class II malocclusion in the long-term, however the quality of the evidence was very low and the clinical significance was limited.

**Systematic review registration:**

CRD42018092139

## Introduction

### Rationale

Class II malocclusion is the most prevalent antero-posterior jaw problem in orthodontics, affecting one third of the population [1, 2]. The majority of Class II patients exhibit mandibular skeletal retrusion [3, 4]. Reduced mandibular size is also a major feature of Class II malocclusion patients [5]. As a result, there has been great interest in the use of ‘functional appliances’, designed primarily to influence the lower dentition and enhance the growth of the mandible [3]. These appliances promote forward posturing of the mandible, although their effects also impact on the upper jaw [6, 7].

The potential that functional appliances could modify skeletal growth is of great importance for patients and orthodontists alike. Improving facial aesthetics is one of the main reasons for seeking orthodontic treatment [8] and it is associated with a high level of patient and parent satisfaction [9]. Mandibular retrusion has a negative impact on perceived attractiveness [10], self-esteem and oral health-related quality of life [11]. The magnitude of the retrusion is also an important factor in treatment decision-making. Small skeletal discrepancies may only need multi-bracket therapy for the correction of malocclusion and refinement of teeth alignment. On the other hand, greater discrepancies may require a surgical treatment to modify the position and length of skeletal structures and to attain better aesthetic results [12].

Post-pubertal growth has been shown to produce dramatic alterations in skeletal and dental relationships [13]. There is no consensus on the age at which growth ends [14–18]. Overall, growth continues up to mid-adulthood, with different patterns in the two genders. Males show an anterior rotation of the mandible, whereas females demonstrate a posterior mandibular rotation [17, 18]. An alternative method to establish when growth comes to an end is through using indicators of the growth phase, such as the hand-and-wrist maturation method [19] or the cervical vertebral maturation method [20].

To fully understand the real effects of functional appliances on the growth of the jaws and profile, it is essential to study these effects at the completion of patient growth, when biases and confounding factors due to natural changes are negligible. The long-term stability of these changes is important too.

To date, most systematic reviews investigating the treatment effects of functional appliances in Class II malocclusion patients have synthesized studies evaluating the skeletal and soft-tissue changes at the end of the orthodontic treatment [6, 7, 21–26]. Only two reviews systematically searched for scientific evidence concerning the long-term stability of treatment results achieved by Class II functional appliance therapy [27, 28]. Another systematic review is ongoing [29]. No previous reviews determined the effects of removable and fixed functional appliances in patients with Class II malocclusion compared to untreated controls at growth completion.

### Objective

The objective of this systematic review was therefore to assess the skeletal and soft-tissue effects measured on lateral cephalograms produced by functional appliances in treated versus untreated Class II subjects in the long-term (primarily at the end of growth, secondarily at least 3 years after retention).

## Materials and methods

### Protocol and registration

The present systematic review was performed according to the guidelines of the Cochrane Handbook for Systematic Reviews of Interventions Version 5.1.0 [30], and is reported on the basis of the Preferred Reporting Items for Systematic Reviews and Meta-Analyses (PRISMA) statement (S1 table [31]). The protocol was published in the International Prospective Register of Systematic Reviews (PROSPERO) on 03 April 2018 (registration number CRD42018092139).

### Information sources

The search strategy covered 11 bibliographic databases, 10 non-bibliographic databases and 3 unpublished studies sources, from their launch to March 2018 [32–35]. Hand-searching of the most common orthodontic journals was performed as well. The Cochrane Master List was consulted to facilitate the identification of these journals [30, 34, 36]. The reference lists of the trials eligible for inclusion and systematic reviews concerning Class II malocclusion treatment were also checked. Information concerning the name of the search source, the date range that were searched, and, for electronic databases, the search platform or provider are presented in S2 table.

### Search

Search strategies were developed using medical subject headings (MeSH) and text words related to functional appliances. The search strategies of the preliminarily identified systematic reviews published between 2015 and 2018 were collected [6, 7, 21–26, 28]. As recommended by the Cochrane Collaboration [30], terms related to only three aspects of the review’s question were selected: participants, interventions and timing.

Preliminary searches were conducted to screen the list of queries and define the MEDLINE and Google Scholar search strategies. After the MEDLINE strategy had been finalised, it was adapted to the syntax and subjects headings of the other databases. No restrictions based on language, publication year, or publication status were applied to the search. The search strategy designed for each database is shown in S3 table.

### Eligibility criteria

Randomised and non-randomised controlled trials reporting on cephalometric skeletal and soft-tissue measurements of Class II patients (aged 16 years or under) treated with functional appliances, worn alone or in combination with multi-bracket therapy, compared to untreated Class II subjects were included (Table 1). The rationale behind eligibility criteria is provided in S1 Appendix.

**Table 1:** Eligibility criteria used for the study selection.

Category	Inclusion	Exclusion
Study designs	Randomised controlled trials (RCTs), controlled (non-randomised) clinical trials (CCTs), controlled before-after (CBA) studies, and case-control or nested case-control studies	Prospective and retrospective cohort studies, cross-sectional studies, case series, and case reports
Participants	Children and adolescents (aged 16 years or under) receiving orthodontic treatment to correct Class II malocclusion	Participants with a cleft lip or palate or both, other craniofacial deformity/syndrome (such as Apert, Crouzon, Hemifacial Microsomia/Goldenhar, Moebius, Pierre Robin, Treacher Collins syndromes or craniosynostosis), syndromes affecting the craniofacial structures or patients with temporo-mandibular joint disorders
	Active treatment with functional appliances had to be completed by the age of 16 years	
Interventions	Any type of functional appliance, defined as a removable or fixed orthodontic appliance that postures the mandible forward	Association with other Class II devices designed primarily to restrain the maxilla (e.g. headgear)
	Functional appliances worn alone or in combination with multi-bracket therapy. When functional appliances were worn alone, this therapy could also take place after the functional appliance treatment.	
	Functional appliances worn for 6 months or longer	
Comparators	Untreated Class II subjects	
	Groups with similar ages at the commencement of the observational period (age differences between the treated and untreated groups less than 18 months)	
Outcomes	Cephalometric skeletal measurements evaluating the antero-posterior position of the maxilla and mandible, the total mandibular length or length of its parts (ramus and corpus), the mutual relationship between the two jaws	
	Soft tissue changes of both lips and chin, measured on lateral cephalograms	
Timing	At the end of growth, defined by age or using indicators of the growth phase	
	Post-retention period of at least 3 years	

### Study selection

Search results from those databases allowing for the export of valid file formats (MEDLINE, EMBASE, CENTRAL, LILACS, Web of Science, Scopus and ProQuest Dissertation & Theses) were uploaded to EndNote software. Results from Google Scholar, TRIP Database, British Library Direct, ISI proceedings, hand-searching, unpublished and ongoing studies were managed manually. A calibration exercise was undertaken to pilot and refine the screening questions, before initiating the formal screening process.

G.C. and A.U. independently screened the titles and abstracts to remove obviously irrelevant reports. After having retrieved full texts of potentially relevant and unclear reports, the reviewers examined if these met the eligibility criteria. Multiple reports of the same study were linked together at the end of the selection process [30]. G.C. sought additional information from study authors when it was deemed necessary to resolve questions about eligibility. Reviewers resolved disagreements by discussion, and an arbitrator (C.S.) adjudicated unresolved disagreements. Primary reasons for excluding trials were recorded.

### Data collection process

G.C. and A.U. independently extracted data using a piloted data extraction form. This electronic form originated from those proposed by the Cochrane Collaboration [30] and a previous Cochrane review on Class II malocclusion [26]. To ensure consistency across reviewers, calibration exercises were conducted before starting the review. Disagreements were resolved through discussion.

### Data items

Information was extracted from each included study on source and general information, methods, characteristics of participants and interventions, outcomes, data and analysis.

### Risk of bias in individual studies

The risk of bias tool for non-randomised studies of interventions (ROBINS-I tool [37]) was used to ascertain the quality of the evidence of included trials.

### Summary measures

Data were summarised and considered suitable for pooling only if the same cephalometric measurement was used for the same outcome. To circumvent the issue of the different follow-up periods of included studies, the overall treatment and post-treatment changes were analysed [30]. Mean differences (MDs) and 95% confidence intervals (95% CIs) between these changes were calculated. Whenever necessary, the enlargement of linear measurements due to the radiographic examination was adjusted at 0%. Studies in which the magnification was not reported for linear measurements were excluded from meta-analyses.

Skewed data and non-quantitative data were presented in narrative format.

### Synthesis of results

The random-effects model proposed by DerSimonian and Laird [38] was chosen a priori to combine and compare data from included studies. The presence of statistical heterogeneity was assessed by inspecting the overlap of the confidence intervals in the forest plots and by using the chi-squared (Chi^2^) test, while the impact of between-study heterogeneity on the meta-analysis was tested by calculating the τ^2^ and the I^2^ statistics [39].

Since variation applies as much within studies as across them, the choice to treat each independent subgroup as a separate study was preferred to computing a composite effect for each study and using it in the analysis [40].

As there is no consensus on the age at which growth ends, treatment effects were evaluated at 2 primary time points:

■ Above 18 years of age. The age threshold of 18 years was chosen to maximise the data available [30];
■ At the end of growth documented by the Cervical Vertebral Maturation (CVM) method (cervical vertebral maturation stage 5 or 6 [20]);

A secondary time point was established after a post-retention period of at least 3 years.

### Additional analysis

Subgroup and sensitivity analyses were performed in order to explore the source of heterogeneity and test the overall robustness of the data, respectively. All subgroup and sensitivity analyses were pre-specified in the protocol.

For all outcomes, results were divided according to the type of functional appliance.

For the most clinically important outcomes, subgroup analyses were based on the following:

■ Patient characteristics (gender);
■ Beginning of the functional appliance therapy according to age (early treatments, commencing in children aged between 7 and 11 years; late treatments, beginning in adolescents aged between 12 and 16 years);
■ Start of the treatment according to the cervical vertebral maturation method (early treatments, with patients presenting with Cervical Vertebral Maturation Stage [CVMS] 1 or 2 at the first observation; late treatments, with subjects presenting with CVMS 2 or 3);
■ Post-retention period duration (3–4, 5–10 years after active treatment with functional appliances);

Sensitivity analysis was performed to examine the impact of the study quality assessment on the overall estimates of effect.

### Risk of bias across studies

Outcome reporting bias and publication bias were evaluated. In order to determine whether reporting bias was present, the Clinical Trial Register was screened using the International Clinical Trials Registry Platform of the World Health Organisation (http://apps.who.int/trialssearch). When protocols were identified, discrepancies between the outcomes planned in the protocol and those reported in the final manuscript were assessed. The potential for reporting bias was explored by funnel plots if ≥10 studies were available [40].

The quality of evidence for all outcomes at both primary time points was judged using the Grading of Recommendations Assessment, Development and Evaluation working group methodology [41].

## Results

### Study selection

**Fig 1:**
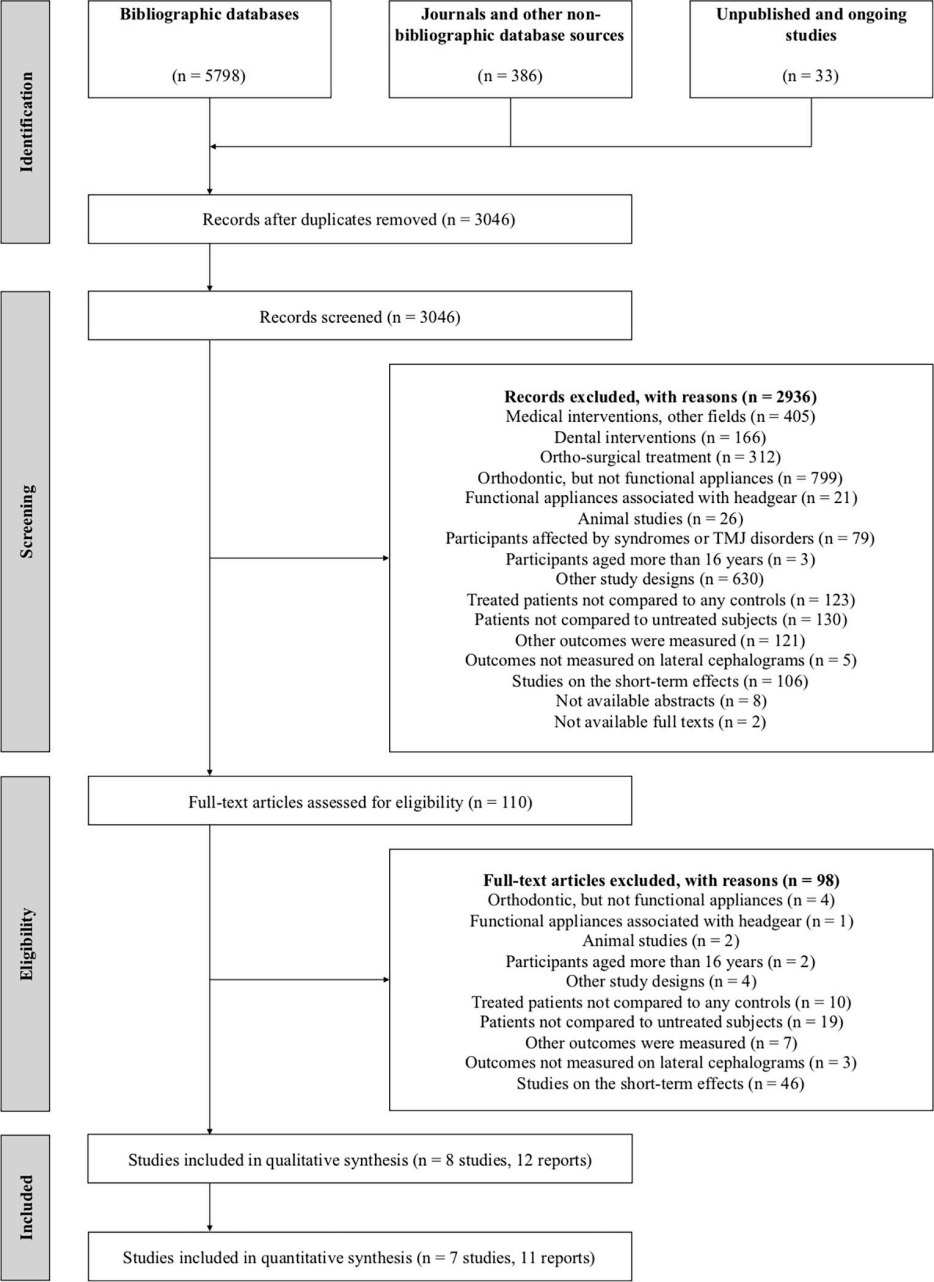
PRISMA flow diagram.

The results of the search are summarised in Fig 1. Among 3046 records, eight non-randomised studies published in 12 papers were identified for inclusion in this review [42–49]. Two authors were contacted to clarify whether duplicate data was used in their trials. Since the study by Pavoni et al. [43] contained partial data of previous studies [50–52] and has the greater sample size and subgroup analysis, it was considered the reference study of the other reports. The thesis by Wigal [47] with complete data of the subsequent published study [53] was included as well. Excluded studies with reasons are listed in supplementary files (S4 Table, S2 Appendix).

### Study characteristics

The main characteristics of the 8 included studies are presented in Tables 2–3. All the studies were retrospective controlled clinical trials [42–49]. A wide range of eligibility criteria was found in the included studies. Class II malocclusion was defined by both skeletal and dental parameters. Six trials used historical controls for the comparison with treated patients [43, 45, 46–49].

**Table 2:** Characteristics of included studies (participants, interventions, outcomes).

Study	Groups (N)	Participants	Interventions		Outcomes		
			T1-T2	T2-T3	Mx skeletal	Md skeletal	Mx-Md skeletal
Wieslander 1979	TG (23)	ANB > 6 degrees, full Class II molar relationship, mixed dentition	Act	None	A to S perp	Pg to S perp, Co-Gn, Ar-Gn, Co to mand	ANB
	CG (23)	Matched according to gender, age, ethnicity, and socioeconomic background	None	None			
Pavoni 2017	TG (46)	ANB > 4 degrees, full Class II or end-to-end molar relationship, excessive overjet (greater than 5 mm)	Bio / Act	MBA	SNA	SNB, Pg to N perp, Co-Gn, Co-Go	ANB, Wits
	CG (31)	Matched according to age and skeletal maturation, and starting cephalometric characteristics	None	None			
Falck 1991	TG (50)	Class II division 1 malocclusion (no definition)	Fr2	-	Horiz. A to ORS	Horiz. B or Pg to ORS, Co-Gn	-
	CG (38)	Matched according to gender and age	None	None			
Freeman 2009	TG (30)	Full Class II molar relationship, excessive overjet (no definition)	Fr2	-	SNA, A to N perp, Co-A	SNB, Pg to N perp, Co-Gn	ANB, Wits, Co-Gn/Co-A diff
	CG (20)	Matched according to gender, age and skeletal maturation, and starting cephalometric characteristics	None	None			
Angelieri 2014	TG (17)	ANB > 2 degrees, full Class II or end-to-end molar relationship, excessive overjet (greater than 5 mm), late mixed dentition	Fr2	Fr2 / None	SNA, A to N perp, Co-A	SNB, Pg to N perp, Co-Gn	ANB, Wits, Co-Gn/Co-A diff
	CG (17)	Matched according to gender, age and skeletal maturation	None	None			
Wigal 2008	TG (22)	ANB > 4 degrees, mixed dentition	Hb	MBA	SNA, Co-A, Olp-A	SNB, Co-Gn, Olp-Pg, Olp-Co	ANB, Wits, Co-Gn/Co-A diff
	CG (22)	Matched according to gender, age, and starting cephalometric characteristics	None	None			
Drosen 2018	TG (13)	Class II malocclusion (no definition)	Hb +/- MBA	Act / None	SNA	SNB, Ar-Go	ANB, Wits
	CG (13)	Matched according to gender and age	None	None			
Alhoraibi 2017	TG (39)	ANB > 4 degrees, full Class II or end-to-end molar relationship, excessive overjet (greater than 10 mm)	FRD + MBA	None	SNA, A to N perp, Co-A	SNB, Pg to N perp, Co-Gn	ANB, Wits, Co-Gn/Co-A diff
	CG (39)	Matched according to gender, age and skeletal maturation, and starting cephalometric characteristics	None	None			

N, number of participants; TG, treated group; CG, control group

Act, Activator; Bio, Bionator; Fr2, Frankel-2; Hb, Herbst; FRD, Forsus; MBA, multi-bracket appliances

Mx skeletal, maxillary skeletal outcomes; SNA, SNA angle; A to N perp, A point to N perpendicular distance; A to S perp, A point to S perpendicular distance; Horiz. A to ORS, horizontal distance of A point to occipital reference system; Co-A, Co-A distance; Olp-A, distance of A point to occlusal line perpendicular

Md skeletal, mandibular skeletal outcomes; SNB, SNB angle; Pg to N perp, Pg point to N perpendicular distance; Pg to S perp, Pg point to S perpendicular distance; Horiz. B or Pg to ORS, horizontal distance of B point or Pg point to occipital reference system; Co-Gn, Co-Gn distance; Ar-Gn, Ar-Gn distance; Olp-Pg, distance of Pg point to occlusal line perpendicular; Olp-Co, distance of Co point to occlusal line perpendicular; Co to mand, distance of Co point to mandibular plane; Co-Go, Co-Go distance; Ar-Go, Ar-Go distance

Mx-md skeletal, maxillo-mandibular outcomes; ANB, ANB angle; Wits, Wits appraisal; Co-Gn/Co-A diff, Co-Gn/Co-A difference.

**Table 3:** Characteristics of included studies (timing).

Study or subgroup	Groups (N)	Timing											
		T1			T2			T3			T1-T2	T2-T3	T1-T3
		Mean	SD	CVSM	Mean	SD	CVSM	Mean	SD	CVSM			
Wieslander 1979	TG (23)	~ 10	-	-	~ 13	-	-	~ 17	-	-	3.0	4.0	7.0
	CG (23)	~ 10	-	-	~ 13	-	-	~ 17	-	-	3.0	4.0	7.0
Pavoni 2017 (early)	TG (23)	9.5	1.2	1-2	11.4	1.2	1-3	17.9	2.3	5-6	1.9	6.5	8.4
	CG (16)	9.4	0.7	1-2	11.3	0.7	1-3	17.0	1.8	5-6	1.9	5.7	7.6
Pavoni 2017 (late)	TG (23)	10.2	1.3	2-3	12.5	1.2	4-5	18.5	2.1	5-6	2.3	6.0	8.3
	CG (15)	10.8	1.1	2-3	12.7	1.2	4-5	18.3	1.3	5-6	1.9	5.6	7.5
Falck 1991 (males)	TG (19)	7.3	-	-	-	-	-	17.5	-	-	-	-	10.2
	CG (18)	7.0	-	-	-	-	-	16.4	-	-	-	-	9.4
Falck 1991 (females)	TG (31)	7.3	-	-	-	-	-	17.2	-	-	-	-	9.9
	CG (20)	7.7	-	-	-	-	-	17.9	-	-	-	-	10.2
Freeman 2009	TG (30)	8.1	1.3	1-2	-	-	-	18.0	3.4	5-6	-	-	9.9
	CG (20)	8.5	1.2	1-2	-	-	-	18.2	3.7	5-6	-	-	9.7
Angelieri 2014	TG (17)	10.8	0.6	1-3	12.5	0.6	1-4	19.7	0.7	5-6	1.7	7.2	8.9
	CG (17)	11.3	0.6	1-3	12.7	0.6	2-4	18.9	2.0	5-6	1.4	6.2	7.6
Wigal 2008 (males)	TG (7)	8.7	1.3	-	9.6	1.2	-	15.2	1.5	-	0.9	5.6	6.5
	CG (7)	8.7	1.1	-	9.6	1.1	-	15.2	1.9	-	0.9	5.6	6.5
Wigal 2008 (females)	TG (15)	8.3	0.9	-	9.1	0.4	-	14.3	1.3	-	0.8	5.2	6.0
	CG (15)	8.3	1.1	-	9.2	0.3	-	14.4	1.3	-	0.9	5.2	6.1
Drosen 2018 (males)	TG (13)	12.4	0.9	-	14.2	1.2	-	20.2	1.0	-	1.8	6.0	7.8
	CG (13)	12.1	0.5	-	14.2	0.6	-	19.8	2.3	-	2.1	5.6	7.7
Alhoraibi 2017 (early)	TG (18)	11.5	0.8	1	13.1	0.8	-	16.4	1.1	-	1.6	3.3	4.9
	CG (18)	11.8	0.9	1	13.9	1.5	-	17.1	1.3	-	2.1	3.2	5.3
Alhoraibi 2017 (late)	TG (21)	13.3	0.6	2-3	15.3	0.8	-	18.4	1.0	-	2.0	3.1	5.1
	CG (21)	13.5	0.8	2-3	15.1	0.6	-	18.2	0.7	-	1.6	3.1	4.7

N, number of participants; TG, treated group; CG, control group

T1, at the start of the active phase of functional appliance therapy; T2, at the end of the active phase of functional appliance therapy; T3, long-term follow-up

SD, standard deviation; CVMS, cervical vertebral maturation stage.

Five studies evaluated the treatment effects of three removable functional appliances as follows:

■ Activator only [42];
■ A mixed group of patients treated either with the Bionator or Activator [43];
■ Frankel-2 appliance only [44–46].

Two trials evaluated respectively the effects of early treatment (mean age at start = 8.4 years [47]) and late treatment (mean age at start = 12.4 years [48]) of a fixed rigid appliance, the Herbst appliance. One study tested a fixed flexible appliance, the Forsus appliance [49]. Multi-bracket therapy was worn concurrently with functional appliance treatment in one study [49], and after functional appliance therapy in 3 trials [43, 47, 48]. A variety of appliances and retention protocols were used in the post-treatment period. All the studies compared Class II malocclusion patients treated with functional appliances to untreated Class II subjects [42–49].

Only cephalometric skeletal measurements were recorded from the 8 studies included in this review [42–49]. Soft tissue changes of both lips and chin measured on lateral cephalograms were investigated only by a report [51] of an included study [43]. Cephalometric magnifications were set at 0% [47, 48], 8% [43, 45, 49], 10% adjusted to 0% [46]. In the rest of the studies, information was not provided [42, 44]. Outcomes were assessed above 18 years in age in 5 trials (5 subgroups [43, 45, 46, 48, 49]) and at the end of growth using the cervical vertebral maturation method in 3 trials (4 subgroups [43, 45, 46]). All the studies had a post-retention period of at least 3 years (Table 3 [42–49]).

### Risk of bias within studies

The overall risk of bias ranged from moderate to critical in the included studies (Table 4). Most studies suffered bias in selection of participants and due to deviations from intended interventions [42–49]. The estimated effect can be predicted to be greater than the true effect estimate in studies with the observed selection bias [42, 43, 49]. Multi-bracket therapy, as well as retention appliances, could enhance the treatment effects of functional jaw orthopaedics or control their relapse [43, 47–49].

**Table 4:** Risk of bias for multiple outcomes within included studies, according to the risk of bias tool for non-randomised studies of interventions (ROBINS-I tool).

Bias domain	Signalling question	Wieslander and Lagerström, 1979	Pavoni et al., 2017	Falck, 1991	Freeman et al., 2009	Angelieri et al., 2014	Wigal, 2008	Drosen et al., 2018	Alhoraibi, 2017
1. Bias due to confounding	1.1	Y	Y	Y	Y	Y	Y	Y	Y
	1.2	N	N	N	N	N	N	N	N
	1.3	-	-	-	-	-	-	-	-
	1.4	PY	PY	PY	PY	PY	PY	PY	PY
	1.5	PY	PY	PY	PY	PY	PY	PY	PY
	1.6	PN	PN	PN	PN	PN	PN	PN	PN
	1.7	PY	Y	PY	PY	PY	PY	PY	PY
	1.8	PY	PY	PY	PY	PY	PY	PY	PY
Risk of bias judgement		Low	Low	Low	Low	Low	Low	Low	Low
2. Bias in selection of participants into the study	2.1	Y	PY	NI	NI	PY	NI	NI	PN
	2.2	Y	Y	-	-	Y	-	-	-
	2.3	Y	Y	-	-	Y	-	-	-
	2.4	Y	Y	Y	Y	Y	Y	Y	Y
	2.5	N	N	-	-	N	-	-	-
Risk of bias judgement		Crit	Ser	Low	Low	Ser	Low	Low	Low
3. Bias in classification of interventions	3.1	Y	Y	Y	Y	Y	Y	Y	Y
	3.2	Y	Y	Y	Y	Y	Y	Y	Y
	3.3	N	N	N	N	N	N	N	N
Risk of bias judgement		Low	Low	Low	Low	Low	Low	Low	Low
4. Bias due to deviations from intended interventions	4.1	PN	PN	N	N	N	PN	Y	PN
	4.2	-	-	-	-	-	-	Y	-
	4.3	NI	PN	Y	Y	Y	PN	PN	PN
	4.4	PY	PY	PY	PY	PY	PY	PY	PY
	4.5	PY	PY	PY	PY	PY	PY	PY	PY
	4.6	-	-	-	-	-	-	-	-
Risk of bias judgement		Low	Mod	Low	Low	Low	Mod	Ser	Mod
5. Bias due to missing data	5.1	N	Y	Y	Y	Y	N	Y	Y
	5.2	PN	PN	PN	PN	PN	PN	PN	PN
	5.3	Y	PN	PN	PN	PN	PN	PN	PN
	5.4	Y	-	-	-	-	Y	-	-
	5.5	PN	-	-	-	-	PN	-	-
Risk of bias judgement		Ser	Low	Low	Low	Low	Mod	Low	Low
6. Bias in measurement of outcomes	6.1	NI	NI	NI	NI	NI	NI	NI	NI
	6.2	NI	NI	NI	NI	NI	NI	NI	NI
	6.3	Y	Y	Y	Y	Y	Y	Y	Y
	6.4	PN	PN	PN	PN	PN	PN	PN	PN
Risk of bias judgement		Mod	Mod	Mod	Mod	Mod	Mod	Mod	Mod
7. Bias in selection of the reported result	7.1	PN	PN	PN	PN	PN	PN	PN	PN
	7.2	PY	PY	PY	PY	PY	PY	PY	PY
	7.3	N	N	N	N	N	N	N	N
Risk of bias judgement		Mod	Mod	Mod	Mod	Mod	Mod	Mod	Mod
** Overall risk of bias**		**Crit**	**Ser**	**Mod**	**Mod**	**Ser**	**Mod**	**Ser**	**Mod**

Y, yes; PY, probably yes; N, no; PN, probably no; NI, no information.

"-", not applicable or nothing to note

Mod, moderate; Ser, serious; Crit, critical.

### Results of individual studies

The main results of the included studies are reported in S5–S6 Tables.

Only one report [51] found that Bionator therapy was able to significantly alter the sagittal position of both the maxillary and mandibular soft tissue profile components. During the overall observation period, functional jaw orthopaedics with the Bionator, followed by multi-bracket appliances produced a restraining effect on the soft tissue A point (-1.8 mm, CI not reported) and a protrusive effect on the soft tissue Pg point (+2.6 mm, CI not reported).

### Synthesis of results

Seven studies (10 subgroups [42, 43, 45–49]) were included in the meta-analyses of 9 outcomes at 3 time points (Table 5). Subgroup analyses according to the type of functional appliance are presented together with their overall effects (Tables 6–7). The forest plots concerning the most clinically relevant results are reported in the main text. Other findings are set out in S3 Appendix.

**Table 5:** Details of the performed meta-analyses with tests on heterogeneity.

Outcome	Time point	Overall effect				Heterogeneity			
		N_s	MD	95% CI	P	Tau^2^	Chi^2^	P	I^2^
**Mx skeletal**									
SNA (degrees)	Age 18 +	5	-0.31	-0.83, 0.21	0.24	0.05	4.62	0.33	13%
	CVMS 5-6	4	-0.73	-1.31, -0.15	0.01	0.00	0.02	1.00	0%
	3-years +	9	-1.03	-1.88, -0.18	0.02	1.28	50.87	0.00	84%
A to N perp (mm)	Age 18 +	3	-2.41	-6.45, 1.62	0.24	12.54	140.47	0.00	99%
	CVMS 5-6	2	-0.48	-2.74, 1.77	0.67	2.41	11.49	0.00	91%
	3-years +	4	-2.24	-4.79, 0.30	0.08	6.57	164.00	0.00	98%
Co-A (mm)	Age 18 +	3	0.53	0.00, 1.05	0.05	0.00	0.65	0.72	0%
	CVMS 5-6	2	0.15	-1.16, 1.46	0.82	0.00	0.27	0.60	0%
	3-years +	6	-0.96	-2.32, 0.40	0.17	2.04	39.60	0.00	87%
**Md skeletal**									
SNB (degrees)	Age 18 +	5	0.66	0.03, 1.29	0.04	0.22	7.05	0.13	43%
	CVMS 5-6	4	0.65	-0.45, 1.74	0.25	0.89	10.25	0.02	71%
	3-years +	9	0.14	-0.48, 0.76	0.67	0.52	21.67	0.01	63%
Pg to N perp (mm)	Age 18 +	4	1.42	0.01, 2.84	0.05	1.39	10.02	0.02	70%
	CVMS 5-6	4	1.54	-0.25, 3.32	0.09	2.22	9.30	0.03	68%
	3-years +	6	0.86	-0.41, 2.13	0.18	1.80	23.00	0.00	78%
Co-Gn (mm)	Age 18 +	4	3.20	1.32, 5.08	0.00	2.61	11.89	0.01	75%
	CVMS 5-6	4	2.87	0.47, 5.26	0.02	4.38	11.57	0.01	74%
	3-years +	8	1.79	-0.05, 3.64	0.06	5.73	57.49	0.00	88%
**Mx-md skeletal**									
ANB (degrees)	Age 18 +	5	-1.00	-2.15, 0.16	0.09	1.52	35.86	0.00	89%
	CVMS 5-6	4	-1.31	-2.37, -0.24	0.02	0.97	17.21	0.00	83%
	3-years +	10	-1.11	-1.82, -0.40	0.00	1.07	57.36	0.00	84%
Wits (mm)	Age 18 +	5	-3.40	-4.45, -2.35	0.00	0.87	11.10	0.03	64%
	CVMS 5-6	4	-3.52	-5.11, -1.93	0.00	1.85	10.71	0.01	72%
	3-years +	9	-2.89	-3.64, -2.14	0.00	0.78	23.26	0.00	66%
Co-Gn/Co-A diff (mm)	Age 18 +	3	2.07	0.79, 3.35	0.00	0.64	3.99	0.14	50%
	CVMS 5-6	2	2.69	1.51, 3.86	0.00	0.00	0.49	0.48	0%
	3-years +	6	2.56	1.07, 4.05	0.00		2.64	24.57	0.00	80%

Mx skeletal, maxillary skeletal outcomes; SNA, SNA angle; A to N perp, A point to N perpendicular distance; Co-A, Co-A distance

Md skeletal, mandibular skeletal outcomes; SNB, SNB angle; Pg to N perp, Pg point to N perpendicular distance; Co-Gn, Co-Gn distance

Mx-md skeletal, maxillo-mandibular outcomes; ANB, ANB angle; Wits, Wits appraisal; Co-Gn/Co-A diff, Co-Gn/Co-A difference

Age 18 +, above 18 years of age; CVMS 5–6, at the end of growth according to the cervical vertebral maturation method; 3-years +, after a post-retention period of at least 3 years

N_s, number of studies or subgroups; MD, mean differences; 95% CI, 95% confidence intervals; P, P value.

**Table 6:** Details of the performed subgroup analysis according to the type of functional appliance (Bionator/Activator and multi-bracket appliances, Frankel-2 appliance).

Outcome	Time point	Bionator/Activator + multibracket appliances				Frankel-2 appliance			
		N_s	MD	95% CI	P	N_s	MD	95% CI	P
**Mx skeletal**									
SNA (degrees)	Age 18 +	1	-0.70	-2.20, 0.80	0.36	2	-0.70	-1.46, 0.06	0.07
	CVMS 5-6	2	-0.76	-1.67, 0.14	0.10	2	-0.70	-1.46, 0.06	0.07
	3-years +	2	-0.76	-1.67, 0.14	0.10	2	-0.70	-1.46, 0.06	0.07
A to N perp (mm)	Age 18 +	-	-	-	-	2	-0.48	-2.74, 1.77	0.67
	CVMS 5-6	-	-	-	-	2	-0.48	-2.74, 1.77	0.67
	3-years +	-	-	-	-	2	-0.48	-2.74, 1.77	0.67
Co-A (mm)	Age 18 +	-	-	-	-	2	0.15	-1.16, 1.46	0.82
	CVMS 5-6	-	-	-	-	2	0.15	-1.16, 1.46	0.82
	3-years +	-	-	-	-	2	0.15	-1.16, 1.46	0.82
**Md skeletal**									
SNB (degrees)	Age 18 +	1	1.10	-0.19, 2.39	0.09	2	1.19	0.11, 2.26	0.03
	CVMS 5-6	2	0.12	-1.74, 1.99	0.90	2	1.19	0.11, 2.26	0.03
	3-years +	2	0.12	-1.74, 1.99	0.90	2	1.19	0.11, 2.26	0.03
Pg to N perp (mm)	Age 18 +	1	2.90	1.11, 4.69	0.00	2	1.16	-2.26, 4.59	0.51
	CVMS 5-6	2	2.05	0.11, 3.99	0.04	2	1.16	-2.26, 4.59	0.51
	3-years +	2	2.05	0.11, 3.99	0.04	2	1.16	-2.26, 4.59	0.51
Co-Gn (mm)	Age 18 +	1	5.10	3.29, 6.91	0.00	2	3.18	1.31, 5.04	0.00
	CVMS 5-6	2	2.35	-3.23, 7.93	0.41	2	3.18	1.31, 5.04	0.00
	3-years +	2	2.35	-3.23, 7.93	0.41	2	3.18	1.31, 5.04	0.00
**Mx-md skeletal**									
ANB (degrees)	Age 18 +	1	-1.80	-2.74, -0.86	0.00	2	-1.82	-2.69, -0.94	0.00
	CVMS 5-6	2	-0.87	-2.64, 0.89	0.33	2	-1.82	-2.69, -0.94	0.00
	3-years +	3	-1.19	-2.41, 0.04	0.06	2	-1.82	-2.69, -0.94	0.00
Wits (mm)	Age 18 +	1	-5.40	-7.66, -3.14	0.00	2	-3.64	-5.59, -1.68	0.00
	CVMS 5-6	2	-3.45	-7.17, 0.27	0.07	2	-3.64	-5.59, -1.68	0.00
	3-years +	2	-3.45	-7.17, 0.27	0.07	2	-3.64	-5.59, -1.68	0.00
Co-Gn/Co-A diff (mm)	Age 18 +	-	-	-	-	2	2.69	1.51, 3.86	0.00
	CVMS 5-6	-	-	-	-	2	2.69	1.51, 3.86	0.00
	3-years +	-	-	-	-	2	2.69	1.51, 3.86	0.00

Mx skeletal, maxillary skeletal outcomes; SNA, SNA angle; A point to N perp, A to N perpendicular distance; Co-A, Co-A distance

Md skeletal, mandibular skeletal outcomes; SNB, SNB angle; Pg point to N perp, Pg to N perpendicular distance; Co-Gn, Co-Gn distance

Mx-md skeletal, maxillo-mandibular outcomes; ANB, ANB angle; Wits, Wits appraisal; Co-Gn/Co-A diff, Co-Gn/Co-A difference

Age 18 +, above 18 years of age; CVMS 5–6, at the end of growth according to the cervical vertebral maturation method; 3-years +, after a post-retention period of at least 3 years

N_s, number of studies or subgroups; MD, mean differences; 95% CI, 95% confidence intervals; P, P value

P_s, test for subgroup differences.

**Table 7:** Details of the performed subgroup analysis according to the type of functional appliance (Herbst, Forsus and multi-bracket appliances).

Outcome	Time point	Herbst +/- multibracket appliances				Forsus + multibracket appliances				
		N_s	MD	95% CI	P	N_s	MD	95% CI	P	P_s
**Mx skeletal**										
SNA (degrees)	Age 18 +	1	-0.60	-1.91, 0.71	0.37	1	0.40	-0.38, 1.18	0.32	0.20
	CVMS 5-6	-	-	-	-	-	-	-	-	0.92
	3-years +	3	-1.62	-3.17, -0.07	0.04	2	-0.92	-3.47, 1.62	0.48	0.77
A to N perp (mm)	Age 18 +	-	-	-	-	1	-6.30	-7.01, -5.59	0.00	0.00
	CVMS 5-6	-	-	-	-	-	-	-	-	NA
	3-years +	-	-	-	-	2	-3.99	-8.50, 0.52	0.08	0.17
Co-A (mm)	Age 18 +	-	-	-	-	1	0.60	0.03, 1.17	0.04	0.54
	CVMS 5-6	-	-	-	-	-	-	-	-	NA
	3-years +	2	-4.08	-6.03, -2.12	0.00	2	-0.40	-2.36, 1.56	0.69	0.00
**Md skeletal**										
SNB (degrees)	Age 18 +	1	-0.30	-1.69, 1.09	0.67	1	0.30	-0.27, 0.87	0.31	0.25
	CVMS 5-6	-	-	-	-	-	-	-	-	0.33
	3-years +	3	-0.41	-1.35, 0.54	0.40	2	-0.21	-1.29, 0.87	0.70	0.15
Pg to N perp (mm)	Age 18 +	-	-	-	-	1	0.90	0.17, 1.63	0.02	0.13
	CVMS 5-6	-	-	-	-	-	-	-	-	0.66
	3-years +	-	-	-	-	2	-0.06	-2.02, 1.89	0.95	0.32
Co-Gn (mm)	Age 18 +	-	-	-	-	1	1.60	0.62, 2.58	0.00	0.00
	CVMS 5-6	-	-	-	-	-	-	-	-	0.78
	3-years +	2	-1.44	-6.09, 3.22	0.55	2	2.59	0.63, 4.55	0.01	0.35
**Mx-md skeletal**										
ANB (degrees)	Age 18 +	1	-0.40	-1.32, 0.52	0.40	1	0.60	-0.01, 1.21	0.05	0.00
	CVMS 5-6	-	-	-	-	-	-	-	-	0.35
	3-years +	3	-1.48	-2.72, -0.25	0.02	2	0.17	-0.80, 1.14	0.73	0.02
Wits (mm)	Age 18 +	1	-2.40	-4.11, -0.69	0.01	1	-2.70	-3.53, -1.87	0.00	0.13
	CVMS 5-6	-	-	-	-	-	-	-	-	0.93
	3-years +	3	-1.74	-2.66, -0.81	0.00	2	-3.10	-3.78, -2.42	0.00	0.09
Co-Gn/Co-A diff (mm)	Age 18 +	-	-	-	-	1	1.00	-0.32, 2.32	0.14	0.06
	CVMS 5-6	-	-	-	-	-	-	-	-	NA
	3-years +	2	1.63	-0.09, 3.34	0.06	2	2.97	-0.85, 6.79	0.13	0.58

Mx skeletal, maxillary skeletal outcomes; SNA, SNA angle; A point to N perp, A to N perpendicular distance; Co-A, Co-A distance

Md skeletal, mandibular skeletal outcomes; SNB, SNB angle; Pg point to N perp, Pg to N perpendicular distance; Co-Gn, Co-Gn distance

Mx-md skeletal, maxillo-mandibular outcomes; ANB, ANB angle; Wits, Wits appraisal; Co-Gn/Co-A diff, Co-Gn/Co-A difference

Age 18 +, above 18 years of age; CVMS 5–6, at the end of growth according to the cervical vertebral maturation method; 3-years +, after a post-retention period of at least 3 years

N_s, number of studies or subgroups; MD, mean differences; 95% CI, 95% confidence intervals; P, P value

P_s, test for subgroup differences.

#### Maxillary/Upper jaw changes

**Fig 2:**
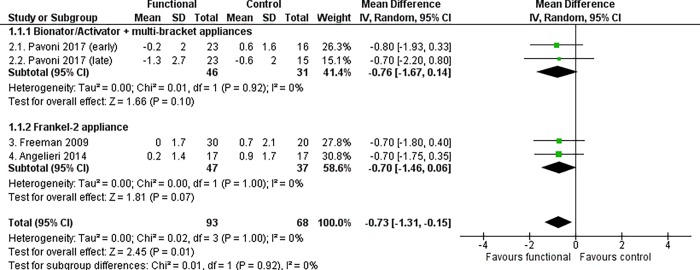
Meta-analysis; Outcome: SNA angle; Time point: End of growth according to the CVM method.

It was found that functional appliances produced a statistically significant reduction in the angular position of the maxilla (SNA angle) at the end of growth according to the CVM method (MD -0.73°, 95% CI -1.31 to -0.15, P = 0.01, I^2^ = 0%, 4 studies [Fig 2]) and after a post-retention period of at least 3 years (MD -1.03°, 95% CI -1.88 to -0.18, P = 0.02, I^2^ = 84%, 9 studies [Table 5]).

The most clinically relevant maxillary effects were produced by fixed functional appliances: the Herbst appliance (Co-A distance at least 3 years after retention, MD -4.08 mm, 95% CI -6.03 to -2.12, P < 0.0001, I^2^ = 0%, 2 studies [Table 7]) and the Forsus device, in combination with multi-bracket therapy (A to N perpendicular distance above 18 years of age, MD -6.30 mm, 95% CI -7.01 to -5.59, P < 0.00001, I^2^ = Not applicable, 1 study [Table 7]).

#### Mandibular/Lower jaw changes

**Fig 3:**
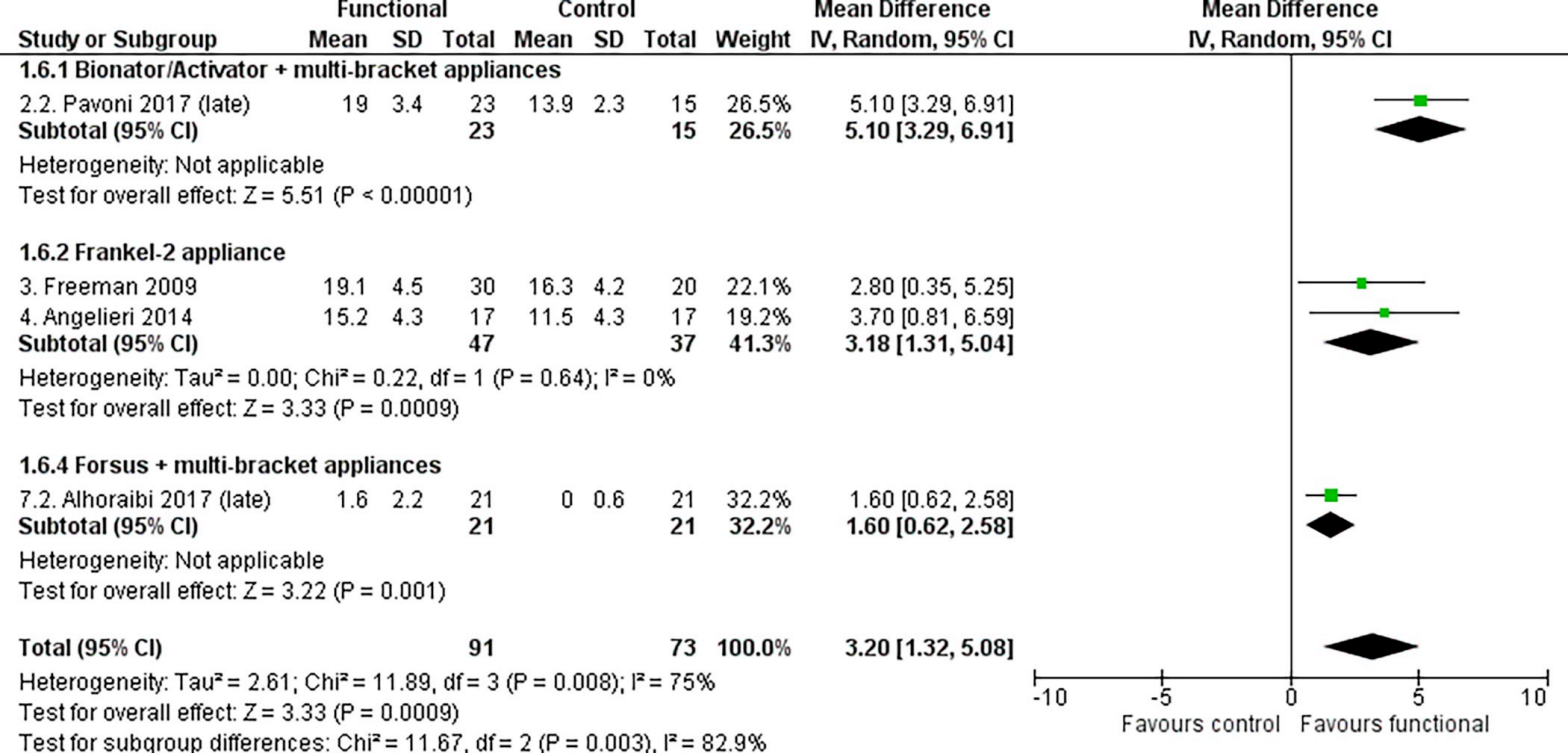
Meta-analysis; Outcome: Co-Gn distance; Time point: Above 18 years of age.

**Fig 4:**
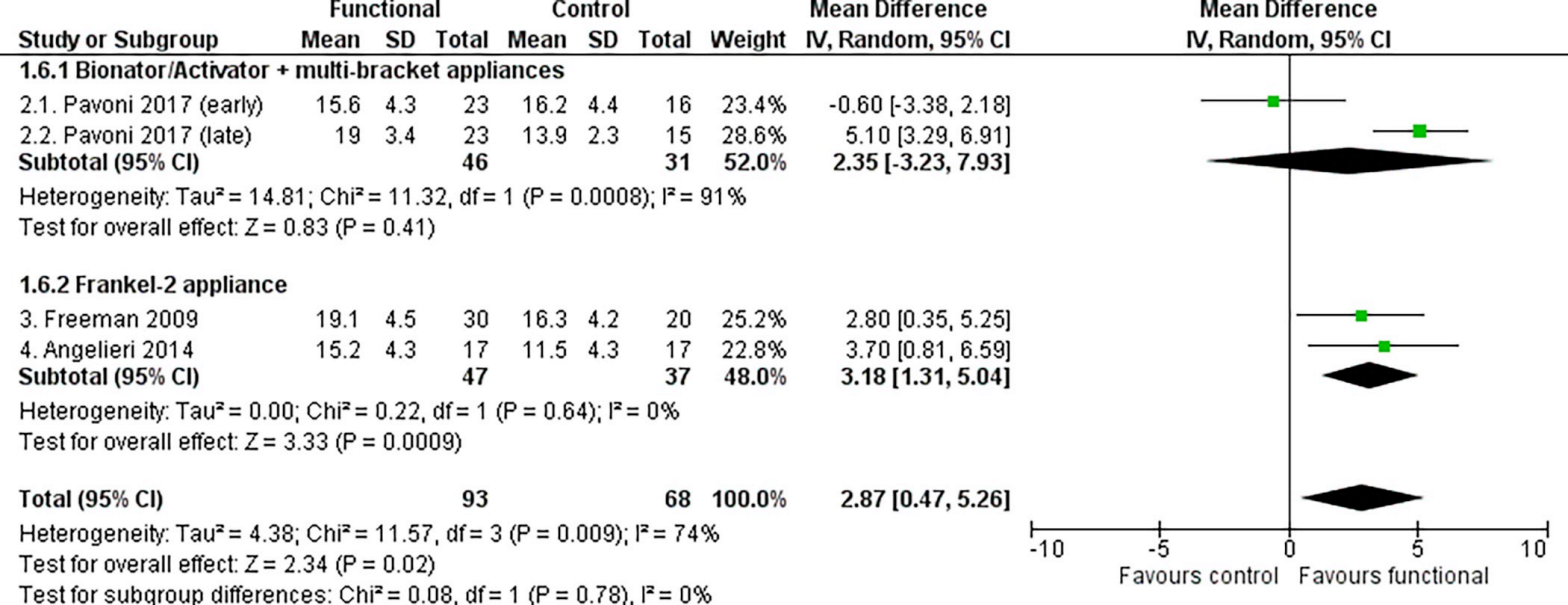
Meta-analysis; Outcome: Co-Gn distance; Time point: End of growth according to the CVM method.

Treated patients showed a statistically significant increase in the mandibular length (Co-Gn distance) compared to untreated subjects, at both primary time points. The increase in the mandibular growth was 3.20 mm in patients aged 18 years and above (95% CI 1.32 to 5.08, P = 0.0009, I^2^ = 75%, 4 studies [Fig 3]) and 2.87 mm at the end of growth according to the CVM method (95% CI 0.47 to 5.26, P = 0.02, I^2^ = 74%, 4 studies [Fig 4]).

The angular improvement of the mandibular projection was significant above 18 years of age (SNB angle, MD 0.66°, 95% CI 0.03 to 1.29, P = 0.04, I^2^ = 43%, 5 studies [Table 5]), however the linear improvement of the same outcome was not significant at any time point (Pg to N perpendicular distance above 18 years of age, MD 1.42 mm, 95% CI 0.01 to 2.84, P = 0.05, I^2^ = 70%, 4 studies [Table 5]).

Removable functional appliances produced greater treatment effects than fixed devices. The greatest significant increase in the mandibular growth (Co-Gn distance) above 18 years of age was observed in a single study [43], in which a mixed subgroup of patients was treated either with the Bionator or Activator during puberty (MD 5.10 mm, 95% CI 3.29 to 6.91, P < 0.00001, I^2^ = Not applicable, 1 study [Table 6]). This group also showed a statistically significant improvement of the sagittal projection of the mandible (Pg to N perpendicular distance, MD 2.90 mm, 95% CI 1.11 to 4.69, P = 0.001, I^2^ = Not applicable, 1 study [Table 6]), although the test for subgroup differences was not significant (P = 0.13, I^2^ = 51.5%).

#### Maxillo-mandibular changes

**Fig 5:**
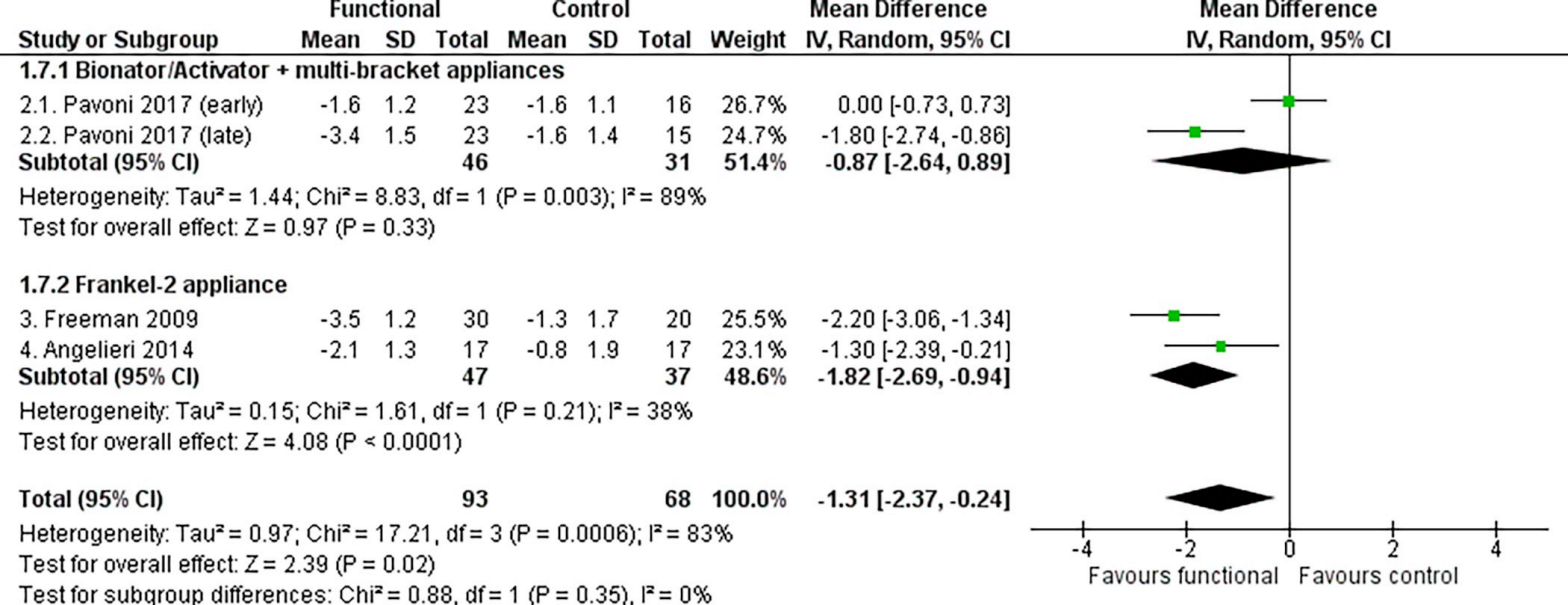
Meta-analysis; Outcome: ANB angle; Time point: End of growth according to the CVM method.

**Fig 6:**
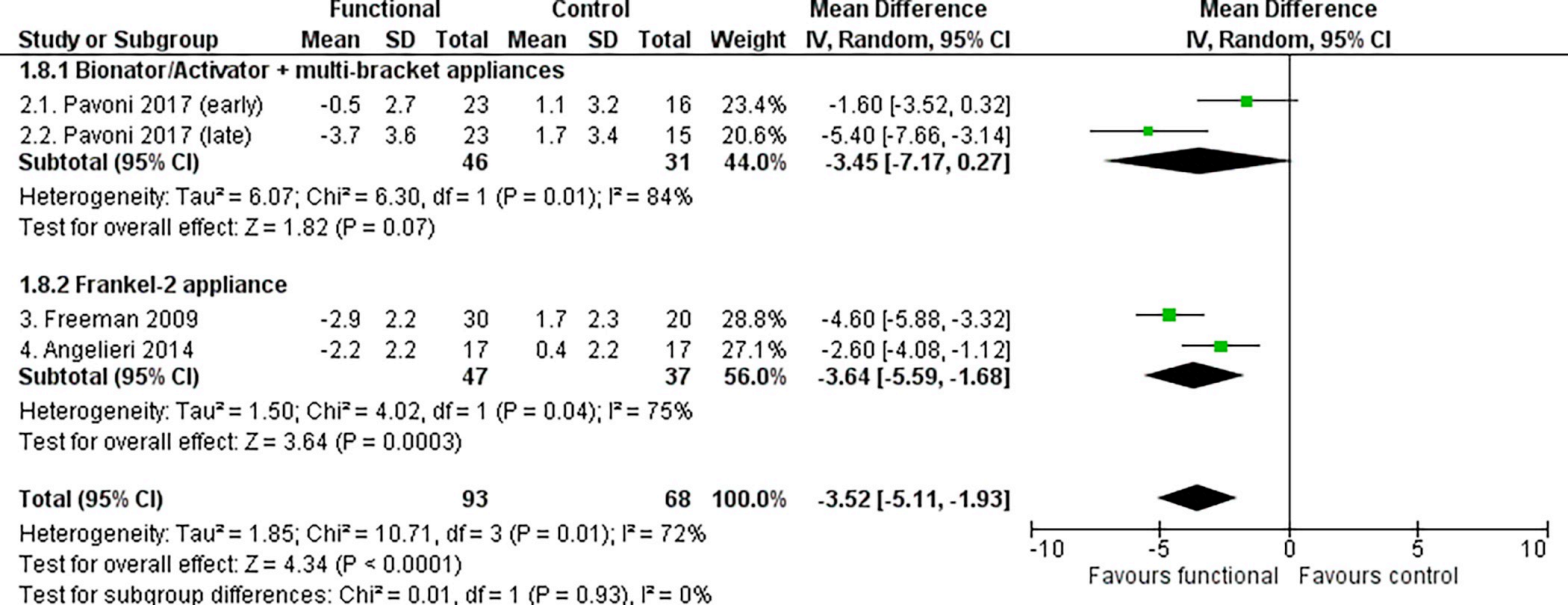
Meta-analysis; Outcome: Wits appraisal; Time point: End of growth according to the CVM method.

**Fig 7:**
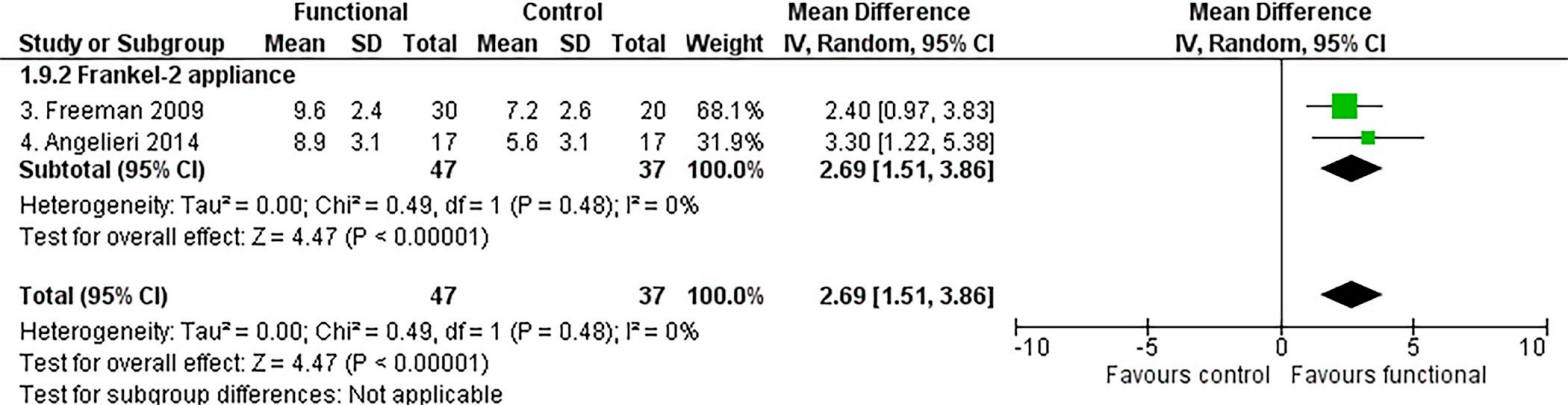
Meta-analysis; Outcome: Co-Gn/Co-A difference; Time point: End of growth according to the CVM method.

Functional appliance therapy produced a statistically significant improvement of the mutual relationship between the maxilla and mandible, at almost all time points. The most clinically relevant maxillo-mandibular changes were recorded at the end of growth according to the CVM method, when treated patients exhibited an improvement in both angular and linear measurements relative to the controls (ANB angle, MD -1.31°, 95% CI -2.37 to -0.24, P = 0.02, I^2^ = 83%, 4 studies [Fig 5]; Wits appraisal, MD -3.52 mm, 95% CI -5.11 to -1.93, P < 0.0001, I^2^ = 72%, 4 studies [Fig 6]; Co-Gn/Co-A difference, MD 2.69 mm, 95% CI 1.51, 3.86, P < 0.0001, I^2^ = 0%, 2 studies [Fig 7]).

The Frankel-2 appliance worn alone improved all skeletal maxillo-mandibular outcomes regardless of the time point chosen. The statistically significant improvement of the ANB angle, Wits appraisal and Co-Gn/Co-A difference were respectively -1.82° (95% CI -2.69 to -0.94, P < 0.0001, I^2^ = 38%, 2 studies [Fig 5]), -3.64 mm (95% CI -5.59 to -1.68, P = 0.0003, I^2^ = 75%, 2 studies [Fig 6), and 2.69 mm (95% CI 1.51 to 3.86, P < 0.00001, I^2^ = 0%, 2 studies [Fig 7]).

### Additional analysis

Few statistically significant differences were found among the subgroups analysed (Tables 8–9, S3 Appendix). Early treatment with functional appliances (commencing in children aged between 7 and 11 years) produced a greater improvement of the angular antero-posterior position of the maxilla (SNA angle) and the relationship between the two jaws (ANB angle) than late treatment (beginning in adolescents aged between 12 and 16 years).

**Table 8:** Details of the performed subgroup analyses, according to gender, beginning of the functional appliance therapy and post-retention period duration.

Outcome	Subgroups	Overall effect				Heterogeneity			
		N_s	MD	95% CI	P	Tau^2^	Chi^2^	P	I^2^
**Males Vs females**									
SNA (degrees)	Males	2	-0.85	-1.96, 0.27	0.14	0.00	0.50	0.48	0%
	Females	1	-3.20	-5.25, -1.15	0.00	NA			
Total (95% CI)		3	-1.62	-3.17, -0.07	0.04	1.02	4.42	0.11	55%
Subgroup differences:							3.92	0.05	75%
Co-Gn (mm)	Males	1	1.30	-2.71, 5.31	0.52	NA			
	Females	1	-3.50	-5.41, -1.59	0.00	NA			
Total (95% CI)		2	-1.44	-6.09, 3.22	0.55	8.95	4.49	0.03	78%
Subgroup differences:							4.49	0.03	78%
ANB (degrees)	Males	2	-1.26	-3.11, 0.60	0.18	1.41	4.55	0.03	78%
	Females	1	-2.00	-3.11, -0.89	0.00	NA			
Total (95% CI)		3	-1.48	-2.72, -0.25	0.02	0.84	6.92	0.03	71%
Subgroup differences:							0.45	0.50	0%
**Early Vs late treatments according to age**									
SNA (degrees)	7 < age < 11	7	-1.34	-2.11, -0.57	0.00	0.66	20.39	0.00	71%
	12 < age < 16	2	0.04	-0.90, 0.98	0.93	0.20	1.66	0.20	40%
Total (95% CI)		9	-1.03	-1.88, -0.18	0.02	1.28	50.87	0.00	84%
Subgroup differences:							4.99	0.03	80%
Co-Gn (mm)	7 < age < 11	7	1.81	-0.61, 4.23	0.14	9.08	55.68	0.00	89%
	12 < age < 16	1	1.60	0.62, 2.58	0.00	NA			
Total (95% CI)		8	1.79	-0.05, 3.64	0.06	5.73	57.49	0.00	88%
Subgroup differences:							0.02	0.88	0%
ANB (degrees)	7 < age < 11	8	-1.43	-2.07, -0.79	0.00	0.61	26.11	0.00	73%
	12 < age < 16	2	0.16	-0.81, 1.13	0.74	0.34	3.13	0.08	68%
Total (95% CI)		10	-1.11	-1.82, -0.40	0.00	1.07	57.36	0.00	84%
Subgroup differences:							7.15	0.01	86%
**Early Vs late treatments according to the cervical vertebral maturation method**									
SNA (degrees)	CVSM 1-2	2	-1.61	-2.96, -0.25	0.02	0.80	5.40	0.02	81%
	CVSM 2-3	2	0.04	-0.97, 1.05	0.93	0.23	1.63	0.20	39%
Total (95% CI)		4	-0.85	-2.35, 0.64	0.26	2.06	40.60	0.00	93%
Subgroup differences:							3.67	0.06	73%
Co-Gn (mm)	CVSM 1-2	2	1.71	-2.39, 5.80	0.41	7.67	7.66	0.01	87%
	CVSM 2-3	2	3.26	-0.16, 6.69	0.06	5.57	11.11	0.00	91%
Total (95% CI)		4	2.61	0.76, 4.47	0.01	2.85	19.83	0.00	85%
Subgroup differences:							0.33	0.57	0%
ANB (degrees)	CVSM 1-2	2	-0.15	-0.73, 0.43	0.62	0.00	0.43	0.51	0%
	CVSM 2-3	2	-0.57	-2.92, 1.78	0.63	2.72	17.66	0.00	94%
Total (95% CI)		4	-0.36	-1.33, 0.61	0.47	0.81	18.10	0.00	83%
Subgroup differences:							0.12	0.73	0%
**3-4 Vs 5-10 years after active functional appliance therapy**									
SNA (degrees)	3-4 years	2	-0.92	-3.47, 1.62	0.48	3.29	36.06	0.00	97%
	5-10 years	7	-0.90	-1.40, -0.40	0.00	0.00	5.72	0.46	0%
Total (95% CI)		9	-1.03	-1.88, -0.18	0.02	1.28	50.87	0.00	84%
Subgroup differences:							0.00	0.98	0%
Co-Gn (mm)	3-4 years	2	2.59	0.63, 4.55	0.01	1.73	7.46	0.01	87%
	5-10 years	6	1.46	-1.63, 4.55	0.35	13.01	46.89	0.00	89%
Total (95% CI)		8	1.79	-0.05, 3.64	0.06	5.73	57.49	0.00	88%
Subgroup differences:							0.37	0.55	0%
ANB (degrees)	3-4 years	3	-0.53	-2.06, 1.00	0.50	1.67	25.46	0.00	92%
	5-10 years	7	-1.37	-2.11, -0.63	0.00	0.74	24.20	0.00	75%
Total (95% CI)		10	-1.11	-1.82, -0.40	0.00	1.07	57.36	0.00	84%
Subgroup differences:							0.94	0.33	0%

SNA, SNA angle; Co-Gn, Co-Gn distance; ANB, ANB angle

7 < age < 11; early treatments, commencing in children aged between 7 and 11 years; 12 < age < 16; late treatments, beginning in adolescents aged between 12 and 16 years

CVSM 1–2; early treatments, with patients presenting with Cervical Vertebral Maturation Stage (CVMS) 1 or 2 at the first observation; CVSM 2–3, late treatments, with subjects presenting with CVMS 2 or 3

N_s, number of studies or subgroups; MD, mean differences; 95% CI, 95% confidence intervals; P, P value.

**Table 9:** Details of the performed sensitivity analyses according to study quality assessment.

Outcome	Subgroups	Overall effect				Heterogeneity			
		N_s	MD	95% CI	P	Tau^2^	Chi^2^	P	I^2^
SNA (degrees)	Low-mod	5	-1.34	-2.72, 0.05	0.06	2.03	41.62	0.00	90%
	Crit-ser	4	-0.71	-1.31, -0.10	0.02	0.00	0.05	1.00	0%
Co-Gn (mm)	Low-mod	5	1.19	-1.17, 3.54	0.32	5.99	41.55	0.00	90%
	Crit-ser	3	2.83	-0.57, 6.23	0.10	7.39	11.36	0.00	82%
ANB (degrees)	Low-mod	5	-1.20	-2.51, 0.11	0.07	1.96	39.09	0.00	90%
	Crit-ser	5	-1.05	-1.84, -0.26	0.01	0.61	16.90	0.00	76%

SNA, SNA angle; Co-Gn, Co-Gn distance; ANB, ANB angle

Mod, moderate; Ser, serious; Crit, critical.

N_s, number of studies or subgroups; MD, mean differences; 95% CI, 95% confidence intervals; P, P value.

Sensitivity analyses revealed that, if only studies with low and moderate risk of bias were considered, differences in the most clinically important outcomes (SNA angle, Co-Gn distance, ANB angle) were not statistically significant (Table 9).

### Risk of bias across studies

The protocol of the included studies was not retrieved in the Clinical Trial Register, thus outcome reporting bias could not be assessed. Due to the limited number of included studies, an evaluation for the existence of reporting bias (including publication bias) was not possible [40].

The GRADE assessment for all the outcomes at primary time points were rated as being ‘very low’ (Table 10), except for the Co-A distance when patients were 18 or older (‘low’), and Co-Gn/Co-A difference above the age of 18 (‘low’) and at the end of growth (‘moderate’). Since the included studies were observational, evidence supporting estimates of the intervention effects started to be rated as low-quality. The evidence was down rated for most of the outcomes, as a direct result of the risk of bias and inconsistency of included trials [41].

**Table 10:** Details for the GRADE assessment of the primary outcomes.

Outcome	RB	IC	IN	IM	Overall certainty of evidence	No. part. (studies)		Anticipated absolute effects	
N_C						N_T	Risk with No treatment	Risk with Functional appliances
**Above 18 years of age**									
SNA	S	NS	NS	S	⨁◯◯◯	190 (5)		The mean ranged from **-0.6 to 0.9** degrees	MD **0.31 degrees lower** (0.83 lower to 0.21 higher)
					VERY LOW	86	104		
A to N perp	NS	S	NS	S	⨁◯◯◯	126 (3)		The mean ranged from **0.1 to 0.9** mm	MD **2.41 mm lower** (6.45 lower to 1.62 higher)
					VERY LOW	58	68		
Co-A	NS	NS	NS	NS	⨁⨁◯◯	126 (3)		The mean ranged from **0.6 to 9.6** mm	MD **0.53 mm higher** (0.00 higher to 1.05 higher)
					LOW	58	68		
SNB	S	NS	NS	NS	⨁◯◯◯	190 (5)		The mean ranged from **1.0 to 2.2** degrees	MD **0.66 degrees higher** (0.03 higher to 1.29 higher)
					VERY LOW	86	104		
Pg to N perp	S	S	NS	NS	⨁◯◯◯	164 (4)		The mean ranged from **0.9 to 3.6** mm	MD **1.42 mm higher** (0.01 higher to 2.84 higher)
					VERY LOW	73	91		
Co-Gn	S	S	NS	NS	⨁◯◯◯	164 (4)		The mean ranged from **0.0 to 16.3** mm	MD **3.20 mm higher** (1.32 higher to 5.08 higher)
					VERY LOW	73	91		
ANB	S	S	NS	S	⨁◯◯◯	190 (5)		The mean ranged from **-1.6 to -0.8** degrees	MD **1 degrees lower** (2.15 lower to 0.16 higher)
					VERY LOW	86	104		
Wits	S	S	NS	NS	⨁◯◯◯	190 (5)		The mean ranged from **0.4 to 1.7** mm	MD **3.40 mm lower** (4.45 lower to 2.35 lower)
					VERY LOW	86	104		
Co-Gn/Co-A diff	NS	NS	NS	NS	⨁⨁⨁◯	126 (3)		The mean ranged from **-0.6 to 7.2** mm	MD **2.07 mm higher** (0.79 higher to 3.35 higher)
					MODERATE	58	68		
**At the end of growth according to the cervical vertebral maturation method**									
SNA	S	NS	NS	NS	⨁◯◯◯	161 (4)		The mean ranged from **-0.6 to 0.9** degrees	MD **0.73 degrees lower** (1.31 lower to 0.15 lower)
					VERY LOW	68	93		
A to N perp	S	S	NS	S	⨁◯◯◯	84 (2)		The mean ranged from **0.1 to 0.9** mm	MD **0.48 mm lower** (2.74 lower to 1.77 higher)
					VERY LOW	37	47		
Co-A	S	NS	NS	S	⨁◯◯◯	84 (2)		The mean ranged from **5.7 to 9.6** mm	MD **0.15 mm higher** (1.16 lower to 1.46 higher)
					VERY LOW	37	47		
SNB	S	S	NS	S	⨁◯◯◯	161 (4)		The mean ranged from **1.0 to 2.2** degrees	MD **0.65 degrees higher** (0.45 lower to 1.74 higher)
					VERY LOW	68	93		
Pg to N perp	S	S	NS	S	⨁◯◯◯	161 (4)		The mean ranged from **2.8 to 3.6** mm	MD **1.54 mm higher** (0.25 lower to 3.32 higher)
					VERY LOW	68	93		
Co-Gn	S	S	NS	NS	⨁◯◯◯	161 (4)		The mean ranged from **11.5 to 16.3** mm	MD **2.87 mm higher** (0.47 higher to 5.26 higher)
					VERY LOW	68	93		
ANB	S	S	NS	NS	⨁◯◯◯	161 (4)		The mean ranged from **-1.6 to -0.8** degrees	MD **1.31 degrees lower** (2.37 lower to 0.24 lower)
					VERY LOW	68	93		
Wits	S	S	NS	NS	⨁◯◯◯	161 (4)		The mean ranged from **0.4 to 1.7** mm	MD **3.52 mm lower** (5.11 lower to 1.93 lower)
					VERY LOW	68	93		
Co-Gn/Co-A diff	S	NS	NS	NS	⨁⨁◯◯	84 (2)		The mean ranged from **5.6 to 7.2** mm	MD **2.69 mm higher** (1.51 higher to 3.86 higher)
					LOW	37	47		

SNA, SNA angle; A to N perp, A point to N perpendicular distance; Co-A, Co-A distance

SNB, SNB angle; Pg to N perp, Pg point to N perpendicular distance; Co-Gn, Co-Gn distance

ANB, ANB angle; Wits, Wits appraisal; Co-Gn/Co-A diff, Co-Gn/Co-A difference

RB, risk of bias; IC, inconsistency; IN, indirectness; IM, imprecision

No. part., number of participants; N_C, number of not treated subjects; N_T, number of treated patients.

S, serious; NS, not serious

All studies were observational studies.

## Discussion

### Summary of evidence

The results demonstrated that functional appliances, worn alone or in combination with multi-bracket therapy, produced an improvement of the maxillo-mandibular relationship at almost all time points. The improvement was around -1 degree for the angular measurement (ANB angle) and between -3.5 and 2.5 mm for the linear outcomes (Wits appraisal, Co-Gn/Co-A difference). The decrease in the ANB angle and Wits appraisal was consistent with that reported in previous systematic reviews on the effects of functional appliances in the short- [6, 21, 22, 24, 26, 28] and long-term [28].

In agreement with previous reviews [7, 21, 24], a restraint of maxillary growth (SNA angle, -1 degree) was observed in included studies. Above 18 years of age or at the end of growth according to the cervical vertebral maturation method [20], the increase in the mandibular length (Co-Gn distance) was approximately 3 mm greater in the treated patients compared to that in untreated subjects. Similar results were found in the subgroups of adolescents studied by Perinetti et al. [6, 22]. However, the improvement of the position of the mandible was negligible or not significant, as inferred from results of its measurements (SNB angle, Pg to N perpendicular). During growth, the mandible is translated downward and forward, while at the same time it increases in size by growing upward and backward [12, 14]. Vertical growth can reduce the effects of the increase in mandibular length on its projection.

According to the GRADE Working Group, the quality of evidence was ‘very low’ for most of the outcomes at both primary time points. Most of the studies received a very low rating, because of their risk of bias and inconsistency [41].

Overall, the clinical significance of these findings was limited. Several approaches were described to establish if the ‘statistically significant’ differences were also ‘clinically important’. The small or minimal clinical important, moderate and large effects were conventionally defined as half, one, and two standard deviations of the normal values, respectively [54]. According to these thresholds, functional appliances produced only small clinically significant changes in the linear maxillo-mandibular measurements (Wits appraisal, Co-Gn/Co-A difference) and in the mandibular length (Co-Gn distance).

### Strengths and limitations

Strengths of the present systematic review were in the efforts made to respect rigorous standards for quality and reduce risk of bias: original research question; unrestricted electronic search of 24 databases and additional manual searches; pre-defined and unambiguous eligibility criteria with rationale; adjustment for magnified linear measurements; 3 time points evaluated with rationale; pre-defined and broad additional analyses.

However, limitations occurred at some levels. Although both randomised and non-randomised controlled studies were sought, only retrospective controlled clinical trials were retrieved with negative consequences on the quality of evidence of the effect estimates. It needs to be noted that only long-term studies were considered eligible. The whole observational periods of included trials ranged from 4.7 to 10.2 years.

Participants were eligible regardless of their baseline disease severity. The antero-posterior relationship between the two arches or jaws affects the amount of advancement produced by functional appliances, therefore this could influence the treatment effects. The greater the space created between the upper and lower front teeth is, the more protruded position of the mandible can be achieved. Different classifications of malocclusion also bring into question the applicability of results.

Any type of functional appliance, worn alone or in combination with multi-bracket therapy, was included. As anticipated, multi-bracket therapy, as well as retention appliances, could enhance the treatment effects of functional jaw orthopaedics or control their relapse. Moreover, trials with historical untreated controls from growth studies showed larger treatment effects compared to trials with untreated controls from clinical archives [55].

Other limitations concerned the evaluated outcomes. The present systematic review mainly assessed cephalometric skeletal measurements which can be considered as ‘clinically important outcomes’. The effects of functional appliances on the soft-tissue facial structures were searched, but few results were found. Multiple related outcomes were also analysed. In fact, the ANB angle is defined as the difference between the SNA and SNB angles, whilst the Co-Gn/Co-A difference is defined as the total mandibular length (Co-Gn) minus Co-A distance. The greater the number of outcomes, the higher the chance of finding a false positive result [56]. Cephalometric magnification was not reported or retrieved in 2 studies [42, 44]. Linear measurements of these studies were excluded from meta-analyses. The impact of dental movements on the skeletal measurements cannot be examined further, as the objective of this systematic review was to assess the skeletal effects produced by functional appliances in the long-term.

With regards to time points, two alternative methods were used to define the completion of growth. Each of these methods is affected by some limitations. The age threshold of 18 years, as reported in one included trial [48], was chosen to maximise the data available. In studies of long duration with several periods of follow-up, the Cochrane Collaboration recommends to select a single time point and analyse only data at this time [30]. Some investigations reported that growth continues up to 21 years of age [15] or more [16–18]. However, above 18 years of age, most changes in the mandibular growth (Co-Gn distance) appear to be as non-clinically significant (mean change = 0.1 mm per year [17, 18]). None of the included trials evaluated the treatment effects of functional appliances in patients aged at least 21 years old. The cervical vertebral maturation method was also employed. The accuracy of this method is questionable. No skeletal maturity indicator may be considered to have a full diagnostic reliability in the identification of the phases of mandibular growth [57]. All the studies had a post-retention period of at least 3 years, so that a sufficient post-retention period after the functional appliance therapy could be guaranteed [42–49].

### Implications for practice

Based on results of this review, weak recommendations can be provided on the long-term effects of functional appliances in treated versus untreated Class II subjects. There is a very low quality evidence that functional appliance therapy produced an improvement of skeletal Class II malocclusion at the end of growth and at least 3 years after retention. Treated patients exhibited an increase in the mandibular length compared to untreated subjects, although with marginal clinical significance.

### Implications for research

Further high quality primary studies are needed to confirm or reject the findings of this review. Randomised controlled trials comparing treated patients to untreated subjects (no historical controls) should be carried out. A consensus should be formed on the clinically important measurements to be used for the inclusion in the study and assessment of the effects. Few linear measurements for the position of the maxilla and mandible, the relationship between these jaws, seem to be more appropriate because of their influence on the soft tissue measurements. Patient important outcomes, such as perceived attractiveness, self-esteem and oral health-related quality of life, should be assessed as well.

## Conclusions

Functional appliances, worn alone or in combination with multi-bracket therapy, may be effective in correcting skeletal Class II malocclusion in the long-term. The increase in the mandibular length may contribute to the improvement of the maxillo-mandibular relationship, although it brought about a negligible or non-significant improvement of the mandibular projection. The quality of evidence was ‘very low’ for most of the outcomes at both primary time points; the clinical significance of these findings was limited. Further randomised controlled trials evaluating clinically and patient important outcomes are needed to confirm or reject the findings of this review.

## Differences between protocol and review

The data extracted were not preliminarily annualised to minimize heterogeneity related to the observation period variability. Annualised changes (mean differences divided by the duration of the whole observational period) seemed to be inappropriate to evaluate the treatment effects in the long-term. If an appliance produced a certain amount of improvement in a given period (reported as degrees/year or mm/year), it does not mean that the device could cause the established improvement for each year of treatment.

An adjustment for magnified linear measurements was introduced to avoid distorted analyses.

## Supporting information

S1 Table PRISMA (Preferred Reporting Items for Systematic reviews and Meta-Analyses) 2009 Checklist. (PDF)

S2 Table Name of the search source, date range, search platform/provider and link of all databases that were used. (PDF)

S3 Table Search strategy and corresponding results for all databases. (PDF)

S4 Table Studies excluded with corresponding main reason of exclusion. (PDF)

S5 Table Results during the overall observational period for each outcome included in the meta-analysis. (PDF)

S6 Table Results during the overall observational period for each outcome excluded by the meta-analysis. (PDF)

S1 Appendix Eligibility criteria with rationale. (PDF)

S2 Appendix References to studies excluded from this review. (PDF)

S3 Appendix The forest plots concerning redundant or non-statistically significant results. (PDF)
