# Supplementary material for: Long-term effects of functional appliances in treated versus untreated patients with Class II malocclusion: A systematic review and meta-analysis
Source: PLoS One. 2019 Sep 6;14(9):e0221624. doi: 10.1371/journal.pone.0221624 (PMC6730901; doi:10.1371/journal.pone.0221624)
Supplement: S2 Table — (PDF) [file pone.0221624.s002.pdf]

**S2 Table. Name of the search source, date range, search platform/provider and link of all databases that were used.**

| Database | Search platform or provider; date range | Link |
|---|---|---|
| **Bibliographic databases** | | |
| **MEDLINE, EMBASE, CENTRAL** | | |
| MEDLINE | Pubmed; 1946 - 13th March 2018 | https://www.ncbi.nlm.nih.gov/pubmed/advanced |
| EMBASE | OVID; 1974 - 15th March 2018 | https://www.embase.com/search/advanced |
| Cochrane Central Register of Controlled Trials (CENTRAL) | Cochrane Library; 1993 - 13th March 2018 | http://onlinelibrary.wiley.com/cochranelibrary/search/ |
| **National and regional databases** | | |
| Latin America and the Caribbean (LILACS) | Biblioteca Regional de Medicina (BIREME), Pan American Health Organization (PAHO), WHO; 1982 - 13th March 2018 | http://pesquisa.bvsalud.org/portal/advanced/?lang=en |
| **General search engines** | | |
| Google Scholar | 2004 - 14th March 2018 | https://scholar.google.it/ |
| Turning Research into Practice (TRIP) database | 1997 - 14th March 2018 | https://www.tripdatabase.com/ |
| **Citation indexes** | | |
| Web of Science Core Collection - Science Citation Index / Science Citation Index Expanded | Web of Science; 1945 - 15th March 2018 | https://apps.webofknowledge.com/WOS_GeneralSearch_input.do?product=WOS&search_mode=GeneralSearch |
| Scopus | Elsevier; 2004 - 13th March 2018 | https://www.scopus.com/ |
| **Dissertation and theses databases** | | |
| ProQuest Dissertations & Theses Global | 1938 - 15th March 2018 | https://search.proquest.com/pqdtglobal/advanced |
| ProQuest Dissertations and Theses – UK & Ireland | 1950 - 15th March 2018 | https://search.proquest.com/pqdtuk/advanced |
| **Grey literature databases** | | |
| OpenGrey, formerly System for Information on Grey Literature (SIGLE) | 1993 - 13th March 2018 | http://www.opengrey.eu/ |
| **Journals and other non-bibliographic database sources** | | |
| **Hand-searching** | | |
| American Journal of Orthodontics and Dentofacial Orthopaedics | 1915 - 16th March 2018 | https://www.ajodo.org/ |
| Angle Orthodontist | 1931 - 16th March 2018 | https://www.angle.org/ |
| Australian Journal of Orthodontics | 2010 - 16th March 2018 | https://www.aso.org.au/australasian-orthodontic-journal |
| European Journal of Orthodontics | 1979 - 16th March 2018 | https://academic.oup.com/ejo |
| Journal of Clinical Orthodontics | 1967 - 16th March 2018 | https://www.jco-online.com/ |
| Journal of Orthodontics | 1973 - 16th March 2018 | https://www.tandfonline.com/loi/yjor20 |
| Orthodontics & Craniofacial Research | 1998 - 16th March 2018 | https://onlinelibrary.wiley.com/journal/16016343 |
| Progress in Orthodontics | 2013 - 16th March 2018 | https://progressinorthodontics.springeropen.com/ |
| Seminars in Orthodontics | 1995 - 16th March 2018 | https://www.semortho.com/ |
| **Tables of contents** | | |
| British Library Direct | 1930 - 16th March 2018 | https://ondemand.bl.uk/onDemand/home |
| Current Contents Connect – Clinical Medicine | Web of Science; 1998 - 15th March 2018 | https://apps.webofknowledge.com/CCC_GeneralSearch_input.do?product=CCC&search_mode=GeneralSearch |
| Scientific Electronic Library Online (SciELO) Citation Index | Web of Science; 1997 - 15th March 2018 | https://apps.webofknowledge.com/SCIELO_GeneralSearch_input.do?product=SCIELO&search_mode=GeneralSearch |

**S2 Table (continued). Name of the search source, date range, search platform/provider and link of all databases that were used.**

| Database | Search platform or provider; date range | Link |
|---|---|---|
| **Conference abstracts or proceedings** | | |
| BIOSIS Citation Index | Web of Science; 1969 - 15th March 2018 | https://apps.webofknowledge.com/BCI_GeneralSearch_input.do?product=BCI&search_mode=GeneralSearch |
| Web of Science Core Collection – Conference Proceedings Citation Index – Science | Web of Science; 1990 - 15th March 2018 | https://apps.webofknowledge.com/WOS_GeneralSearch_input.do?product=WOS&search_mode=GeneralSearch |
| ISI Proceedings | 2004 - 16th March 2018 | http://www.proceedings.com/ |
| **Other reviews, guidelines and reference lists as sources of studies** | | |
| Cochrane Database of Systematic Reviews (CDSR) | Cochrane Library; 1993 - 13th March 2018 | http://onlinelibrary.wiley.com/cochranelibrary/search/ |
| Database of Abstracts of Reviews of Effects (DARE) | Cochrane Library; 1993 - 13th March 2018 | http://onlinelibrary.wiley.com/cochranelibrary/search/ |
| Health Technology Assessment Database (HTA Database) | Cochrane Library; 1993 - 13th March 2018 | http://onlinelibrary.wiley.com/cochranelibrary/search/ |
| NHS Economic Evaluation Database (NHS EED) | Cochrane Library; 1993 - 13th March 2018 | http://onlinelibrary.wiley.com/cochranelibrary/search/ |
| **Unpublished and ongoing studies** | | |
| ClinicalTrials.gov register | From inception - 16th March 2018 | https://clinicaltrials.gov/ |
| Current controlled trials metaRegister of Controlled Trials (mRCT) – active and archived registers | From inception - 16th March 2018 | https://www.controlled-trials.com/mrct/ |
| International prospective register of systematic reviews (PROSPERO) | From inception - 16th March 2018 | https://www.crd.york.ac.uk/prospero/#searchadvanced |
