# Supplementary material for: Long-term effects of functional appliances in treated versus untreated patients with Class II malocclusion: A systematic review and meta-analysis
Source: PLoS One. 2019 Sep 6;14(9):e0221624. doi: 10.1371/journal.pone.0221624 (PMC6730901; doi:10.1371/journal.pone.0221624)
Supplement: S3 Table — (PDF) [file pone.0221624.s003.pdf]

S3 Table. Search strategy and corresponding results for all databases.

Search	Query	Hits
MEDLINE		
#1	"Class II malocclusion" OR "Class II" OR Class II div* OR Class/*	68634
#2	"prominent upper front teeth" OR "prominent upper teeth" OR "prominent teeth"	107
#3	"increased overjet"	173
#4	Malocclusion, Angle Class II [Mesh]	5724
#5	#1 OR #2 OR #3 OR #4	69184
#6	Functional OR orthopedic OR orthopaedic OR interceptive OR preventive OR bite jump* OR (mandib* AND (advanc* OR enhanc* OR postur* OR protract* OR reposition*)) OR Activator OR Andresen OR Bass OR Bionator OR Bimler OR Frankel OR Fraenkel OR "Functional magnetic system" OR Harvold OR Monoblock OR "Twin block" OR Herbst OR "Mandibular anterior repositioning appliance" OR MARA OR "Eureka spring" OR Forsus OR "Jasper jumper" OR "Sabbagh spring" OR "Twin force"	1749977
#7	appliance OR device	1475369
#8	#6 AND #7	140521
#9	Mandibular advancement [Mesh] OR Orthodontic appliances [Mesh] OR Orthodontics [Mesh]	49535
#10	#8 OR #9	185661
#11	"End of growth" OR "completion of growth" OR long term OR longterm OR follow up OR post retention OR stability OR longitudinal	2251067
#12	#5 AND #10 AND #11	1623

Search	Query	Hits
EMBASE		
#1	("Class II malocclusion" or "Class II" or Class II div*).af.	65204
#2	("prominent upper front teeth" or "prominent upper teeth" or "prominent teeth").af.	11
#3	"increased overjet".af.	145
#4	Malocclusion, Angle Class II/	18774
#5	#1 OR #2 OR #3 OR #4	81885
#6	(Functional or orthopedic or orthopaedic or interceptive or preventive or bite jump* or (mandib* and (advanc* or enhanc* or postur* or protract* or reposition*)) or Activator or Andresen or Bass or Bionator or Bimler or Frankel or Fraenkel or "Functional magnetic system" or Harvold or Monoblock or "Twin block" or Herbst or "Mandibular anterior repositioning appliance" or MARA or "Eureka spring" or Forsus or "Jasper jumper" or "Sabbagh spring" or "Twin force").af.	2223341
#7	(appliance or device).af.	488726
#8	#6 AND #7	54578
#9	Mandibular advancement/	235
#10	Orthodontic appliances/	17679
#11	Orthodontics/	33049
#12	#8 OR #9 OR #10 OR #11	93445
#13	("End of growth" or "completion of growth" or long term or longterm or follow up or post retention or stability or longitudinal).af.	3051080
#14	#5 AND #12 AND #13	1613

Search	Query	Hits
Cochrane Library		
#1	"Class II malocclusion" OR "Class II"	3116
#2	"prominent upper front teeth" OR "prominent upper teeth" OR "prominent teeth"	6
#3	"increased overjet"	10
#4	Class II malocclusion explode all trees	11
#5	#1 OR #2 OR #3 OR #4	3121

S3 Table (continued). Search strategy and corresponding results for all databases.

Search	Query	Hits
Cochrane Library		
#6	Functional OR orthopedic OR orthopaedic OR interceptive OR preventive OR bite jump* OR (mandib* AND (advanc* OR enhanc* OR postur* OR protract* OR reposition*)) OR Activator OR Andresen OR Bass OR Bionator OR Bimler OR Frankel OR Fraenkel OR "Functional magnetic system" OR Harvold OR Monoblock OR "Twin block" OR Herbst OR "Mandibular anterior repositioning appliance" OR MARA OR "Eureka spring" OR Forsus OR "Jasper jumper" OR "Sabbagh spring" OR "Twin force"	84043
#7	appliance OR device	29577
#8	#6 AND #7	4554
#9	Mandibular advancement explode all trees or Orthodontic appliances explode all trees or Orthodontics explode all trees	34
#10	#8 OR #9	4565
#11	"End of growth" OR "completion of growth" OR long term OR longterm OR follow up OR post retention OR stability OR longitudinal	242883
#12	#5 AND #10 AND #11	144
Cochrane Central Register of Controlled Trials (CENTRAL)		76
Cochrane Database of Systematic Reviews (CDSR)		63
Database of Abstracts of Reviews of Effects (DARE)		3
Health Technology Assessment Database (HTA Database)		1
NHS Economic Evaluation Database (NHS EED)		1

Search	Query	Hits
Latin America and the Caribbean (LILACS)		
	(tw:(("Class II malocclusion" OR "Class II" OR class ii div* OR class/*) OR ("prominent upper front teeth" OR "prominent upper teeth" OR "prominent teeth") OR "increased overjet")) AND (tw:((functional OR orthopedic OR orthopaedic OR interceptive OR preventive OR bite jump* OR (mandib* AND (advanc* OR enhanc* OR postur* OR protract* OR reposition*)) OR activator OR andresen OR bass OR bionator OR bimler OR frankel OR fraenkel OR "Functional magnetic system" OR harvold OR monoblock OR "Twin block" OR herbst OR "Mandibular anterior repositioning appliance" OR mara OR "Eureka spring" OR forsus OR "Jasper jumper" OR "Sabbagh spring" OR "Twin force") AND (appliance OR device))) AND (tw:("End of growth" OR "completion of growth" OR long term OR follow up OR post retention OR stability)) AND (instance:"regional") AND (db:("LILACS")) AND (instance:"regional") AND (db:("LILACS") AND jd:("ORTODONTIA" OR "ODONTOLOGIA" OR "MEDICINA"))	42

Search	Query	Hits
Google Scholar		
	"Class II malocclusion" AND "functional appliances" AND "long term"	1510

Search	Query	Hits
Turning Research into Practice (TRIP) database		
	"Class II malocclusion" AND "functional appliances" AND "long term"	14

Search	Query	Hits
Science Citation Index / Science Citation Index Expanded		
#1	TS=("Class II malocclusion" OR "Class II" OR Class II div*)	59008
#2	TS=("prominent upper front teeth" OR "prominent upper teeth" OR "prominent teeth")	15
#3	TS=("increased overjet")	104
#4	#1 OR #2 OR #3	59092

S3 Table (continued). Search strategy and corresponding results for all databases.

Search	Query	Hits
Science Citation Index / Science Citation Index Expanded		
#5	TS=(Functional OR orthopedic OR orthopaedic OR interceptive OR preventive OR bite jump* OR (mandib* AND (advanc* OR enhanc* OR postur* OR protract* OR reposition*)) OR Activator OR Andresen OR Bass OR Bionator OR Bimler OR Frankel OR Fraenkel OR "Functional magnetic system" OR Harvold OR Monoblock OR "Twin block" OR Herbst OR "Mandibular anterior repositioning appliance" OR MARA OR "Eureka spring" OR Forsus OR "Jasper jumper" OR "Sabbagh spring" OR "Twin force")	1725015
#6	TS=(appliance OR device)	815498
#7	#5 AND #6	42131
#8	TS=("End of growth" OR "completion of growth" OR long term OR longterm OR follow up OR post retention OR stability OR longitudinal)	3041564
#9	#4 AND #7 AND #8	296
	For all queries: Indexes=SCI-EXPANDED Timespan=All years	

Search	Query	Hits
Scopus		
	ALL ("Class II malocclusion" AND "functional appliances" AND "long term")	512

Search	Query	Hits
ProQuest Dissertation & Theses Global		
	"Class II malocclusion" AND "functional appliances" AND "long term"	112

Search	Query	Hits
ProQuest Dissertations and Theses — UK & Ireland / Index to Theses		
	"Class II malocclusion" AND "functional appliances" AND "long term"	0

Search	Query	Hits
OpenGrey - formerly System for Information on Grey Literature (SIGLE)		
	"Class II malocclusion" AND "functional appliances" AND "long term"	0

Search	Query	Hits
British Library Direct		
	"Class II malocclusion" AND "functional appliances" AND "long term"	9

Search	Query	Hits
Current Contents Connect - Clinical Medicine		
#1	TS=("Class II malocclusion" OR "Class II" OR Class II div*)	12168
#2	TS=("prominent upper front teeth" OR "prominent upper teeth" OR "prominent teeth")	4
#3	TS=("increased overjet")	68
#4	#1 OR #2 OR #3	12215
#5	TS=(Functional OR orthopedic OR orthopaedic OR interceptive OR preventive OR bite jump* OR (mandib* AND (advanc* OR enhanc* OR postur* OR protract* OR reposition*)) OR Activator OR Andresen OR Bass OR Bionator OR Bimler OR Frankel OR Fraenkel OR "Functional magnetic system" OR Harvold OR Monoblock OR "Twin block" OR Herbst OR "Mandibular anterior repositioning appliance" OR MARA OR "Eureka spring" OR Forsus OR "Jasper jumper" OR "Sabbagh spring" OR "Twin force")	321347
#6	TS=(appliance OR device)	115720
#7	#5 AND #6	10317

S3 Table (continued). Search strategy and corresponding results for all databases.

Search	Query	Hits
Current Contents Connect - Clinical Medicine		
#8	TS=("End of growth" OR "completion of growth" OR long term OR longterm OR follow up OR post retention OR stability OR longitudinal)	837413
#9	#4 AND #7 AND #8	222
	For all queries: Indexes=CM<br>Timespan=All years	

Search	Query	Hits
Scientific Electronic Library Online (SciELO) Citation Index		
#1	TS=("Class II malocclusion" OR "Class II" OR Class II div*)	759
#2	TS=("prominent upper front teeth" OR "prominent upper teeth" OR "prominent teeth")	0
#3	TS=("increased overjet")	17
#4	#1 OR #2 OR #3	771
#5	TS=(Functional OR orthopedic OR orthopaedic OR interceptive OR preventive OR bite jump* OR (mandib* AND (advanc* OR enhanc* OR postur* OR protract* OR reposition*)) OR Activator OR Andresen OR Bass OR Bionator OR Bimler OR Frankel OR Fraenkel OR "Functional magnetic system" OR Harvold OR Monoblock OR "Twin block" OR Herbst OR "Mandibular anterior repositioning appliance" OR MARA OR "Eureka spring" OR Forsus OR "Jasper jumper" OR "Sabbagh spring" OR "Twin force")	19400
#6	TS=(appliance OR device)	7107
#7	#5 AND #6	520
#8	TS=("End of growth" OR "completion of growth" OR long term OR longterm OR follow up OR post retention OR stability OR longitudinal)	35213
#9	#4 AND #7 AND #8	14
	For all queries: Indexes=SCIELO<br>Timespan=All years	

Search	Query	Hits
BIOSIS Citation Index		
#1	TS=("Class II malocclusion" OR "Class II" OR Class II div*)	55062
#2	TS=("prominent upper front teeth" OR "prominent upper teeth" OR "prominent teeth")	17
#3	TS=("increased overjet")	11
#4	#1 OR #2 OR #3	55088
#5	TS=(Functional OR orthopedic OR orthopaedic OR interceptive OR preventive OR bite jump* OR (mandib* AND (advanc* OR enhanc* OR postur* OR protract* OR reposition*)) OR Activator OR Andresen OR Bass OR Bionator OR Bimler OR Frankel OR Fraenkel OR "Functional magnetic system" OR Harvold OR Monoblock OR "Twin block" OR Herbst OR "Mandibular anterior repositioning appliance" OR MARA OR "Eureka spring" OR Forsus OR "Jasper jumper" OR "Sabbagh spring" OR "Twin force")	1162622
#6	TS=(appliance OR device)	681259
#7	#5 AND #6	24326
#8	TS=("End of growth" OR "completion of growth" OR long term OR longterm OR follow up OR post retention OR stability OR longitudinal)	1487148
#9	#4 AND #7 AND #8	53
	For all queries: Indexes=BCI<br>Timespan=All years	

Search	Query	Hits
Conference Proceedings Citation Index - Science		
#1	TS=("Class II malocclusion" OR "Class II" OR Class II div*)	4205
#2	TS=("prominent upper front teeth" OR "prominent upper teeth" OR "prominent teeth")	0
#3	TS=("increased overjet")	2
#4	#1 OR #2 OR #3	4207

S3 Table (continued). Search strategy and corresponding results for all databases.

Search	Query	Hits
Conference Proceedings Citation Index - Science		
#5	TS=(Functional OR orthopedic OR orthopaedic OR interceptive OR preventive OR bite jump* OR (mandib* AND (advanc* OR enhanc* OR postur* OR protract* OR reposition*)) OR Activator OR Andresen OR Bass OR Bionator OR Bimler OR Frankel OR Fraenkel OR "Functional magnetic system" OR Harvold OR Monoblock OR "Twin block" OR Herbst OR "Mandibular anterior repositioning appliance" OR MARA OR "Eureka spring" OR Forsus OR "Jasper jumper" OR "Sabbagh spring" OR "Twin force")	197814
#6	TS=(appliance OR device)	412194
#7	#5 AND #6	10918
#8	TS=("End of growth" OR "completion of growth" OR long term OR longterm OR follow up OR post retention OR stability OR longitudinal)	498647
#9	#4 AND #7 AND #8	10
	For all queries: Indexes=CPCI-S<br>Timespan=All years	

Search	Query	Hits
ISI Proceedings		
	"Class II malocclusion" AND "functional appliances" AND "long term"	10

Search	Query	Hits
ClinicalTrials.gov register		
	Condition: Class II malocclusion; Intervention: functional appliances	14

Search	Query	Hits
Current controlled trials metaRegister of Controlled Trials (mRCT) – active and archived registers		
	"Class II malocclusion" AND "functional appliances"	2

Search	Query	Hits
International prospective register of systematic reviews (PROSPERO)		
	Class II malocclusion AND functional appliances	17
