# Supplementary material for: Long-term effects of functional appliances in treated versus untreated patients with Class II malocclusion: A systematic review and meta-analysis
Source: PLoS One. 2019 Sep 6;14(9):e0221624. doi: 10.1371/journal.pone.0221624 (PMC6730901; doi:10.1371/journal.pone.0221624)
Supplement: S4 Table — (PDF) [file pone.0221624.s004.pdf]

S4 Table. Studies excluded with corresponding main reason of exclusion.

Study	Reference	Reason for exlusion
AAO COSA 2005	[1]	Other study design
Al-Jewair 2012	[2]	Study on the short-term effects
Al-Jewair 2013	[3]	Study on the short-term effects
Alió-Sanz 2012	[4]	Study on the short-term effects
Angelieri 2009	[5]	Patients not compared to untreated subjects
Baccetti 2010	[6]	Study on the short-term effects
Ball 1991	[7]	Study on the short-term effects
Barnett 2007	[8]	Study on the short-term effects
Bavbek 2016	[9]	Study on the short-term effects
Berg 1979	[10]	Treated patients not compared to any controls
Berg 1983	[11]	Orthodontic, but not functional appliances
Bigliazzi 2015	[12]	Outcomes not measured on lateral cephalograms
Bolmgren 1986	[13]	Study on the short-term effects
Bredy 1987	[14]	Not available abstract
Byloff-Clar 1970	[15]	Not available abstract
Cacciatore 2014	[16]	Study on the short-term effects
Casellas 2001	[17]	Study on the short-term effects
Chen 2011	[18]	Other outcomes were measured
Chhibber 2010	[19]	Other study design
Cozza 2003	[20]	Other study design
Craig 1977	[21]	Study on the short-term effects
Criswell 2011	[22]	Study on the short-term effects
Dalci 2014	[23]	Study on the short-term effects
DeVincenzo 1991	[24]	Patients not compared to untreated subjects
Dolce 2005	[25]	Outcomes not measured on lateral cephalograms
Dolce 2007	[26]	Patients not compared to untreated subjects
Dos Santos-Pinto 2013	[27]	Study on the short-term effects
Drage 1990	[28]	Study on the short-term effects
Ehmer 1990	[29]	Treated patients not compared to any controls
Falck 1983	[30]	Not available abstract
Faxén Sepanian 2014	[31]	Other outcomes were measured
Filip 1970	[32]	Not available abstract
Flores-Mir 2009	[33]	Study on the short-term effects
Foncatti 2017	[34]	Patients not compared to untreated subjects
Franchi 1999	[35]	Study on the short-term effects
Franchi 2006	[36]	Study on the short-term effects
Franchi 2011	[37]	Orthodontic, but not functional appliances
Franchi 2016	[38]	Outcomes not measured on lateral cephalograms
Frankel 1983	[39]	Other outcomes were measured
Fry 2006	[40]	Patients not compared to untreated subjects
Ghislanzoni 2011	[41]	Study on the short-term effects
Han 2014	[42]	Patients not compared to untreated subjects
Hansen 1992	[43]	Patients not compared to untreated subjects
Humphrey 2016	[44]	Study on the short-term effects
Jacob 2014	[45]	Functional appliances associated with headgear
Jakobsone 2013	[46]	Study on the short-term effects
Janson 2007	[47]	Patients not compared to untreated subjects
Johannesen 1972	[48]	Not available abstract
Karlowska 1971	[49]	Not available abstract
Keeling 1998	[50]	Study on the short-term effects
Keski-Nisula 2008	[51]	Study on the short-term effects
Knight 1988	[52]	Study on the short-term effects
Koroluk 2003	[53]	Other outcomes were measured
Lall 2011	[54]	Study on the short-term effects
Lima 2013	[55]	Study on the short-term effects
Livieratos 1995	[56]	Patients not compared to untreated subjects
Luder 1982	[57]	Study on the short-term effects
Lux 2001	[58]	Study on the short-term effects

S4 Table (continued). Studies excluded with corresponding main reason of exclusion.

Study		Reason for exlusion
Madone 1984a	[59]	Treated patients not compared to any controls
Madone 1984b	[60]	Not available abstract
Mills 2000	[61]	Study on the short-term effects
Mongini 1987	[62]	Orthodontic, but not functional appliances
Morris 1998	[63]	Study on the short-term effects
Morteson 2004	[64]	Participants aged more than16 years
Nelson 2007	[65]	Patients not compared to untreated subjects
O'Brien 2009	[66]	Study on the short-term effects
Omblus 1997	[67]	Patients not compared to untreated subjects
Pancherz 1977	[68]	Patients not compared to untreated subjects
Pancherz 1986	[69]	Treated patients not compared to any controls
Pancherz 1989	[70]	Not available full-text
Pancherz 1993	[71]	Patients not compared to untreated subjects
Pancherz 1994	[72]	Patients not compared to untreated subjects
Pancherz 1998	[73]	Patients not compared to untreated subjects
Pancherz 2003	[74]	Other outcomes were measured
Pancherz 2015	[75]	Treated patients not compared to any controls
Pancherz 2015	[76]	Treated patients not compared to any controls
Pangrazio 2012	[77]	Study on the short-term effects
Pavoni 2017	[78]	Other outcomes were measured
Perillo 1996	[79]	Patients not compared to untreated subjects
Perillo 2011	[80]	Treated patients not compared to any controls
Phelan 2012	[81]	Study on the short-term effects
Righellis 1983	[82]	Study on the short-term effects
Sander 1995	[83]	Study on the short-term effects
Sawrie 2007	[84]	Patients not compared to untreated subjects
Scalzone 2015	[85]	Study on the short-term effects
Schadlbauer 1984	[86]	Not available abstract
Schütz-Fransson 2006	[87]	Orthodontic, but not functional appliances
Siara-Olds 2010	[88]	Study on the short-term effects
Sivakumar 2005	[89]	Other study design
Stuber 1990	[90]	Not available full-text
Stuber 1990	[91]	Study on the short-term effects
Thompson 2001	[92]	Participants aged more than16 years
Tomblyn 2015	[93]	Study on the short-term effects
Tomblyn 2016	[94]	Study on the short-term effects
Tulloch 1998	[95]	Study on the short-term effects
Ulusoy 2014	[96]	Study on the short-term effects
Valant 1983	[97]	Study on the short-term effects
VanLaecken 2006	[98]	Study on the short-term effects
Vardimon 2001	[99]	Study on the short-term effects
Voudouris 2003	[100]	Animal study
Voudouris 2003	[101]	Animal study
Weschler 2005	[102]	Patients not compared to untreated subjects
Wheeler 2002	[103]	Study on the short-term effects
Wortham 2009	[104]	Other outcomes were measured
Yassaei 2012	[105]	Treated patients not compared to any controls
Yassaei 2014	[106]	Treated patients not compared to any controls
Yüksel 2010	[107]	Treated patients not compared to any controls
Zelderloo 2017	[108]	Patients not compared to untreated subjects
