# Supplementary material for: Long-term effects of functional appliances in treated versus untreated patients with Class II malocclusion: A systematic review and meta-analysis
Source: PLoS One. 2019 Sep 6;14(9):e0221624. doi: 10.1371/journal.pone.0221624 (PMC6730901; doi:10.1371/journal.pone.0221624)
Supplement: S5 Table — (PDF) [file pone.0221624.s005.pdf]

S5 Table. Results during the overall observational period for each outcome included in the meta-analysis.

Study	Intervention	Results								
		Treated			Control			Diff	95% CI	P
		Mean	SD	N	Mean	SD	N			
**SNA (degrees)**										
Pavoni 2017 (early)	Bio / Act + MBA	-0.2	2.0	23	0.6	1.6	16	-0.8	-2.0, 0.4	0.177
Pavoni 2017 (late)	Bio / Act + MBA	-1.3	2.7	23	-0.6	2.0	15	-0.7	-2.4, 1.0	0.391
Freeman 2009	Fr2	0.0	1.7	30	0.7	2.1	20	-0.7	-	-
Angelieri 2014	Fr2	0.2	1.4	17	0.9	1.7	17	-0.7	-	-
Wigal 2008 (males)	Hb + MBA	0.1	2.4	7	1.6	1.6	7	-1.5	-	0.172
Wigal 2008 (females)	Hb + MBA	-1.3	3.1	15	1.9	2.6	15	-3.2	-	0.005
Drosen 2018 (males)	Hb +/- MBA	0.1	1.7	13	0.7	1.7	13	-0.6	-	0.297
Alhoraibi 2017 (early)	FRD + MBA	-1.7	0.6	18	0.5	0.4	18	-2.2	-	0.000
Alhoraibi 2017 (late)	FRD + MBA	1.1	1.8	21	0.7	0.3	21	0.4	-	0.000
**A to N perp (mm)**										
Freeman 2009	Fr2	-1.6	1.4	30	0.1	1.5	20	-1.7	-	-
Angelieri 2014	Fr2	1.6	1.4	17	0.9	1.8	17	0.7	-	-
Alhoraibi 2017 (early)	FRD + MBA	-1.2	0.9	18	0.6	0.4	18	-1.8	-	0.000
Alhoraibi 2017 (late)	FRD + MBA	-6.3	1.7	21	0.5	0.4	21	-6.8	-	0.000
**Co-A (mm)**										
Freeman 2009	Fr2	10.2	3.4	30	10.4	3.7	20	-0.2	-	-
Angelieri 2014	Fr2	6.2	2.6	17	5.7	2.9	17	0.5	-	-
Wigal 2008 (males)	Hb + MBA	6.4	4.4	7	8.8	3.9	7	-2.4	-	-
Wigal 2008 (females)	Hb + MBA	4.0	3.2	15	8.5	2.9	15	-4.5	-	-
Alhoraibi 2017 (early)	FRD + MBA	0.9	1.1	18	2.4	0.7	18	-1.5	-	0.000
Alhoraibi 2017 (late)	FRD + MBA	1.3	1.3	21	0.6	0.6	21	0.7	-	0.000
**SNB (degrees)**										
Pavoni 2017 (early)	Bio / Act + MBA	1.4	2.0	23	2.2	1.6	16	-0.8	-2.0, 0.4	0.209
Pavoni 2017 (late)	Bio / Act + MBA	2.1	2.1	23	1.0	1.9	15	1.1	-0.2, 2.5	0.105
Freeman 2009	Fr2	3.5	1.7	30	1.8	2.1	20	1.7	-	-
Angelieri 2014	Fr2	2.3	1.9	17	1.7	1.8	17	0.6	-	-
Wigal 2008 (males)	Hb + MBA	2.1	2.2	7	1.4	1.8	7	0.7	-	0.525
Wigal 2008 (females)	Hb + MBA	1.0	2.4	15	2.2	2.1	15	-1.2	-	0.165
Drosen 2018 (males)	Hb +/- MBA	1.7	1.7	13	2.0	1.9	13	-0.3	-	0.878
Alhoraibi 2017 (early)	FRD + MBA	2.5	1.3	18	3.3	1.2	18	-0.8	-	0.050
Alhoraibi 2017 (late)	FRD + MBA	2.5	1.2	21	2.2	0.6	21	0.3	-	0.200
**Pg to N perp (mm)**										
Pavoni 2017 (early)	Bio / Act + MBA	4.3	3.8	23	3.4	4.1	16	0.9	-1.7, 3.5	0.479
Pavoni 2017 (late)	Bio / Act + MBA	6.8	2.3	23	3.7	3.4	15	3.1	1.3, 5.0	0.001
Freeman 2009	Fr2	2.5	3.6	30	3.0	3.1	20	-0.5	-	-
Angelieri 2014	Fr2	6.6	4.0	17	3.6	2.8	17	3.0	-	-
Alhoraibi 2017 (early)	FRD + MBA	0.4	0.9	18	1.6	2.3	18	-1.2	-	0.573
Alhoraibi 2017 (late)	FRD + MBA	1.9	0.9	21	1.0	1.6	21	0.9	-	0.570
**Co-Gn (mm)**										
Pavoni 2017 (early)	Bio / Act + MBA	16.8	4.6	23	17.5	4.7	16	-0.7	-3.8, 2.3	0.632
Pavoni 2017 (late)	Bio / Act + MBA	20.5	3.7	23	15.0	2.5	15	5.5	3.3, 7.7	0.000
Freeman 2009	Fr2	20.6	4.9	30	17.6	4.5	20	3.0	-	-
Angelieri 2014	Fr2	15.2	4.3	17	11.5	4.3	17	3.7	-	-
Wigal 2008 (males)	Hb + MBA	15.4	4.5	7	14.1	3.0	7	1.3	-	-
Wigal 2008 (females)	Hb + MBA	9.8	2.3	15	13.3	3.0	15	-3.5	-	-
Alhoraibi 2017 (early)	FRD + MBA	4.0	2.4	18	0.1	0.7	18	3.9	-	0.200
Alhoraibi 2017 (late)	FRD + MBA	1.7	2.4	21	0.0	0.7	21	1.7	-	0.772
**ANB (degrees)**										
Wieslander 1979	Act	-2.3	1.4	23	-0.5	1.0	23	-1.8	-	-
Pavoni 2017 (early)	Bio / Act + MBA	-1.6	1.2	23	-1.6	1.1	16	0.0	-0.8, 0.7	0.859
Pavoni 2017 (late)	Bio / Act + MBA	-3.4	1.5	23	-1.6	1.4	15	-1.8	-2.8, -0.8	0.001
Freeman 2009	Fr2	-3.5	1.2	30	-1.3	1.7	20	-2.2	-	-
Angelieri 2014	Fr2	-2.1	1.3	17	-0.8	1.9	17	-1.3	-	-
Wigal 2008 (males)	Hb + MBA	-2.1	1.6	7	0.2	1.2	7	-2.3	-	0.010
Wigal 2008 (females)	Hb + MBA	-2.3	1.7	15	-0.3	1.4	15	-2.0	-	0.002
Drosen 2018 (males)	Hb +/- MBA	-1.7	1.2	13	-1.3	1.2	13	-0.4	-	0.358

S5 Table (continued). Results during the overall observational period for each outcome included in the meta-analysis.

Study	Intervention	Results								
		Treated			Control			Diff	95% CI	P
		Mean	SD	N	Mean	SD	N			
Alhoraibi 2017 (early)	FRD + MBA	-3.3	1.6	18	-2.9	1.3	18	-0.4	-	0.537
Alhoraibi 2017 (late)	FRD + MBA	-0.9	1.3	21	-1.5	0.6	21	0.6	-	0.000
**Wits (mm)**										
Pavoni 2017 (early)	Bio / Act + MBA	-0.5	2.9	23	1.2	3.5	16	-1.7	-3.8, 0.3	0.098
Pavoni 2017 (late)	Bio / Act + MBA	-4.0	3.9	23	1.8	3.7	15	-5.8	-8.3, -3.2	0.000
Freeman 2009	Fr2	-3.1	2.4	30	1.8	2.5	20	-4.9	-	-
Angelieri 2014	Fr2	-2.2	2.2	17	0.4	2.2	17	-2.6	-	-
Wigal 2008 (males)	Hb + MBA	-2.2	2.1	7	0.1	2.2	7	-2.3	-	0.073
Wigal 2008 (females)	Hb + MBA	-1.3	1.9	15	-0.1	1.6	15	-1.2	-	0.072
Drosen 2018 (males)	Hb +/- MBA	-1.6	2.5	13	0.8	1.9	13	-2.4	-	0.006
Alhoraibi 2017 (early)	FRD + MBA	-2.9	1.3	18	0.8	0.9	18	-3.7	-	0.000
Alhoraibi 2017 (late)	FRD + MBA	-2.3	1.7	21	0.7	1.2	21	-3.0	-	0.000
**Co-Gn/Co-A diff (mm)**										
Freeman 2009	Fr2	10.4	2.6	30	7.8	2.8	20	2.6	-	-
Angelieri 2014	Fr2	8.9	3.1	17	5.6	3.1	17	3.3	-	-
Wigal 2008 (males)	Hb + MBA	7.7	1.8	7	5.4	2.8	7	2.3	-	0.090
Wigal 2008 (females)	Hb + MBA	5.8	4.0	15	4.8	2.5	15	1.0	-	0.404
Alhoraibi 2017 (early)	FRD + MBA	3.0	2.3	18	-2.3	1.1	18	5.3	-	0.000
Alhoraibi 2017 (late)	FRD + MBA	0.4	3.2	21	-0.6	0.8	21	1.0	-	0.004

Act, Activator; Bio, Bionator; Fr2, Frankel-2; Hb, Herbst; FRD, Forsus; MBA, multi-bracket appliances;

Mx skeletal, maxillary skeletal outcomes; SNA, SNA angle; A to N perp, A point to N perpendicular distance; Co-A, Co-A distance;

Md skeletal, mandibular skeletal outcomes; SNB, SNB angle; Pg to N perp, Pg point to N perpendicular distance; Co-Gn, Co-Gn distance;

Mx-md skeletal, maxillo-mandibular outcomes; ANB, ANB angle; Wits, Wits appraisal; Co-Gn/Co-A diff, Co-Gn/Co-A difference;

SD, standard deviation; N, number of participants;

Diff, difference; 95% CI, 95% confidence intervals; P, P value reported by the original study.
