# Supplementary material for: Long-term effects of functional appliances in treated versus untreated patients with Class II malocclusion: A systematic review and meta-analysis
Source: PLoS One. 2019 Sep 6;14(9):e0221624. doi: 10.1371/journal.pone.0221624 (PMC6730901; doi:10.1371/journal.pone.0221624)
Supplement: S6 Table — (PDF) [file pone.0221624.s006.pdf]

S6 Table. Results during the overall observational period for each outcome excluded by the meta-analysis.

Study	Outcome (mm)	Results								
		Treated			Control			Diff	95% CI	P
		Mean	SD	N	Mean	SD	N			
Wieslander 1979	A to S perp	2.8	2.7	23	4.1	2.1	23	-1.3	-	-
Falck 1991 (males)	Horiz. A to ORS	10.2	2.3	19	10.2	3.2	18	0.0	-	-
Falck 1991 (females)	Horiz. A to ORS	6.2	2.0	31	7.2	2.9	20	-0.9	-	-
Wigal 2008 (males)	Olp-A	6.2	2.2	7	8.1	2.4	7	-1.9	-	0.139
Wigal 2008 (females)	Olp-A	3.5	2.3	15	6.6	2.1	15	-3.1	-	0.001
Wieslander 1979	Pg to S perp	6.3	4.4	23	5.5	3.4	23	0.8	-	-
Falck 1991 (males)	Horiz. Pg to ORS	19.6	4.7	19	15.1	4.3	18	4.5	-	-
Falck 1991 (females)	Horiz. Pg to ORS	13.3	3.9	31	9.2	5.6	20	4.0	-	-
Falck 1991 (males)	Horiz. B to ORS	16.9	4.5	19	12.1	3.7	18	4.7	-	-
Falck 1991 (females)	Horiz. B to ORS	11.7	3.0	31	7.4	4.1	20	4.3	-	-
Wieslander 1979	Ar-Gn	11.8	5.1	23	11.2	4.5	23	0.7	-	-
Wieslander 1979	Co-Gn	13.2	5.7	23	11.8	5.7	23	1.5	-	-
Falck 1991 (males)	Co-Gn	23.2	3.7	19	20.3	3.8	18	2.9	-	-
Falck 1991 (females)	Co-Gn	18.1	3.1	31	14.5	3.6	20	3.6	-	-
Wigal 2008 (males)	Olp-Co	1.4	3.3	7	0.6	3.2	7	0.8	-	0.665
Wigal 2008 (females)	Olp-Co	0.5	2.1	15	1.8	1.8	15	-1.3	-	0.077
Wigal 2008 (males)	Olp-Pg	10.9	2.7	7	9.2	4.3	7	1.7	-	0.398
Wigal 2008 (females)	Olp-Pg	6.6	3.5	15	9.7	1.9	15	-3.1	-	0.005
Wieslander 1979	Co to mand	8.7	4.1	23	9.4	4.6	23	-0.6	-	-
Pavoni 2017 (early)	Co-Go	10.9	4.6	23	11.8	2.7	16	-0.9	-3.5, 1.7	0.482
Pavoni 2017 (late)	Co-Go	14.0	3.7	23	11.6	3.0	15	2.4	0.2, 4.8	0.036
Drosen 2018 (males)	Ar-Go	11.7	3.0	13	9.4	2.1	13	2.3	-	0.029

Mx skeletal, maxillary skeletal outcomes; A to S perp, A point to S perpendicular distance; Horiz. A to ORS, horizontal distance of A point to occipital reference system; Olp-A, distance of A point to occlusal line perpendicular;

Md skeletal, mandibular skeletal outcomes; Pg to S perp, Pg point to S perpendicular distance; Horiz. B or Pg to ORS, horizontal distance of B point or Pg point to occipital reference system; Ar-Gn, Ar-Gn distance; Olp-Co, distance of Co point to occlusal line perpendicular; Olp-Pg, distance of Pg point to occlusal line perpendicular; Co to mand, distance of Co point to mandibular plane; Co-Go, Co-Go distance; Ar-Go, Ar-Go distance;

Mx-md skeletal, maxillo-mandibular outcomes; ANB, ANB angle; Wits, Wits appraisal; Co-Gn/Co-A diff, Co-Gn/Co-A difference;

SD, standard deviation; N, number of participants;

Diff, difference; 95% CI, 95% confidence intervals; P, P value reported by the original study.
