# Supplementary material for: Long-term effects of functional appliances in treated versus untreated patients with Class II malocclusion: A systematic review and meta-analysis
Source: PLoS One. 2019 Sep 6;14(9):e0221624. doi: 10.1371/journal.pone.0221624 (PMC6730901; doi:10.1371/journal.pone.0221624)
Supplement: S1 Appendix — (PDF) [file pone.0221624.s007.pdf]

S1 Appendix. Eligibility criteria with rationale.

Study designs

The following study designs were included: randomised controlled trials (RCTs), controlled (non-randomised) clinical trials (CCTs), controlled before-after (CBA) studies, and case-control or nested case-control studies. Prospective and retrospective cohort studies, cross-sectional studies, case series, and case reports were excluded.

Since the aim of this review was to compare Class II malocclusion patients treated with functional appliances to untreated subjects, only experimental and observational studies with a comparison group were included [1]. The decision to evaluate both randomised and non-randomised controlled trials was made, in order to collect a wide range of studies. A limited number of trials assessing any type of outcome in the long-term was found in previous systematic reviews [2-5].

Participants

Children and adolescents (aged 16 years or under) receiving orthodontic treatment to correct Class II malocclusion were included. Active treatment with functional appliances had to be completed by the age of 16 years, to allow for a sufficient post-retention period at growth completion. Studies were considered eligible regardless of how the baseline disease was measured (e.g. dental casts, lateral cephalograms) and its severity (e.g. full or half Class II molar relationship, depending on whether the lower molars were placed in a completely or partially posterior position relative to the upper molars, respectively).

Given the potential of functional appliances in modifying the patient growth, they are commonly used in childhood and adolescence [6]. Thus, the analysis of the treatment effects of these appliances on adults was considered to be of minor relevance.

Although there is no agreement on the definitions of childhood and adolescence, in a recent

systematic review of the Cochrane Collaboration ‘children’ were defined as subjects aged from 7 to 11 years, whereas ‘adolescents’ were defined as subjects aged from 12 to 16 years [6]. This practical categorisation is also used in other systematic reviews [7], trials [8], and some national health services (e.g. United Kingdom and Italy). Alternative methods to establish the growth phase, such as the hand-and-wrist maturation method [9] or the cervical vertebral maturation method [10] were not chosen as inclusion criteria, due to them not being globally accepted [11].

Trials including participants with a cleft lip or palate or both, other craniofacial deformity/syndrome (such as Apert, Crouzon, Hemifacial Microsomia/Goldenhar, Moebius, Pierre Robin, Treacher Collins syndromes or craniosynostosis), syndromes affecting the craniofacial structures or patients with temporo-mandibular joint disorders were excluded.

Interventions

Any type of functional appliance, defined as a removable or fixed orthodontic appliance that postures the mandible forward [12]. Functional appliances had to be worn alone or in combination with multi-bracket therapy so as to be included. When functional appliances were worn alone, this therapy could also take place after the functional appliance treatment. A concurrent or subsequent phase with multi-bracket appliances to align teeth is the most common clinical pathway in Orthodontics [2, 13].

Conversely, association with other Class II devices designed primarily to restrain the maxilla (e.g. headgear) was set as an exclusion criterion. Mechanics opposite to those employed during the functional appliance therapy were kept out, so as to reduce co-intervention bias [14].

Only functional appliances worn for 6 months or longer were considered eligible. The duration of treatment with functional appliances is usually from 6 to 18 months, followed by night-time insertion of the appliance, or though the use of a stabilization plate [5, 6, 12, 13, 15, 16]. A wider spectrum of treatment period was considered to be valid, in order to include as many eligible

studies as possible.

Comparators

Class II malocclusion patients treated with functional appliances were compared only to untreated Class II subjects. No other type of orthodontic appliance or brace was considered as a comparator.

Patterns of mandibular growth in subjects with untreated Class II malocclusion differ from those of untreated subjects with normal occlusion (Class I). The deficiency in mandibular growth in Class II subjects is significant at the growth spurt, and it is maintained at the post-pubertal stage. Thus, the use of untreated Class II comparators in studies or reviews on the effectiveness of dentofacial orthopaedics on mandibular growth is recommended [17].

For this comparison, groups had to be of similar ages at the commencement of the observational period (age differences between the treated and untreated groups less than 18 months).

Outcomes

The following clinically important outcomes were recorded:

- Cephalometric skeletal measurements evaluating the antero-posterior position of the maxilla and mandible, the total mandibular length or length of its parts (ramus and corpus), the mutual relationship between the two jaws.
- Soft tissue changes of both lips and chin, measured on lateral cephalograms.

Measurements derived from any cephalometric analysis were included. Due to possible variation in outcome definitions over time, outcomes were collected as reported. Definitions of outcomes as reported in individual studies were extracted as well.

It is not possible to establish the true nature of a malocclusion without information on the underlying skeletal relationships. Cephalometric analysis still remains the most widespread, safest

and most precise method of measuring changes to skeletal structures [18]. The use of alternative methods, such as the cone-beam computer tomography (CBCT), should not be implemented for this purpose [19]. According to the ‘Guidelines on CBCT for dental and maxillofacial radiology’, large volume CBCT should not be used as a standard diagnosis method in Orthodontics. In comparison to conventional radiograph, CBCT has higher radiation doses and, having so stated, its use may be justified in treatment planning, solely for complex cases of skeletal abnormality, particularly those requiring combined orthodontic/surgical management [19].

Lateral cephalograms can also be useful for analysing soft-tissue changes. At this time, alternative methods, such as two-dimensional or three-dimensional photographs, are not widespread as much as lateral cephalograms in orthodontic practice and research.

Timing

Studies were selected for inclusion based on the duration of follow-up of outcomes. Studies should have measured outcomes at the end of growth, defined by age or using indicators of the growth phase. Otherwise, studies should have a post-retention period of at least 3 years.

Contrary to the age threshold established when selecting the inclusion of participants, no age criteria was used to define the end of growth. Literature disagrees on the completion of the maxillofacial unit growth [20-24].

Since the real and stable results produced by functional appliances are the areas of interest, a minimum post-retention period after functional jaw orthopaedics was imposed. There is no recognised duration for retainers to be worn after multi-bracket appliances. It has been shown that if patients stop wearing retainers for between 1 and 2 years after correction of teeth positions there is a risk of long-term relapse [25]. There is no definitive agreement on the retention protocol after functional appliance therapy either [15, 26, 27]. Nevertheless, it is clinically unlikely that a treatment initiated in adolescence and skeletally stable after a 3 year follow up could relapse. For

these reasons, a post-retention period of at least 3 years as eligibility criteria was set.

References

1. Grimes DA, Schulz KF. An overview of clinical research: the lay of the land. Lancet. 2002;359:57-61.
2. Perinetti G, Primožič J, Furlani G, Franchi L, Contardo L. Treatment effects of fixed functional appliances alone or in combination with multibracket appliances: A systematic review and meta-analysis. Angle Orthod. 2015;85:480-92.
3. Koretsi V, Zymperdikas VF, Papageorgiou SN, Papadopoulos MA. Treatment effects of removable functional appliances in patients with Class II malocclusion: a systematic review and meta-analysis. Eur J Orthod. 2015;37:418-34.
4. Zymperdikas VF, Koretsi V, Papageorgiou SN, Papadopoulos MA. Treatment effects of fixed functional appliances in patients with Class II malocclusion: a systematic review and meta-analysis. Eur J Orthod. 2016;38:113-26.
5. Nucera R, Lo Giudice A, Rustico L, Matarese G, Papadopoulos MA, Cordasco G. Effectiveness of orthodontic treatment with functional appliances on maxillary growth in the short term: A systematic review and meta-analysis. Am J Orthod Dentofacial Orthop. 2016;149:600-611.e3.
6. Batista KB, Thiruvenkatachari B, Harrison JE, O'Brien KD. Orthodontic treatment for prominent upper front teeth (Class II malocclusion) in children and adolescents. Cochrane Database Syst Rev. 2018;3:CD003452.
7. Sunnak R, Johal A, Fleming PS. Is orthodontics prior to 11 years of age evidence-based? A systematic review and meta-analysis. J Dent. 2015 May;43(5):477-86.
8. Nimri KA1, Richardson A. Applicability of interceptive orthodontics in the community. Br J Orthod. 1997;24(3):223-8.
9. Fishman LS. Radiographic evaluation of skeletal maturation. A clinically oriented method based on hand-wrist films. Angle Orthod. 1982;52:88-112.

10. Franchi L, Baccetti T, McNamara JA Jr. Mandibular growth as related to cervical vertebral maturation and body height. Am J Orthod Dentofacial Orthop. 2000;118:335-40.
11. Santiago RC, de Miranda Costa LF, Vitral RW, Fraga MR, Bolognese AM, Maia LC. Cervical vertebral maturation as a biologic indicator of skeletal maturity. Angle Orthod. 2012;82(6):1123-31.
12. McNamara JA Jr, McNamara L, Graber LW. Optimizing Orthodontic and Dentofacial Orthopedic Treatment Timing. In: Graber TM, Vanarsdall RL, Vig KWL, editors. Orthodontics: current principles and techniques. 5th ed. St. Louis: Elsevier Mosby; 2012. pp. 477-514.
13. Ishaq RA, AlHammadi MS, Fayed MM, El-Ezz AA, Mostafa Y. Fixed functional appliances with multibracket appliances have no skeletal effect on the mandible: A systematic review and meta-analysis. Am J Orthod Dentofacial Orthop. 2016;149:612-24.
14. Sackett DL. Bias in analytic research. J Chronic Dis. 1979;32:51-63.
15. Perinetti G, Primožič J, Franchi L, Contardo L. Treatment Effects of Removable Functional Appliances in Pre-Pubertal and Pubertal Class II Patients: A Systematic Review and Meta-Analysis of Controlled Studies. PLoS One. 2015;10:e0141198.
16. Pacha MM, Fleming PS, Johal A. A comparison of the efficacy of fixed versus removable functional appliances in children with Class II malocclusion: A systematic review. Eur J Orthod. 2016;38:621-630.
17. Stahl F, Baccetti T, Franchi L, McNamara JA Jr. Longitudinal growth changes in untreated subjects with Class II Division 1 malocclusion. Am J Orthod Dentofacial Orthop. 2008;134:125-37.
18. Ackerman JL, Nguyen T, Proffit WR. The decision-making process in orthodontics. In: Graber TM, Vanarsdall RL, Vig KWL, editors. Orthodontics: current principles and techniques. 5th ed. St. Louis: Elsevier Mosby; 2012. pp. 3-58.
19. SEDENTEXCT project. Guidelines on CBCT for Dental and Maxillofacial Radiology. European Commission. 2011. Available from:

http://www.sedentexct.eu/content/guidelines-cbct-dental-and-maxillofacial-radiology.

20. Bjork A. Variations in the growth pattern of the human mandible: longitudinal radiographic study by the implant method. J Dent Res. 1963;42:400-11.
21. Love RJ, Murray JM, Mamandras AH. Facial growth in males 16 to 20 years of age. Am J Orthod Dentofacial Orthop. 1990;97:200-6.
22. Bishara SE, Treder JE, Jakobsen JR. Facial and dental changes in adulthood. Am J Orthod Dentofacial Orthop. 1994;106:175-86.
23. West KS, McNamara JA Jr. Changes in the craniofacial complex from adolescence to midadulthood: a cephalometric study. Am J Orthod Dentofacial Orthop. 1999;115:521-32.
24. Pecora NG, Baccetti T, McNamara JA Jr. The aging craniofacial complex: a longitudinal cephalometric study from late adolescence to late adulthood. Am J Orthod Dentofacial Orthop. 2008;134:496-505.
25. Littlewood SJ, Millett DT, Doubleday B, Bearn DR, Worthington HV. Retention procedures for stabilising tooth position after treatment with orthodontic braces. Cochrane Database Syst Rev. 2016;1:CD002283.
26. Bondemark L, Holm AK, Hansen K, Axelsson S, Mohlin B, Brattstrom V, Paulin G, Pietila T. Long-term stability of orthodontic treatment and patient satisfaction. A systematic review. Angle Orthod. 2007;77:181-91.
27. Bock NC, von Bremen J, Ruf S. Stability of Class II fixed functional appliance therapy--a systematic review and meta-analysis. Eur J Orthod. 2016;38:129-39.
