# Supplementary material for: Long-term effects of functional appliances in treated versus untreated patients with Class II malocclusion: A systematic review and meta-analysis
Source: PLoS One. 2019 Sep 6;14(9):e0221624. doi: 10.1371/journal.pone.0221624 (PMC6730901; doi:10.1371/journal.pone.0221624)
Supplement: S2 Appendix — (PDF) [file pone.0221624.s008.pdf]

S2 Appendix. References to studies excluded from this review.

1. AAO Council on Scientific Affairs (COSA). Functional appliances and long-term effects on mandibular growth. Am J Orthod Dentofacial Orthop. 2005;128(3):271-2.
2. Al-Jewair TS, Preston CB, Moll EM, Dischinger T. A comparison of the MARA and the AdvanSync functional appliances in the treatment of Class II malocclusion. Angle Orthod. 2012;82(5):907-14.
3. Al-Jewair T. Treatment Effects of the Edgewise Mandibular Anterior Repositioning Appliance (MARA) in Patients with Class II Malocclusions: A Cephalometric Study. M.Sc. Thesis, Ann Arbor: State University of New York at Buffalo. 2013.
4. Alió-Sanz JJ, Iglesias-Conde C, Lorenzo-Pernía J, Iglesias-Linares A, Mendoza-Mendoza AC, solano-Reina E. Cranial base and maxillary changes in patients treated with Frankel's functional regulator (1b). Med Oral Patol Oral Cir Bucal. 2012;17(4):e689-e96.
5. Angelieri F, Bommarito S, Kanashiro LK, Sannomyia EK, Andreoli FAM. Estabilidade das alterações tegumentares promovidas pelo aparelho regulador de função Frãnkel 2 (RF-2). Ortodontia. 2009;42(5):363-8.
6. Baccetti T, McNamara JA. The impact of functional jaw orthopedics in subjects with unfavorable class II skeletal patterns. Prog Orthod. 2010;11(2):118-26.
7. Ball JV, Hunt NP. Vertical skeletal change associated with Andresen, Harvold, and Begg treatment. Eur J Orthod. 1991;13(1):47-52.
8. Barnett GA. Skeletal and dentoalveolar effects of Class II treatment using a CrossBow appliance compared to an untreated control group. M.Sc. Thesis, Ann Arbor: State University of Alberta (Canada). 2007.
9. Bavbek NC, Tuncer BB, Turkoz C, Ulusoy C, Tuncer C. Changes in airway dimensions and hyoid bone position following class II correction with forsus fatigue resistant device. Clin Oral Investig. 2016;20(7):1747-55.
10. Berg R. Post-retention analysis of treatment problems and failures in 264 consecutively

treated cases. Eur J Orthod. 1979;1(1):55-68.

11. Berg R. Stability of deep overbite correction. Eur J Orthod. 1983;5(1):75-83.
12. Bigliazzi R, Franchi L, Bertoz AP, McNamara JA, Jr., Faltin K, Jr., Bertoz FA. Morphometric analysis of long-term dentoskeletal effects induced by treatment with Balters bionator. Angle Orthod. 2015;85(5):790-8.
13. Bolmgren GA, Moshiri F. Bionator treatment in Class II, division 1. Angle Orthod. 1986;56(3):255-62.
14. Bredy E, Jungto H. The elastic-open Klammt activator--results of follow-up. Fortschr Kieferorthop. 1987;48(2):87-93.
15. Byloff-Clar H, Droschl H. Results of follow-up studies in closed-bite. Osterr Z Stomatol. 1970;67(6):217-21.
16. Cacciatore G, Ghislanzoni LT, Alvetro L, Giuntini V, Franchi L. Treatment and posttreatment effects induced by the Forsus appliance: A controlled clinical study. Angle Orthod. 2014;84(6):1010-7.
17. Casellas JC. Skeletal and dental changes with the acrylic splint Herbst appliance. M.Sc. Thesis, Ann Arbor: West Virginia University. 2001.
18. Chen DR, McGorray SP, Dolce C, Wheeler TT. Effect of early Class II treatment on the incidence of incisor trauma. Am J Orthod Dentofacial Orthop. 2011;140(4):e155-60.
19. Chhibber A, Upadhyay M, Uribe F, Nanda R. Long-term stability of Class II correction with the Twin Force Bite Corrector. J Clin Orthod. 2010;44(6):363-76.
20. Cozza P, De Toffol L. Functional appliance treatment of severe Class II malocclusion in the early mixed dentition. J Clin Orthod. 2003;37(2):69-74.
21. Craig WH. Cephalometric Evaluation of the Chateau Functional Appliance. M.Sc. in Dentistry Thesis, Ann Arbor: Baylor College of Dentistry. 1977.
22. Criswell GR. Skeletal and dental effects of a novel application of the MARA appliance in adolescent patients with Class II malocclusions. M.Sc. Thesis, Ann Arbor: University of California, San Francisco. 2011.

23. Dalci O, Altug AT, Memikoglu UT. Treatment effects of a twin-force bite corrector versus an activator in comparison with an untreated Class II sample: a preliminary report. Aust Orthod J. 2014;30(1):45-53.
24. DeVincenzo JP. Changes in mandibular length before, during, and after successful orthopedic correction of Class II malocclusions, using a functional appliance. Am J Orthod Dentofacial Orthop. 1991;99(3):241-57.
25. Dolce C, McGorray SP, Brazeau L, King GJ, Wheeler TT. Timing of Class II treatment: skeletal changes comparing 1-phase and 2-phase treatment. Am J Orthod Dentofacial Orthop. 2007;132(4):481-9.
26. Dolce C, Schader RE, McGorray SP, Wheeler TT. Centrographic analysis of 1-phase versus 2-phase treatment for Class II malocclusion. Am J Orthod Dentofacial Orthop. 2005;128(2):195-200.
27. Dos Santos-Pinto PR, Martins LP, dos Santos-Pinto A, Gandini Júnior LG, Raveli DB, dos Santos-Pinto CCM. Mandibular growth and dentoalveolar development in the treatment of Class II, division 1, malocclusion using balters bionator according to the skeletal maturation. Dental Press J Orthod. 2013;18(4):43-52.
28. Drage KJ, Hunt NP. Overjet relapse following functional appliance therapy. Br J Orthod. 1990;17(3):205-13.
29. Ehmer U, Tabanci J. Growth and therapy in Angle class II/2--a longitudinal cephalometric study. Fortschr Kieferorthop. 1990;51(4):208-12.
30. Falck F. Sagittal and vertical changes in mandibular retrognathism. A teleradiological longitudinal study of patients with functional regulators compared to a control group. Stomatol DDR. 1983;33(3):182-95.
31. Faxen Sepanian V, Paulsson-Bjornsson L, Kjellberg H. A long-term controlled follow-up study of objective treatment need on young adults treated with functional appliances. Swed Dent J. 2014;38(1):39-46.
32. Filip P. Late result in jaw orthopedic treatment by the Andresen-Haupls system. Den Nor

Tannlaegeforen Tid. 1970;80(5):326-34.

33. Flores-Mir C, Barnett G, Higgins DW, Heo G, Major PW. Short-term skeletal and dental effects of the Xbow appliance as measured on lateral cephalograms. Am J Orthod Dentofacial Orthop. 2009;136(6):822-32.
34. Foncatti CF, Castanha Henriques JF, Janson G, Caldas W, Garib DG. Long-term stability of Class II treatment with the Jasper jumper appliance. Am J Orthod Dentofacial Orthop. 2017;152(5):663-71.
35. Franchi L, Baccetti T, McNamara JA. Treatment and posttreatment effects of acrylic splint Herbst appliance therapy. Am J Orthod Dentofacial Orthop. 1999;115(4):429-38.
36. Franchi L, Baccetti T. Prediction of individual mandibular changes induced by functional jaw orthopedics followed by fixed appliances in Class II patients. Angle Orthod. 2006;76(6):950-4.
37. Franchi L, Baccetti T, Giuntini V, Masucci C, Vangelisti A, Defraia E. Outcomes of two-phase orthodontic treatment of deepbite malocclusions. Angle Orthod. 2011;81(6):945-52.
38. Franchi L, Pavoni C, Faltin K, Bigliazzi R, Gazzani F, Cozza P. Thin-plate spline analysis of mandibular shape changes induced by functional appliances in Class II malocclusion: a long-term evaluation. J Orofac Orthop. 2016;77(5):325-33.
39. Frankel R, Frankel C. A functional approach to treatment of skeletal open bite. Am J Orthod. 1983;84(1):54-68.
40. Fry JR. A comparison of the soft tissue outcomes of one and two phase Class II orthodontic treatment. M.Sc. Thesis, Ann Arbor: University of Southern California. 2006.
41. Ghislanzoni LT, Toll DE, Defraia E, Baccetti T, Franchi L. Treatment and posttreatment outcomes induced by the Mandibular Advancement Repositioning Appliance; a controlled clinical study. Angle Orthod. 2011;81(4):684-91.
42. Han S, Choi YJ, Chung CJ, Kim JY, Kim KH. Long-term pharyngeal airway changes after bionator treatment in adolescents with skeletal Class II malocclusions. Korean J Orthod. 2014;44(1):13-9.

43. Hansen K, Pancherz H. Long-term effects of Herbst treatment in relation to normal growth development: a cephalometric study. Eur J Orthod. 1992;14(4):285-95.

44. Humphrey MP. Post-treatment stability of the dentoalveolar effects caused by the Forsus fatigue resistant device. M.Sc. in Dentistry Thesis, Ann Arbor: Saint Louis University. 2016.

45. Jacob HB, dos Santos-Pinto A, Buschang PH. Dental and skeletal components of Class II open bite treatment with a modified Thurow appliance. Dental Press J Orthod. 2014;19(1):19-25.

46. Jakobsone G, Latkauskiene D, McNamara JA, Jr. Mechanisms of Class II correction induced by the crown Herbst appliance as a single-phase Class II therapy: 1 year follow-up. Prog Orthod. 2013;14:27.

47. Janson G, Nakamura A, Chiqueto K, Castro R, de Freitas MR, Henriques JF. Treatment stability with the eruption guidance appliance. Am J Orthod Dentofacial Orthop. 2007;131(6):717-28.

48. Johannesen B. A cephalometric follow-up study of overjet and incisor stability in treated class II, division 1. Trans Eur Orthod Soc. 1972:231-49.

49. Karlowska I. Late results of treatment with elastic appliances of the author's own design. Czas Stomatol. 1971;24(2):207-13.

50. Keeling SD, Wheeler TT, King GJ, Garvan CW, Cohen DA, Cabassa S, et al. Anteroposterior skeletal and dental changes after early Class II treatment with bionators and headgear. Am J Orthod Dentofacial Orthop. 1998;113(1):40-50.

51. Keski-Nisula K, Keski-Nisula L, Salo H, Voipio K, Varrela J. Dentofacial changes after orthodontic intervention with eruption guidance appliance in the early mixed dentition. Angle Orthodontist. 2008;78(2):324-31.

52. Knight H. The effects of three methods of orthodontic appliancetherapy on some commonly used cephalometric angular variables. Am J Orthod Dentofacial Orthop. 1988;93(3):237-44.

53. Koroluk LD, Tulloch JF, Phillips C. Incisor trauma and early treatment for Class II Division 1 malocclusion. Am J Orthod Dentofacial Orthop. 2003;123(2):117-25; discussion 25-6.
54. Lall R, Kumar GA, Maheshwari A, Kumar M. A retrospective cephalometric evaluation of dental changes with activator and activator headgear combination in the treatment of skeletal class II malocclusion. J Contemp Dent Pract. 2011;12(1):14-8.
55. Lima KJ, Henriques JF, Janson G, Pereira SC, Neves LS, Cancado RH. Dentoskeletal changes induced by the Jasper jumper and the activator-headgear combination appliances followed by fixed orthodontic treatment. Am J Orthod Dentofacial Orthop. 2013;143(5):684-94.
56. Livieratos FA, Johnston Jr LE. A comparison of one-stage and two-stage nonextraction alternatives in matched Class II samples. Am J Orthod Dentofacial Orthop. 1995;108(2):118-31.
57. Luder HU. Skeletal profile changes related to two patterns of activator effects. Am J Orthod. 1982;81(5):390-6.
58. Lux CJ, Rubel J, Starke J, Conradt C, Stellzig PA, Komposch PG. Effects of early activator treatment in patients with class II malocclusion evaluated by thin-plate spline analysis. Angle Orthod. 2001;71(2):120-6.
59. Madone G, Ingervall B. Stability of results and function of the masticatory system in patients treated with the Herren type of activator. Eur J Orthod. 1984;6(2):92-106.
60. Madone G, Ingervall B. Stability of the results and function of the jaws after correction of distal bite using the Herren activator. Schweiz Monatsschr Zahnmed. 1984;94(5):453-61.
61. Mills CM, McCulloch KJ. Posttreatment changes after successful correction of Class II malocclusions with the twin block appliance. Am J Orthod Dentofacial Orthop. 2000;118(1):24-33.
62. Mongini F, Schmid W. Treatment of mandibular asymmetries during growth. A longitudinal study. Eur J Orthod. 1987;9(1):51-67.

63. Morris DO, Illing HM, Lee RT. A prospective evaluation of Bass, Bionator and Twin Block appliances. Part II--The soft tissues. Eur J Orthod. 1998;20(6):663-84.
64. Mortenson DR. Soft tissue changes from Herbst appliance treatment: A cephalometric longitudinal study. M.Sc. Thesis, Ann Arbor: University of Louisville. 2004.
65. Nelson B, Hagg U, Hansen K, Bendeus M. A long-term follow-up study of Class II malocclusion correction after treatment with Class II elastics or fixed functional appliances. Am J Orthod Dentofacial Orthop. 2007;132(4):499-503.
66. O'Brien K, Wright J, Conboy F, Appelbe P, Davies L, Connolly I, et al. Early treatment for Class II Division 1 malocclusion with the Twin-block appliance: a multi-center, randomized, controlled trial. Am J Orthod Dentofacial Orthop. 2009;135(5):573-9.
67. Omblus J, Malmgren O, Pancherz H, Hagg U, Hansen K. Long-term effects of Class II correction in Herbst and Bass therapy. Eur J Orthod. 1997;19(2):185-93.
68. Pancherz H. Relapse after activator treatment. A biometric, cephalometric, and electromyographic study of subjects with and without relapse of overjet. Am J Orthod. 1977;72(5):499-512.
69. Pancherz H, Hansen K. Occlusal changes during and after Herbst treatment: a cephalometric investigation. Eur J Orthod. 1986;8(4):215-28.
70. Pancherz H, Littmann C. Morphology and position of mandible in Herbst treatment. Cephalometric analysis of changes to end of growth period. Inf Orthod Kieferorthop. 1989;21(4):493-513.
71. Pancherz H, Anehus-Pancherz M. The headgear effect of the Herbst appliance: a cephalometric long-term study. Am J Orthod Dentofacial Orthop. 1993;103(6):510-20.
72. Pancherz H, Anehus-Pancherz M. Facial profile changes during and after Herbst appliance treatment. Eur J Orthod. 1994;16(4):275-86.
73. Pancherz H, Ruf S, Kohlhas P. "Effective condylar growth" and chin position changes in Herbst treatment: a cephalometric roentgenographic long-term study. Am J Orthod Dentofacial Orthop. 1998;114(4):437-46.

74. Pancherz H, Fischer S. Amount and direction of temporomandibular joint growth changes in Herbst treatment: a cephalometric long-term investigation. Angle Orthod. 2003;73(5):493-501.
75. Pancherz H, Bjerklin K, Hashemi K. Late adult skeletofacial growth after adolescent Herbst therapy: a 32-year longitudinal follow-up study. Am J Orthod Dentofacial Orthop. 2015;147(1):19-28.
76. Pancherz H, Bjerklin K. The Herbst appliance 32 years after treatment. J Clin Orthod. 2015;49(7):442-51.
77. Pangrazio MN, Pangrazio-Kulbersh V, Berger JL, Bayirli B, Movahhedian A. Treatment effects of the mandibular anterior repositioning appliance in patients with Class II skeletal malocclusions. Angle Orthod. 2012;82(6):971-7.
78. Pavoni C, Lombardo EC, Lione R, Faltin K, Jr., McNamara JA, Jr., Cozza P, et al. Treatment timing for functional jaw orthopaedics followed by fixed appliances: a controlled long-term study. Eur J Orthod. 2017.
79. Perillo L, Johnston LE, Jr., Ferro A. Permanence of skeletal changes after function regulator (FR-2) treatment of patients with retrusive Class II malocclusions. Am J Orthod Dentofacial Orthop. 1996;109(2):132-9.
80. Perillo L, Castaldo MI, Cannavale R, Longobardi A, Grassia V, Rullo R, et al. Evaluation of long-term effects in patients treated with Frankel-2 appliance. Eur J Paediatr Dent. 2011;12(4):261-6.
81. Phelan A, Tarraf NE, Taylor P, Honscheid R, Drescher D, Baccetti T, et al. Skeletal and dental outcomes of a new magnetic functional appliance, the Sydney Magnoglide, in Class II correction. Am J Orthod Dentofacial Orthop. 2012;141(6):759-72.
82. Righellis EG. Treatment effects of Frankel, activator and extraoral traction appliances. Angle Orthod. 1983;53(2):107-21.
83. Sander FG, Wichelhaus A. Skeletal and dental changes during the use of the bite-jumping plate. A cephalometric comparison with an untreated Class-II group. Fortschr

Kieferorthop. 1995;56(3):127-39.

84. Sawrie DC. Cephalometric evaluation of Bionator therapy in the early treatment of Class II malocclusions. Master of Dental Science Thesis, Ann Arbor: The University of Tennessee Health Science Center. 2007.
85. Scalzone A, D'Apuzzo F, Scalzone PP, Vitale V, Cannavale R, Perillo L. Twin Block treatment in growing Class II patients: Dento-skeletal effects. Dental Cadmos. 2015;83(10):654-9.
86. Schadlbauer E. Long-term results after activator treatment. Inf Orthod Kieferorthop. 1984;16(4):419-29.
87. Schutz-Fransson U, Bjerklin K, Lindsten R. Long-term follow-up of orthodontically treated deep bite patients. Eur J Orthod. 2006;28(5):503-12.
88. Siara-Olds NJ, Pangrazio-Kulbersh V, Berger J, Bayirli B. Long-term dentoskeletal changes with the Bionator, Herbst, Twin Block, and MARA functional appliances. Angle Orthod. 2010;80(1):18-29.
89. Sivakumar A, Valiathan A. Stability and functional appliances. Am J Orthod Dentofacial Orthop. 2005;128(6):687.
90. Stuber P. The stimulation of the rotation of the mandible with Angle Class II/1 and II/2 malocclusion with functional regulators and activators as compared to the control group. Stomatol DDR. 1990;40(3):112-4.
91. Stuber P. The possibility of stimulating mandibular growth in mandibular retrognathism with functional regulators and activators compared to a control group--a 6-year cephalometric longitudinal study. Fortschr Kieferorthop. 1990;51(6):361-5.
92. Thompson WD. Dental and skeletal changes from Herbst appliance treatment: A cephalometric longitudinal study. M.Sc. Thesis, Ann Arbor: University of Louisville. 2001.
93. Tomblyn T. A Radiographic Study of Patients Treated with the Reinforced Banded Herbst Appliance. M.Sc. Thesis, Ann Arbor: West Virginia University. 2015.

94. Tomblyn T, Rogers M, Andrews L, 2nd, Martin C, Tremont T, Gunel E, et al. Cephalometric study of Class II Division 1 patients treated with an extended-duration, reinforced, banded Herbst appliance followed by fixed appliances. Am J Orthod Dentofacial Orthop. 2016;150(5):818-30.
95. Tulloch JF, Phillips C, Proffit WR. Benefit of early Class II treatment: progress report of a two-phase randomized clinical trial. Am J Orthod Dentofacial Orthop. 1998;113(1):62-72, quiz 3-4.
96. Ulusoy C, Canigur Bavbek N, Tuncer BB, Tuncer C, Turkoz C, Gencturk Z. Evaluation of airway dimensions and changes in hyoid bone position following class II functional therapy with activator. Acta Odontol Scand. 2014;72(8):917-25.
97. Valant JR. Cephalometric and Dental Evaluation of the Herbst Appliance in Class II Patients. M.Sc. in Dentistry, Ann Arbor: Baylor College of Dentistry. 1983.
98. VanLaecken R, Martin CA, Dischinger T, Razmus T, Ngan P. Treatment effects of the edgewise Herbst appliance: A cephalometric and tomographic investigation. Am J Orthod Dentofacial Orthop. 2006;130(5):582-93.
99. Vardimon AD, Koklu S, Iseri H, Shpack N, Fricke J, Mete L. An assessment of skeletal and dental responses to the functional magnetic system (FMS). Am J Orthod Dentofacial Orthop. 2001;120(4):416-26.
100. Voudouris JC, Woodside DG, Altuna G, Kuftinec MM, Angelopoulos G, Bourque PJ. Condyle-fossa modifications and muscle interactions during Herbst treatment, part 1. New technological methods. Am J Orthod Dentofacial Orthop. 2003;123(6):604-13.
101. Voudouris JC, Woodside DG, Altuna G, Angelopoulos G, Bourque PJ, Lacouture CY. Condyle-fossa modifications and muscle interactions during Herbst treatment, Part 2. Results and conclusions. Am J Orthod Dentofacial Orthop. 2003;124(1):13-29.
102. Weschler D, Pancherz H. Efficiency of three mandibular anchorage forms in Herbst treatment: a cephalometric investigation. Angle Orthod. 2005;75(1):23-7.
103. Wheeler TT, McGorray SP, Dolce C, Taylor MG, King GJ. Effectiveness of early

treatment of Class II malocclusion. Am J Orthod Dentofacial Orthop. 2002;121(1):9-17.

104. Wortham JR, Dolce C, McGorray SP, Le H, King GJ, Wheeler TT. Comparison of arch dimension changes in 1-phase vs 2-phase treatment of Class II malocclusion. Am J Orthod Dentofacial Orthop. 2009;136(1):65-74.

105. Yassaei S, Tabatabaei Z, Ghafurifard R. Stability of pharyngeal airway dimensions: tongue and hyoid changes after treatment with a functional appliance. Int J Orthod Milwaukee. 2012;23(1):9-15.

106. Yassaei S, Jamilian A, Joshan N. Effects of Twin-Block and Faramand-LL appliances on soft tissue profile in the treatment of Class II division 1 malocclusion. Int J Orthod Milwaukee. 2014;25(4):57-62.

107. Yuksel S, Kaygisiz E, Ulusoy C, Keykubat A. Post-treatment evaluation of a magnetic activator device in Class II high-angle malocclusions. Eur J Orthod. 2010;32(4):425-9.

108. Zelderloo A, Cadenas De Llano-Pérula M, Verdonck A, Fieuws S, Willems G. Cephalometric appraisal of Class II treatment effects after functional and fixed appliances: A retrospective study. Eur J Orthod. 2017;39(3):334-41.
