# Supplementary material for: Long-term effects of functional appliances in treated versus untreated patients with Class II malocclusion: A systematic review and meta-analysis
Source: PLoS One. 2019 Sep 6;14(9):e0221624. doi: 10.1371/journal.pone.0221624 (PMC6730901; doi:10.1371/journal.pone.0221624)
Supplement: S3 Appendix — (PDF) [file pone.0221624.s009.pdf]

S3 Appendix. The forest plots concerning redundant or non-statistically significant results.

Maxillary/upper jaw changes

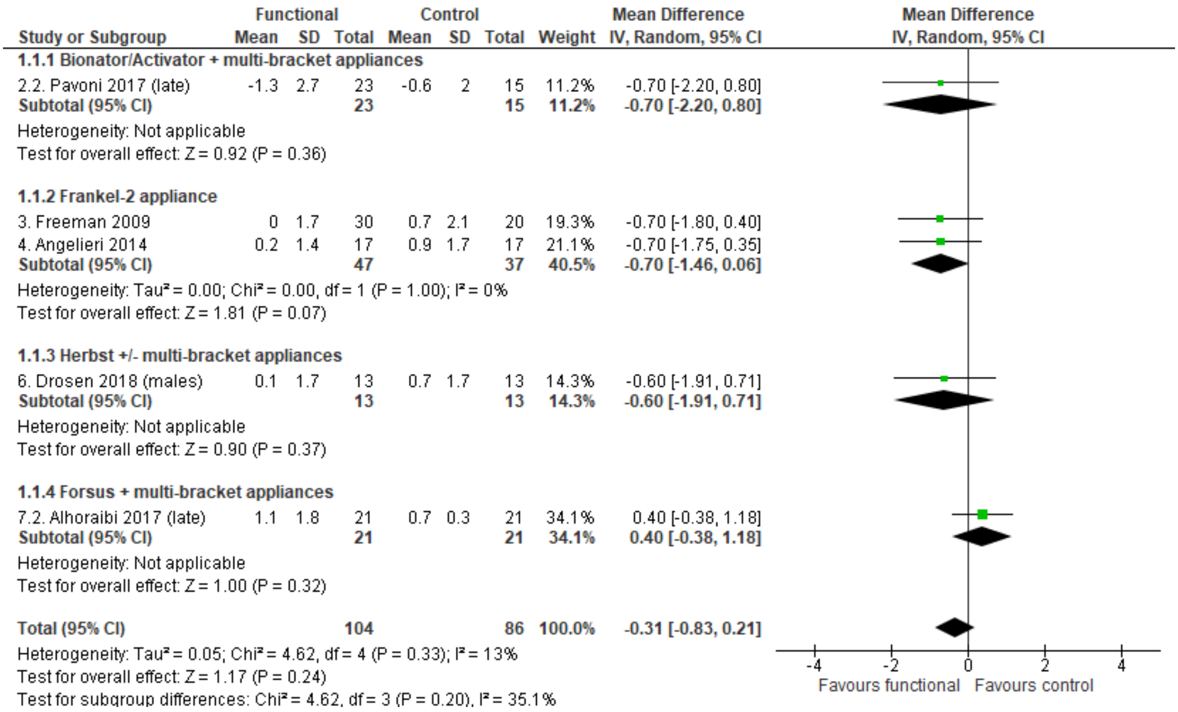

Figure 1. Meta-analysis; Outcome: SNA angle; Time point: above 18 years of age.

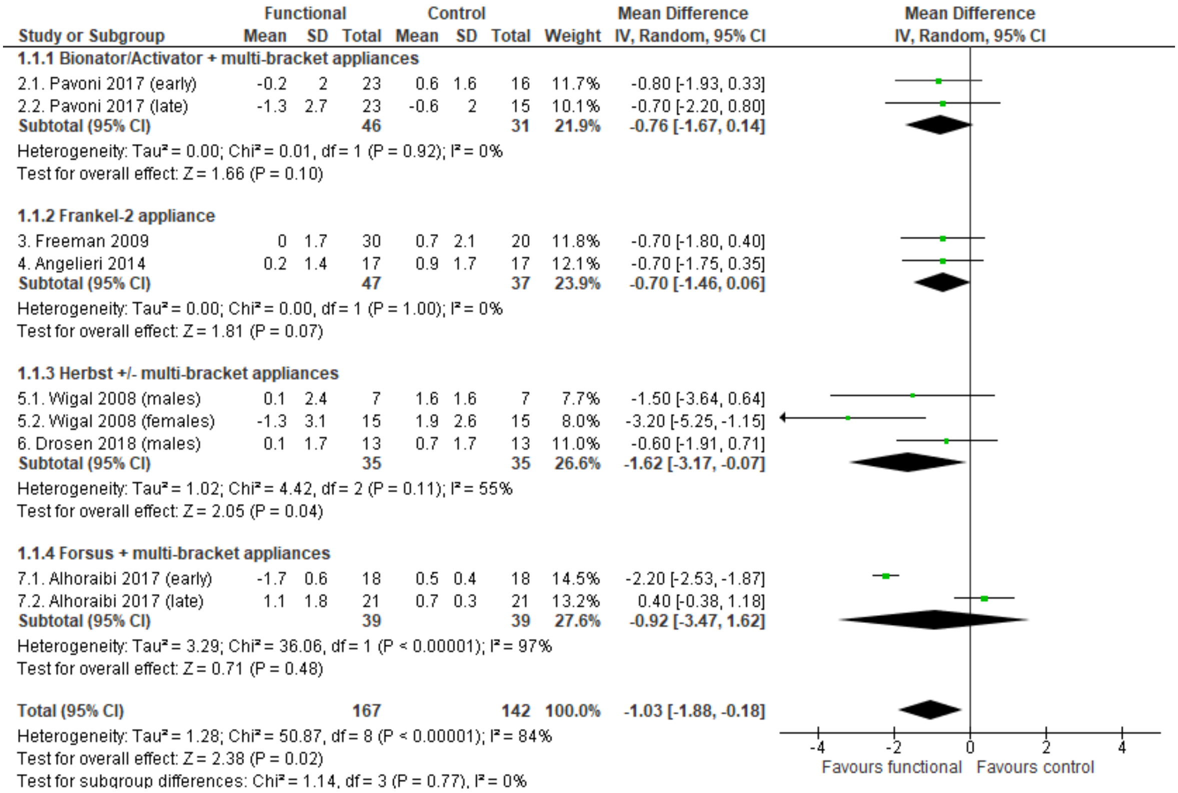

Figure 2. Meta-analysis; Outcome: SNA angle; Time point: After a post-retention period of at least 3 years.

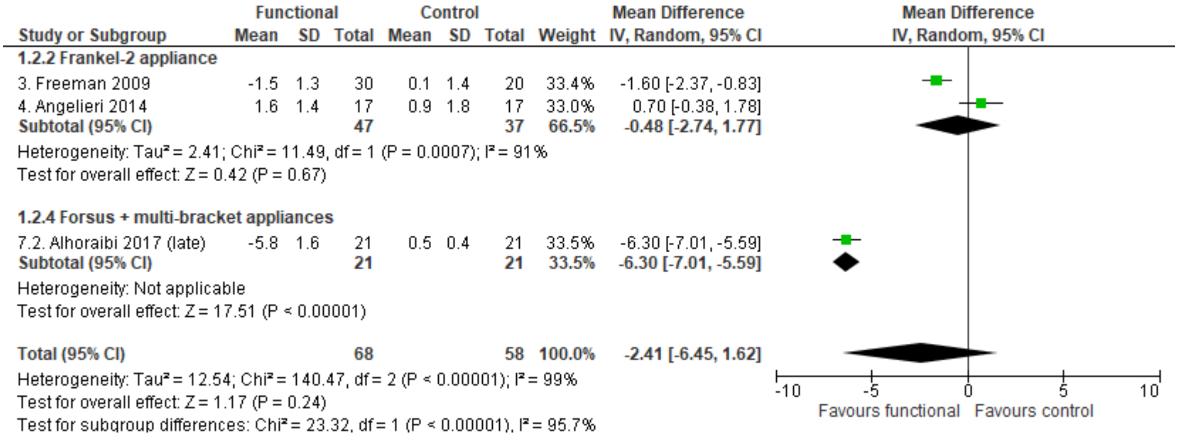

Figure 3. Meta-analysis; Outcome: A to N perpendicular distance; Time point: above 18 years of age.

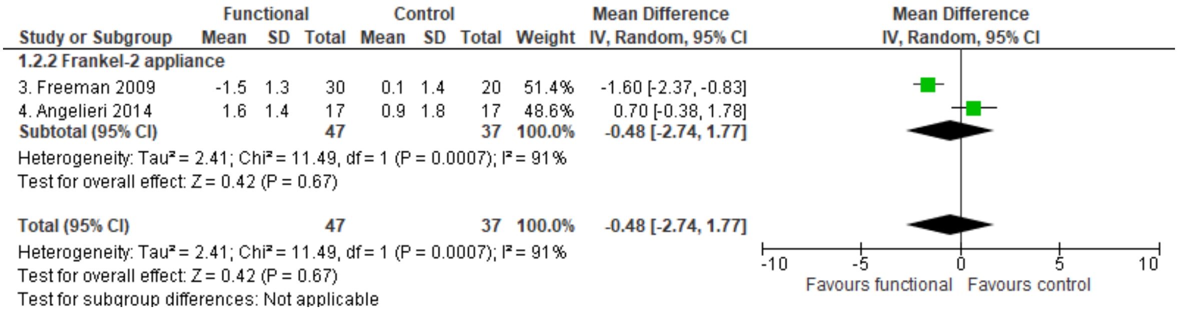

Figure 4. Meta-analysis; Outcome: A to N perpendicular distance; Time point: end of growth according to the CVM method.

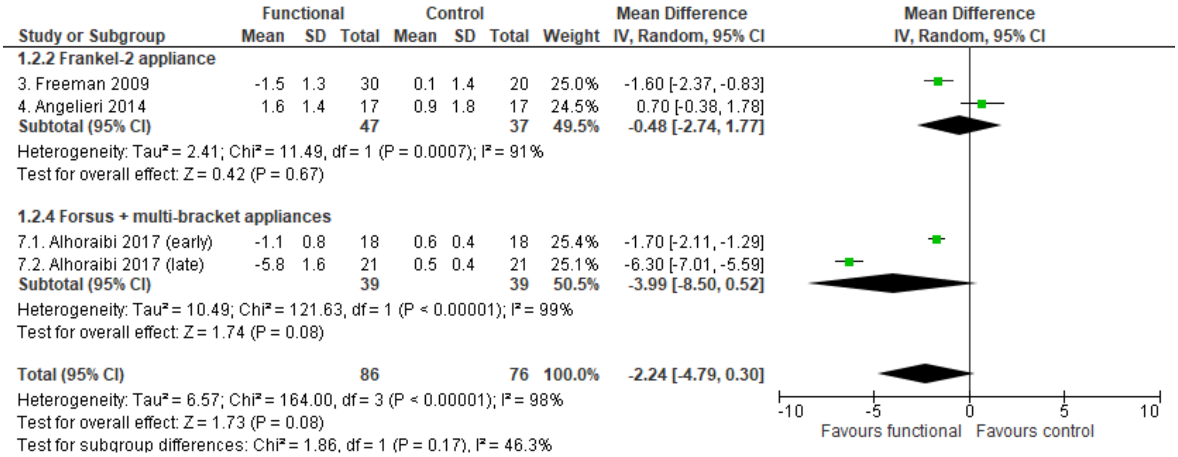

Figure 5. Meta-analysis; Outcome: A to N perpendicular distance; Time point: After a post-retention period of at least 3 years.

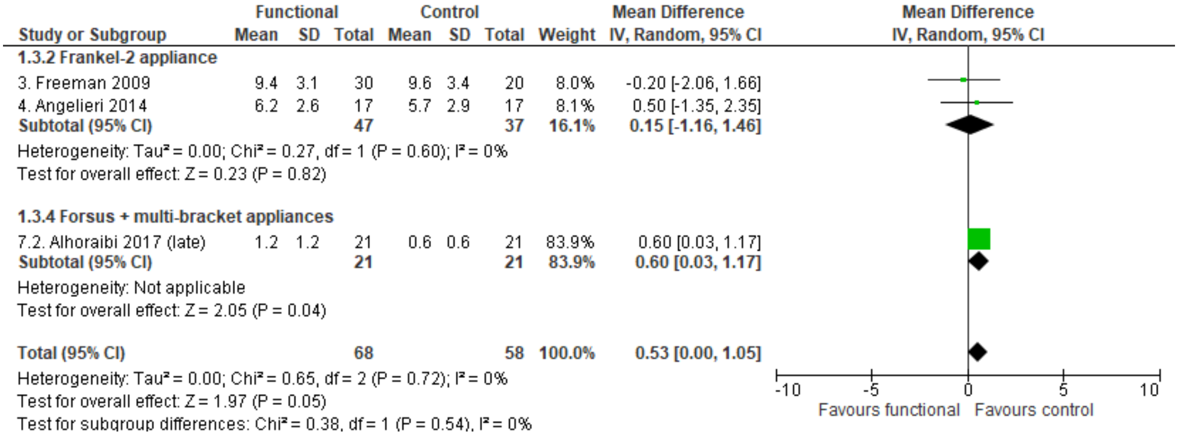

Figure 6. Meta-analysis; Outcome: Co-A distance; Time point: above 18 years of age.

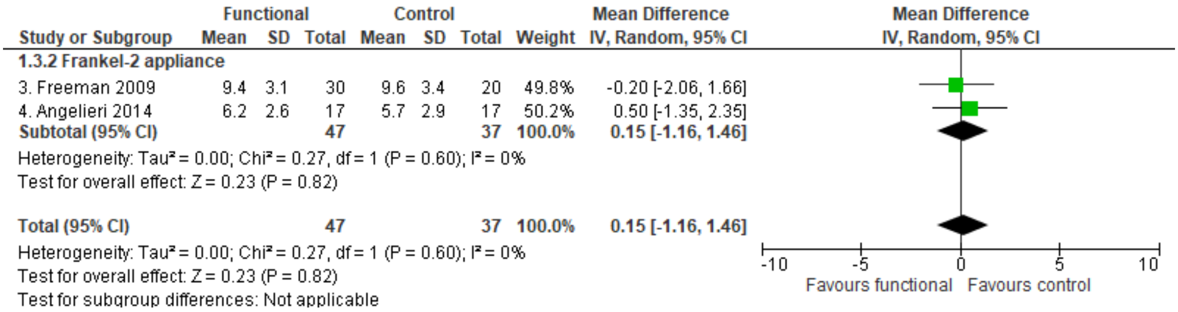

Figure 7. Meta-analysis; Outcome: Co-A distance; Time point: end of growth according to the CVM method.

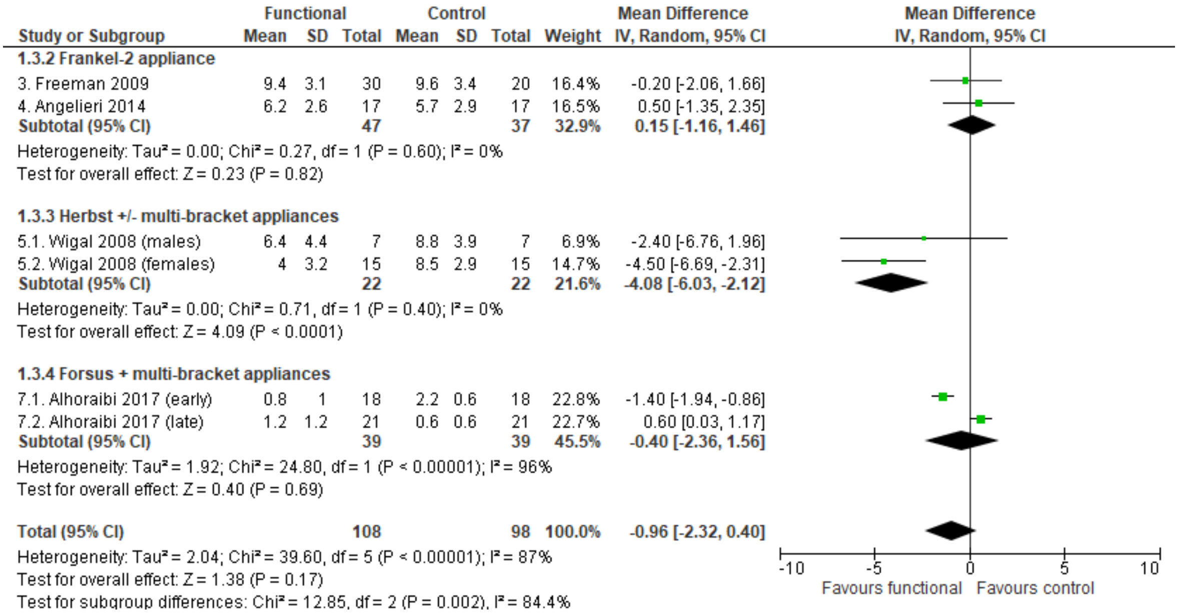

Figure 8. Meta-analysis; Outcome: Co-A distance; Time point: After a post-retention period of at least 3 years.

Mandibular/lower jaw changes

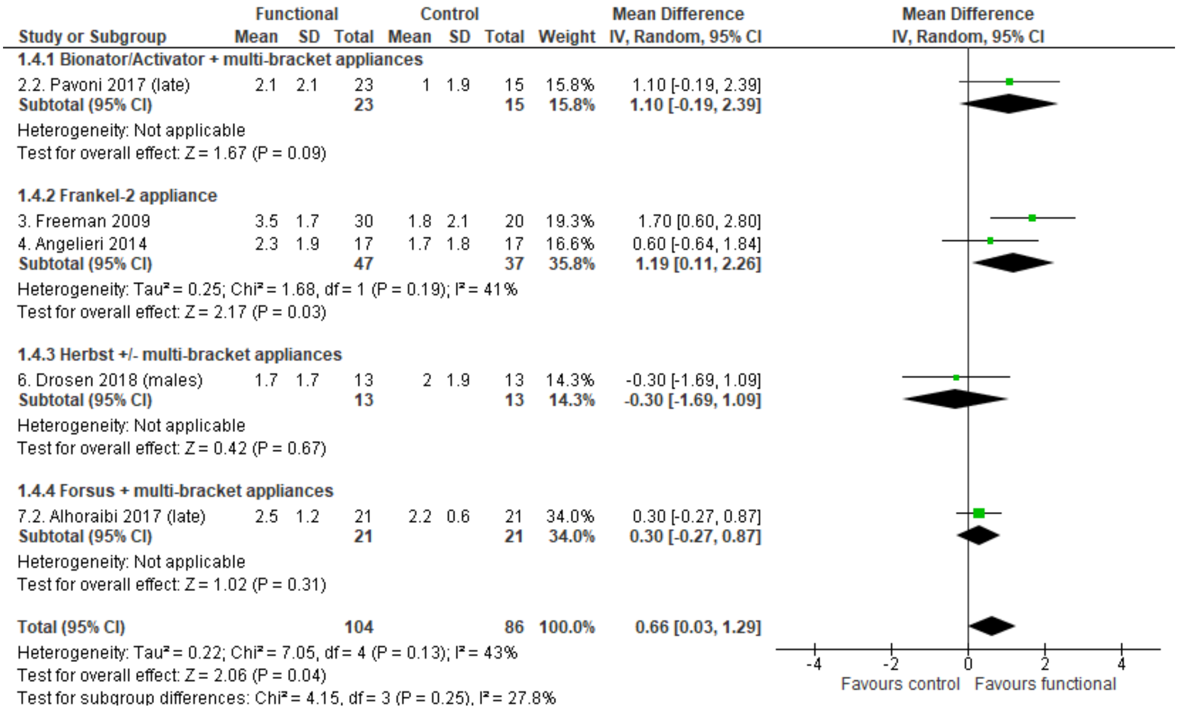

Figure 9. Meta-analysis; Outcome: SNB angle; Time point: above 18 years of age.

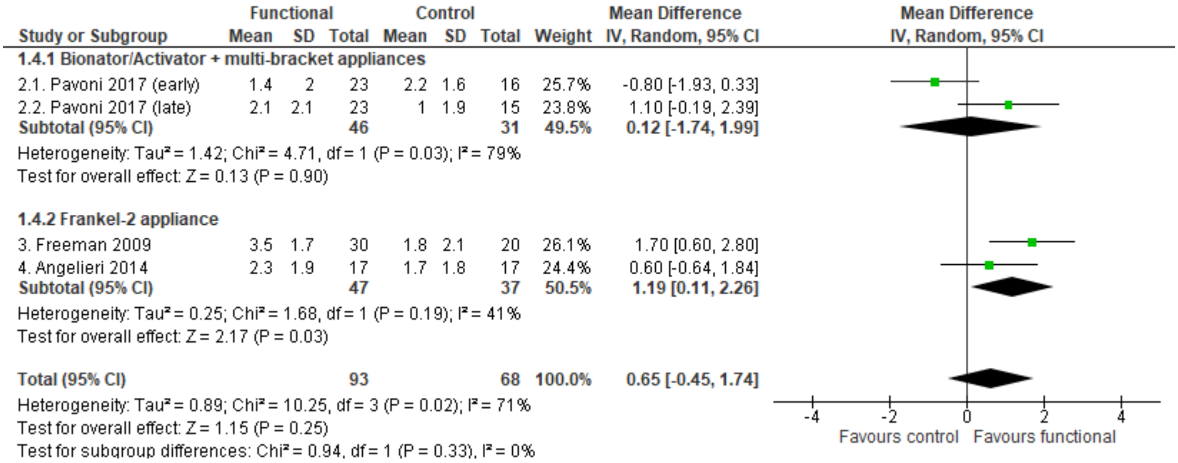

Figure 10. Meta-analysis; Outcome: SNB angle; Time point: end of growth according to the CVM method.

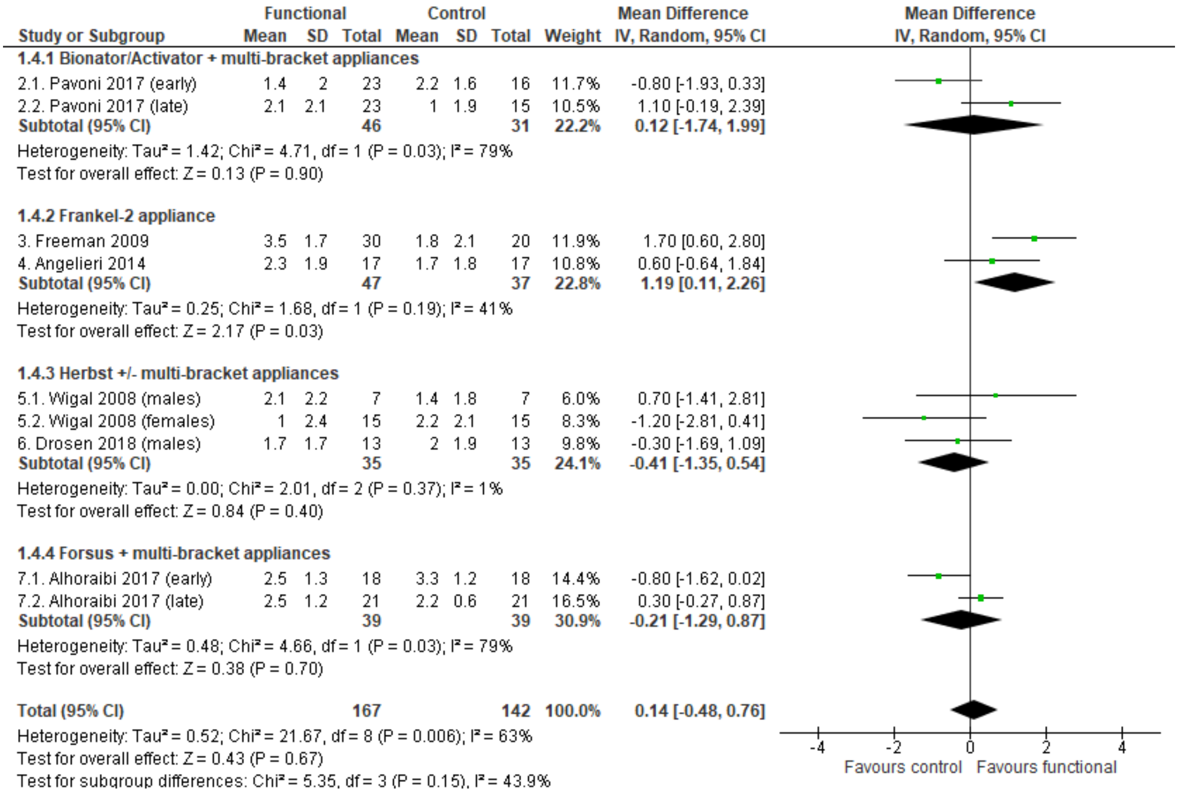

Figure 11. Meta-analysis; Outcome: SNB angle; Time point: After a post-retention period of at least 3 years.

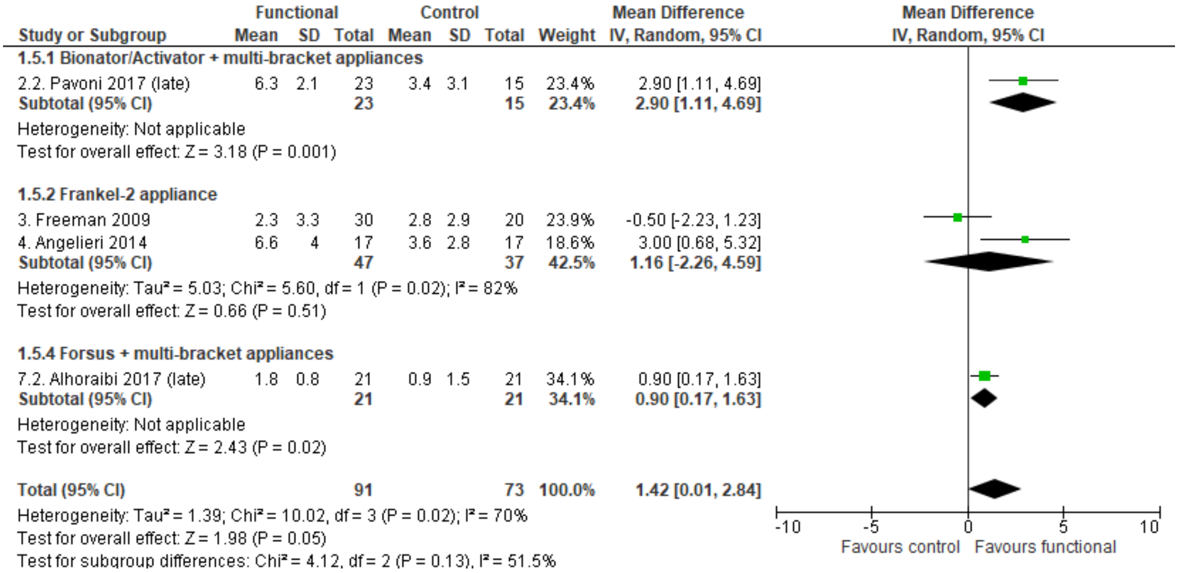

Figure 12. Meta-analysis; Outcome: Pg to N perp distance; Time point: above 18 years of age.

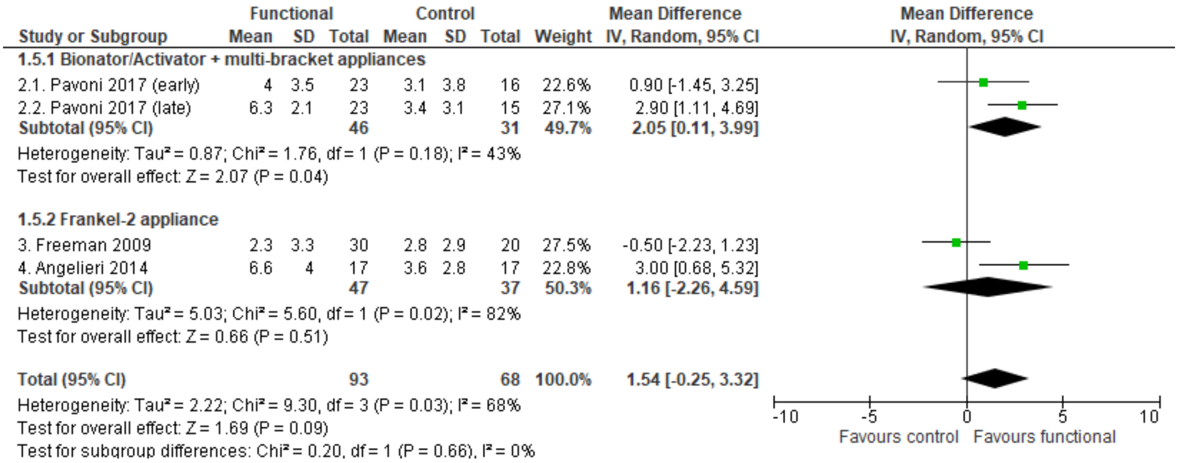

Figure 13. Meta-analysis; Outcome: Pg to N perp distance; Time point: end of growth according to the CVM method.

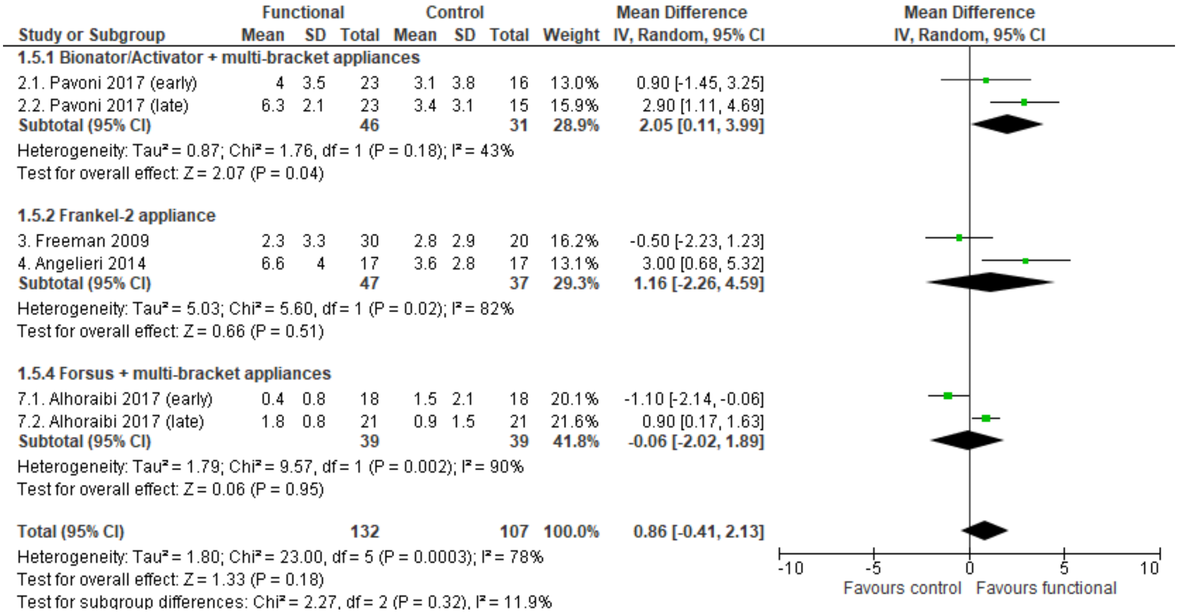

Figure 14. Meta-analysis; Outcome: Pg to N perpendicular distance; Time point: After a post-retention period of at least 3 years.

Study or Subgroup	Functional Mean	Functional SD	Functional Total	Control Mean	Control SD	Control Total	Weight	Mean Difference IV, Random, 95% CI
**1.6.1 Bionator/Activator + multi-bracket appliances**								
2.1. Pavoni 2017 (early)	15.6	4.3	23	16.2	4.4	16	11.4%	-0.60 [-3.38, 2.18]
2.2. Pavoni 2017 (late)	19	3.4	23	13.9	2.3	15	13.5%	5.10 [3.29, 6.91]
**Subtotal (95% CI)**			**46**			**31**	**24.9%**	**2.35 [-3.23, 7.93]**
Heterogeneity: Tau² = 14.81; Chi² = 11.32, df = 1 (P = 0.0008); I² = 91%								
Test for overall effect: Z = 0.83 (P = 0.41)								
**1.6.2 Frankel-2 appliance**								
3. Freeman 2009	19.1	4.5	30	16.3	4.2	20	12.2%	2.80 [0.35, 5.25]
4. Angelieri 2014	15.2	4.3	17	11.5	4.3	17	11.2%	3.70 [0.81, 6.59]
**Subtotal (95% CI)**			**47**			**37**	**23.4%**	**3.18 [1.31, 5.04]**
Heterogeneity: Tau² = 0.00; Chi² = 0.22, df = 1 (P = 0.64); I² = 0%								
Test for overall effect: Z = 3.33 (P = 0.0009)								
**1.6.3 Herbst +/- multi-bracket appliances**								
5.1. Wigal 2008 (males)	15.4	4.5	7	14.1	3	7	8.9%	1.30 [-2.71, 5.31]
5.2. Wigal 2008 (females)	9.8	2.3	15	13.3	3	15	13.3%	-3.50 [-5.41, -1.59]
**Subtotal (95% CI)**			**22**			**22**	**22.2%**	**-1.44 [-6.09, 3.22]**
Heterogeneity: Tau² = 8.95; Chi² = 4.49, df = 1 (P = 0.03); I² = 78%								
Test for overall effect: Z = 0.60 (P = 0.55)								
**1.6.4 Forsus + multi-bracket appliances**								
7.1. Alhoraibi 2017 (early)	3.7	2.2	18	0.1	0.6	18	14.7%	3.60 [2.55, 4.65]
7.2. Alhoraibi 2017 (late)	1.6	2.2	21	0	0.6	21	14.8%	1.60 [0.62, 2.58]
**Subtotal (95% CI)**			**39**			**39**	**29.5%**	**2.59 [0.63, 4.55]**
Heterogeneity: Tau² = 1.73; Chi² = 7.46, df = 1 (P = 0.006); I² = 87%								
Test for overall effect: Z = 2.59 (P = 0.010)								
**Total (95% CI)**			**154**			**129**	**100.0%**	**1.79 [-0.05, 3.64]**
Heterogeneity: Tau² = 5.73; Chi² = 57.49, df = 7 (P < 0.00001); I² = 88%								
Test for overall effect: Z = 1.90 (P = 0.06)								
Test for subgroup differences: Chi² = 3.25, df = 3 (P = 0.35), I² = 7.7%								

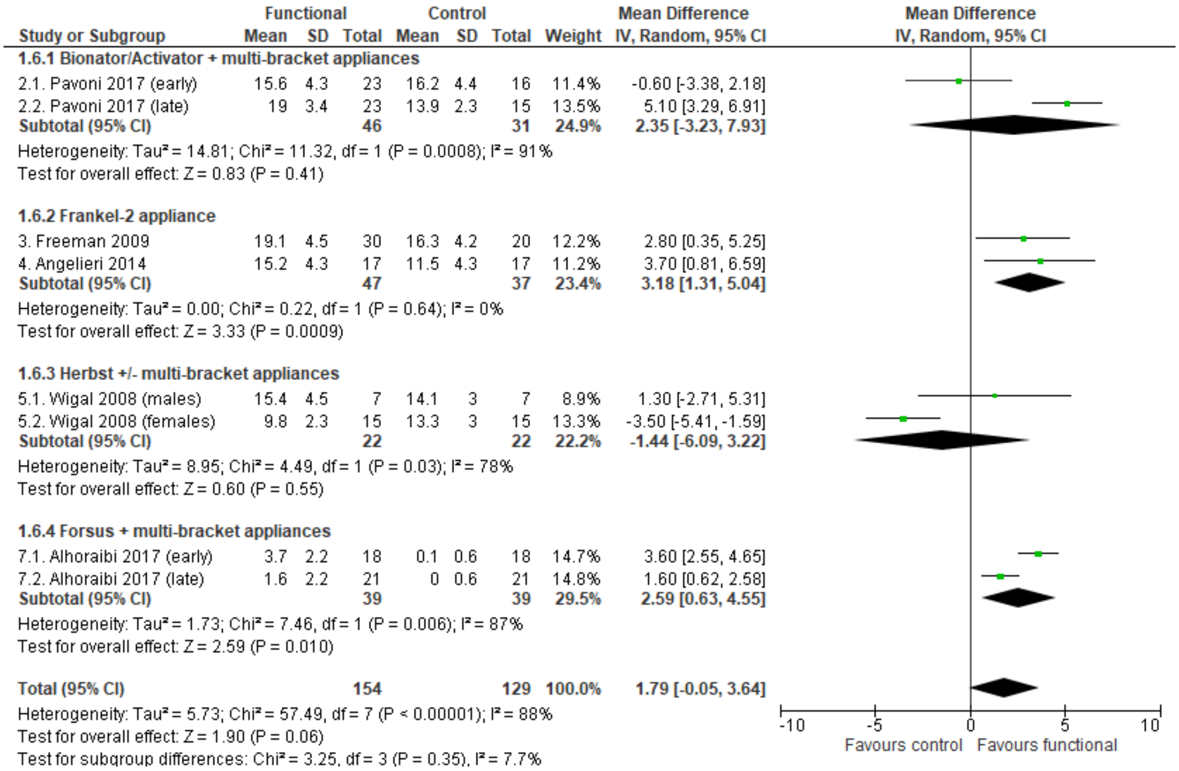

Figure 15. Meta-analysis; Outcome: Co-Gn distance; Time point: After a post-retention period of at least 3 years.

Maxillo-mandibular changes

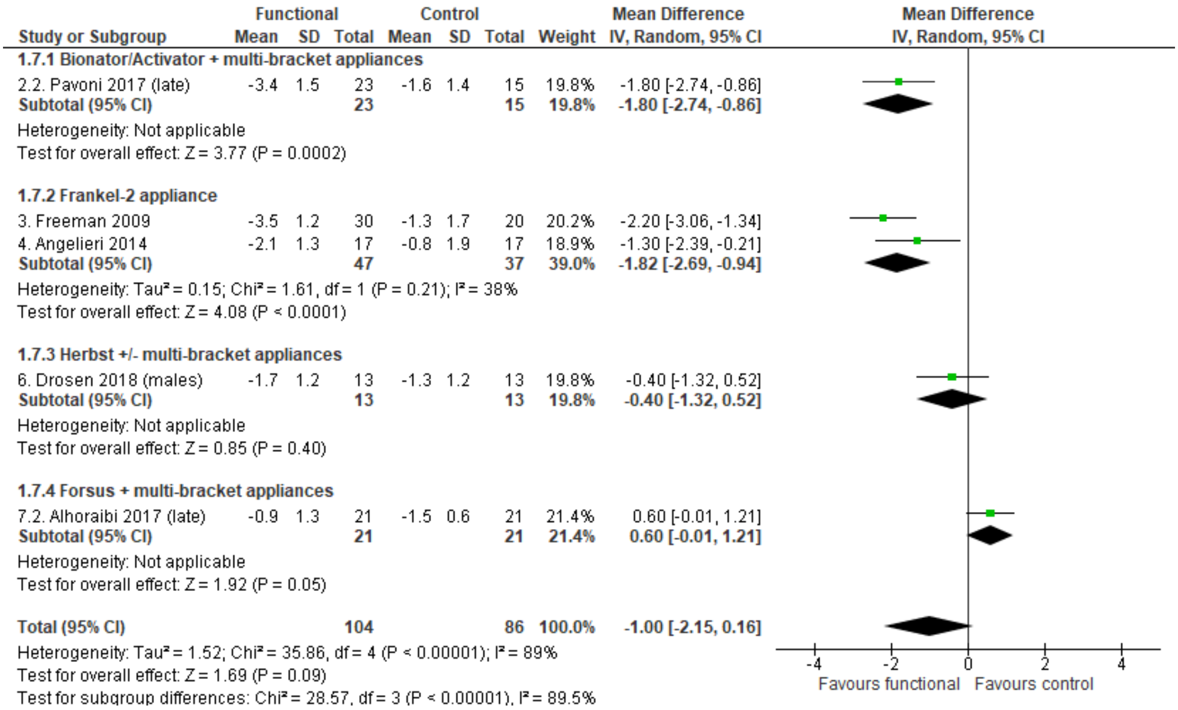

Figure 16. Meta-analysis; Outcome: ANB angle; Time point: above 18 years of age.

Study or Subgroup	Functional Mean	Functional SD	Functional Total	Control Mean	Control SD	Control Total	Weight	Mean Difference IV, Random, 95% CI
**1.7.1 Bionator/Activator + multi-bracket appliances**								
1. Wieslander 1979	-2.3	1.4	23	-0.5	1	23	10.9%	-1.80 [-2.50, -1.10]
2.1. Pavoni 2017 (early)	-1.6	1.2	23	-1.6	1.1	16	10.8%	0.00 [-0.73, 0.73]
2.2. Pavoni 2017 (late)	-3.4	1.5	23	-1.6	1.4	15	10.0%	-1.80 [-2.74, -0.86]
**Subtotal (95% CI)**			**69**			**54**	**31.7%**	**-1.19 [-2.41, 0.04]**
Heterogeneity: Tau² = 1.01; Chi² = 14.69, df = 2 (P = 0.0006); I² = 86%								
Test for overall effect: Z = 1.90 (P = 0.06)								
**1.7.2 Frankel-2 appliance**								
3. Freeman 2009	-3.5	1.2	30	-1.3	1.7	20	10.3%	-2.20 [-3.06, -1.34]
4. Angelieri 2014	-2.1	1.3	17	-0.8	1.9	17	9.4%	-1.30 [-2.39, -0.21]
**Subtotal (95% CI)**			**47**			**37**	**19.8%**	**-1.82 [-2.69, -0.94]**
Heterogeneity: Tau² = 0.15; Chi² = 1.61, df = 1 (P = 0.21); I² = 38%								
Test for overall effect: Z = 4.08 (P < 0.0001)								
**1.7.3 Herbst +/- multi-bracket appliances**								
5.1. Wigal 2008 (males)	-2.1	1.6	7	0.2	1.2	7	7.9%	-2.30 [-3.78, -0.82]
5.2. Wigal 2008 (females)	-2.3	1.7	15	-0.3	1.4	15	9.4%	-2.00 [-3.11, -0.89]
6. Drosen 2018 (males)	-1.7	1.2	13	-1.3	1.2	13	10.1%	-0.40 [-1.32, 0.52]
**Subtotal (95% CI)**			**35**			**35**	**27.4%**	**-1.48 [-2.72, -0.25]**
Heterogeneity: Tau² = 0.84; Chi² = 6.92, df = 2 (P = 0.03); I² = 71%								
Test for overall effect: Z = 2.35 (P = 0.02)								
**1.7.4 Forsus + multi-bracket appliances**								
7.1. Alhoraibi 2017 (early)	-3.3	1.6	18	-2.9	1.3	18	10.0%	-0.40 [-1.35, 0.55]
7.2. Alhoraibi 2017 (late)	-0.9	1.3	21	-1.5	0.6	21	11.2%	0.60 [-0.01, 1.21]
**Subtotal (95% CI)**			**39**			**39**	**21.1%**	**0.17 [-0.80, 1.14]**
Heterogeneity: Tau² = 0.33; Chi² = 3.00, df = 1 (P = 0.08); I² = 67%								
Test for overall effect: Z = 0.34 (P = 0.73)								
**Total (95% CI)**			**190**			**165**	**100.0%**	**-1.11 [-1.82, -0.40]**
Heterogeneity: Tau² = 1.07; Chi² = 57.36, df = 9 (P < 0.00001); I² = 84%								
Test for overall effect: Z = 3.08 (P = 0.002)								
Test for subgroup differences: Chi² = 9.54, df = 3 (P = 0.02), I² = 68.5%								

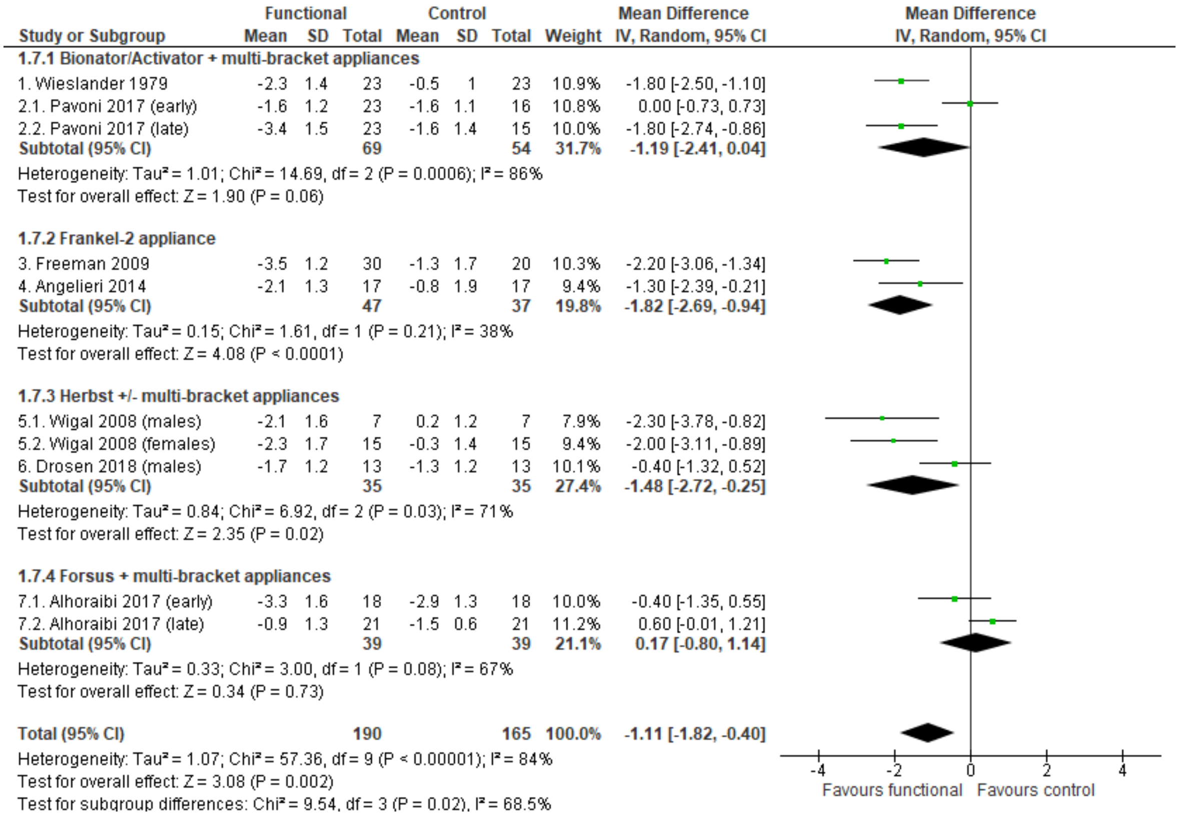

Figure 17. Meta-analysis; Outcome: ANB angle; Time point: After a post-retention period of at least 3 years.

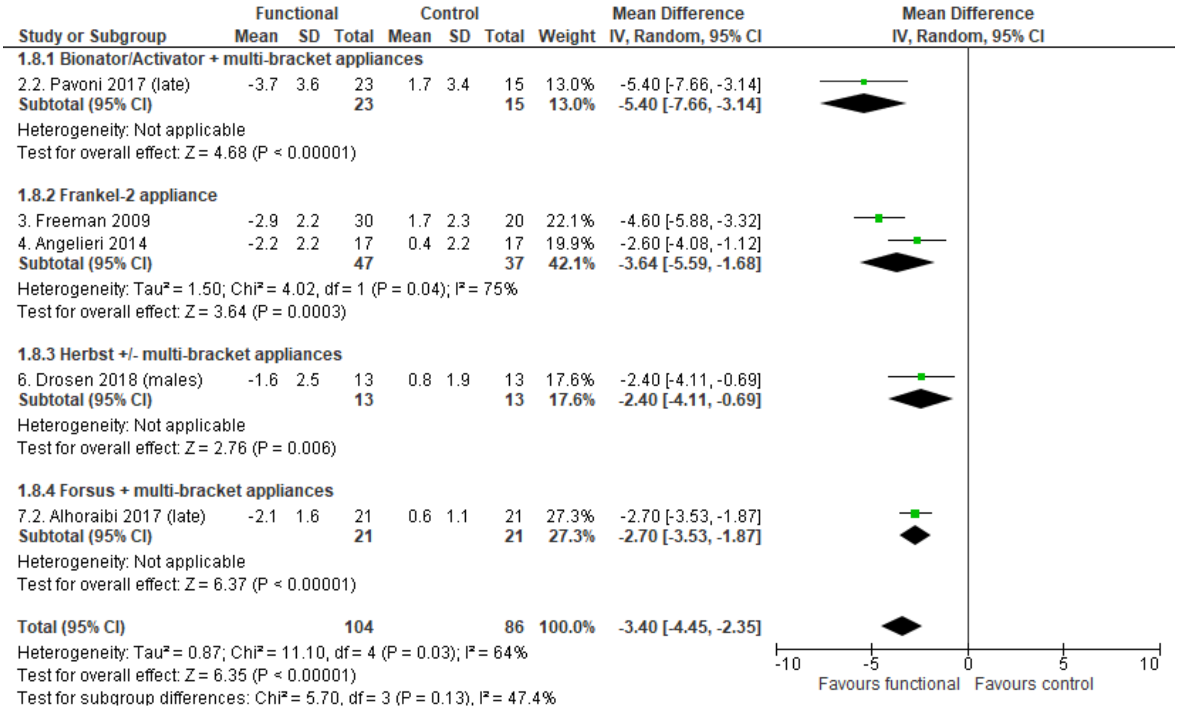

Figure 18. Meta-analysis; Outcome: Wits appraisal; Time point: above 18 years of age.

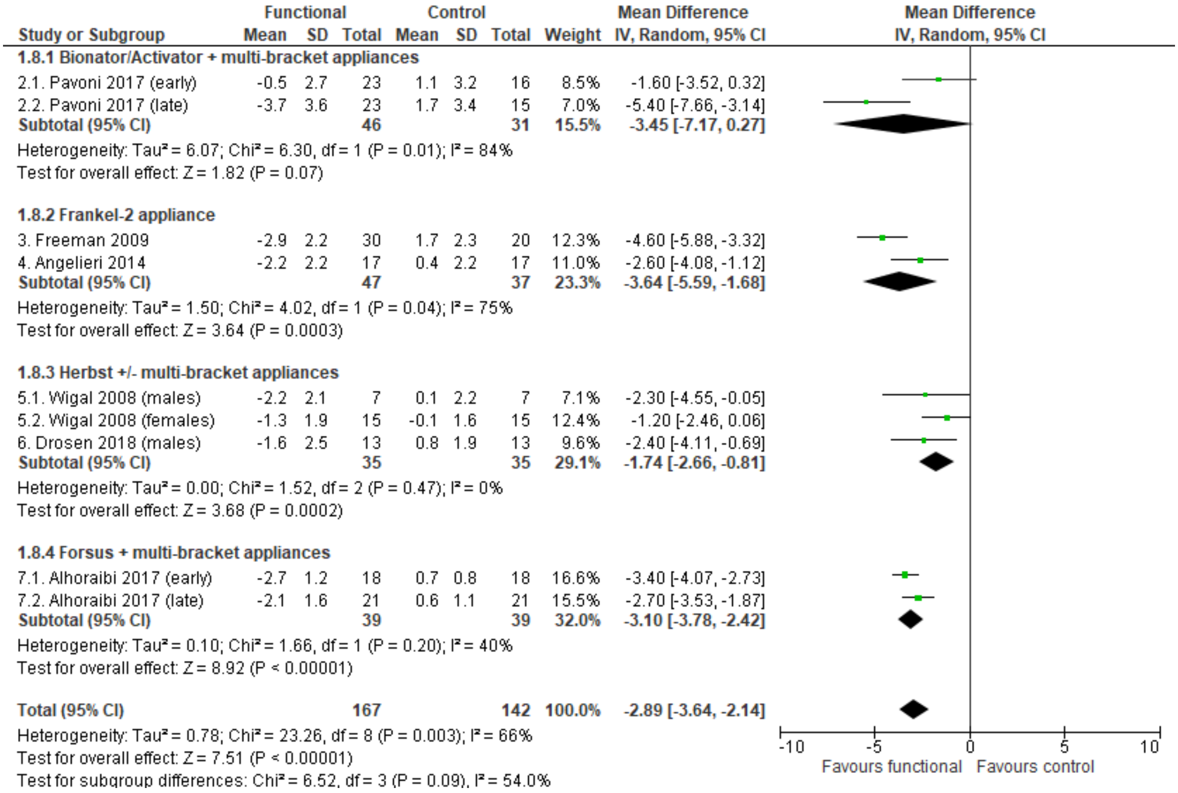

Figure 19. Meta-analysis; Outcome: Wits appraisal; Time point: After a post-retention period of at least 3 years.

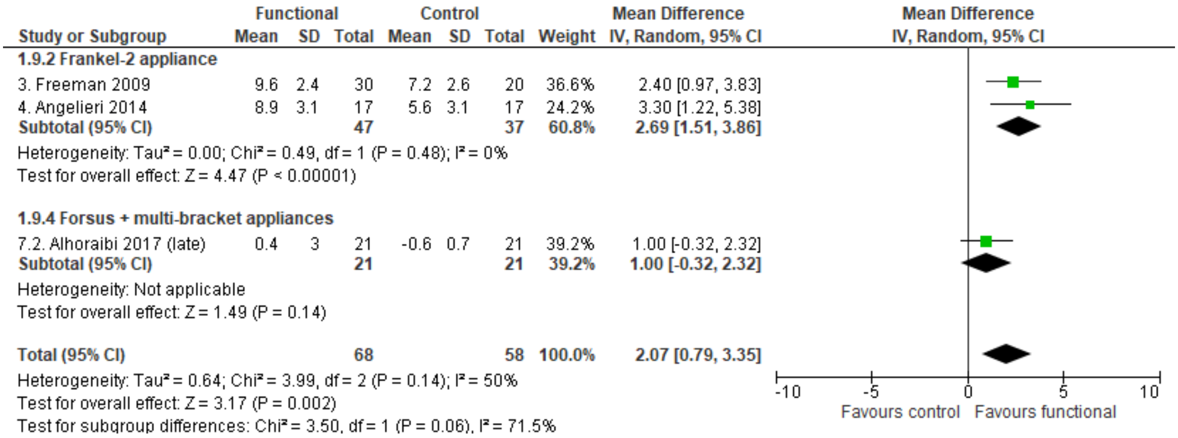

Figure 20. Meta-analysis; Outcome: Co-Gn/Co-A difference; Time point: above 18 years of age.

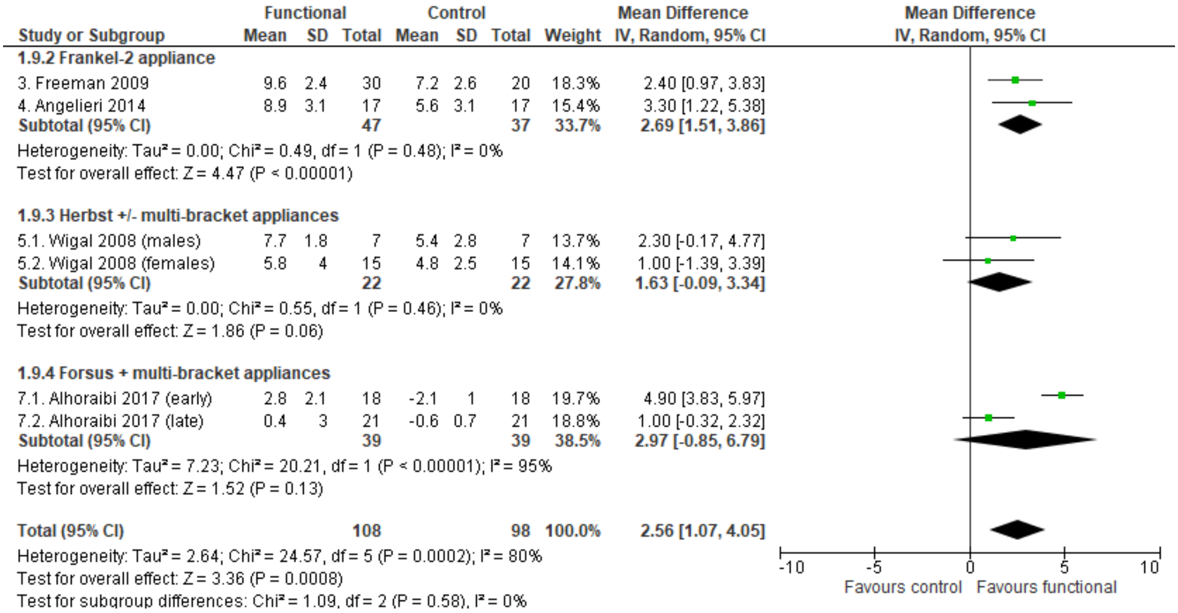

Figure 21. Meta-analysis; Outcome: Co-Gn/Co-A difference; Time point: After a post-retention period of at least 3 years.

Additional analysis

Study or Subgroup	Treated Mean	Treated SD	Treated Total	Control Mean	Control SD	Control Total	Weight	Mean Difference IV, Random, 95% CI
**2.1.1 Males**								
5.1. Wigal 2008 (males)	0.1	2.4	7	1.6	1.6	7	28.2%	-1.50 [-3.64, 0.64]
6. Drosen 2018 (males)	0.1	1.7	13	0.7	1.7	13	42.4%	-0.60 [-1.91, 0.71]
**Subtotal (95% CI)**			**20**			**20**	**70.6%**	**-0.85 [-1.96, 0.27]**
Heterogeneity: Tau² = 0.00; Chi² = 0.50, df = 1 (P = 0.48); I² = 0%								
Test for overall effect: Z = 1.49 (P = 0.14)								
**2.1.2 Females**								
5.2. Wigal 2008 (females)	-1.3	3.1	15	1.9	2.6	15	29.4%	-3.20 [-5.25, -1.15]
**Subtotal (95% CI)**			**15**			**15**	**29.4%**	**-3.20 [-5.25, -1.15]**
Heterogeneity: Not applicable								
Test for overall effect: Z = 3.06 (P = 0.002)								
**Total (95% CI)**			**35**			**35**	**100.0%**	**-1.62 [-3.17, -0.07]**
Heterogeneity: Tau² = 1.02; Chi² = 4.42, df = 2 (P = 0.11); I² = 55%								
Test for overall effect: Z = 2.05 (P = 0.04)								
Test for subgroup differences: Chi² = 3.92, df = 1 (P = 0.05), I² = 74.5%								

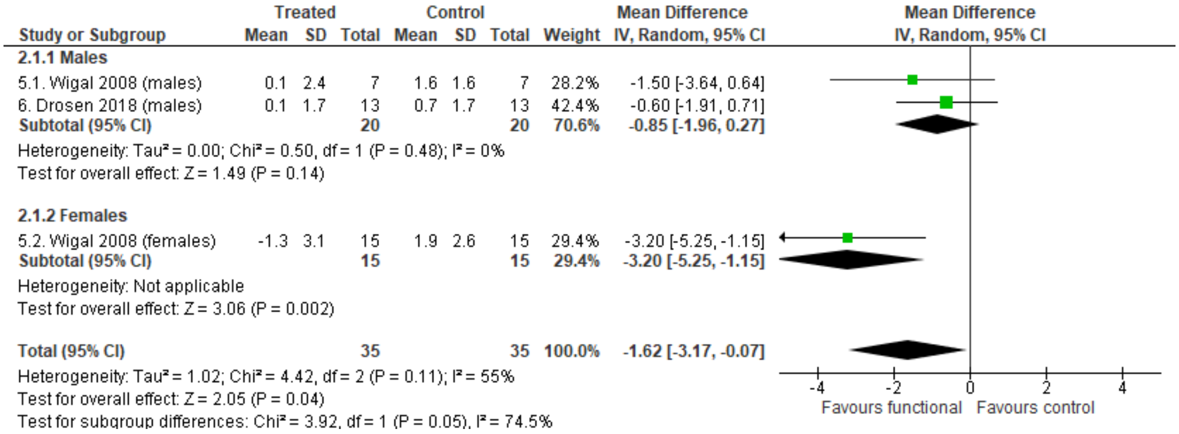

Figure 22. Subgroup analysis based on gender (males, females); Outcome: SNA angle.

Study or Subgroup	Treated Mean	Treated SD	Treated Total	Control Mean	Control SD	Control Total	Weight	Mean Difference IV, Random, 95% CI
**2.2.1 Males**								
5.1. Wigal 2008 (males)	15.4	4.5	7	14.1	3	7	43.0%	1.30 [-2.71, 5.31]
**Subtotal (95% CI)**			**7**			**7**	**43.0%**	**1.30 [-2.71, 5.31]**
Heterogeneity: Not applicable								
Test for overall effect: Z = 0.64 (P = 0.52)								
**2.2.2 Females**								
5.2. Wigal 2008 (females)	9.8	2.3	15	13.3	3	15	57.0%	-3.50 [-5.41, -1.59]
**Subtotal (95% CI)**			**15**			**15**	**57.0%**	**-3.50 [-5.41, -1.59]**
Heterogeneity: Not applicable								
Test for overall effect: Z = 3.59 (P = 0.0003)								
**Total (95% CI)**			**22**			**22**	**100.0%**	**-1.44 [-6.09, 3.22]**
Heterogeneity: Tau² = 8.95; Chi² = 4.49, df = 1 (P = 0.03); I² = 78%								
Test for overall effect: Z = 0.60 (P = 0.55)								
Test for subgroup differences: Chi² = 4.49, df = 1 (P = 0.03), I² = 77.7%								

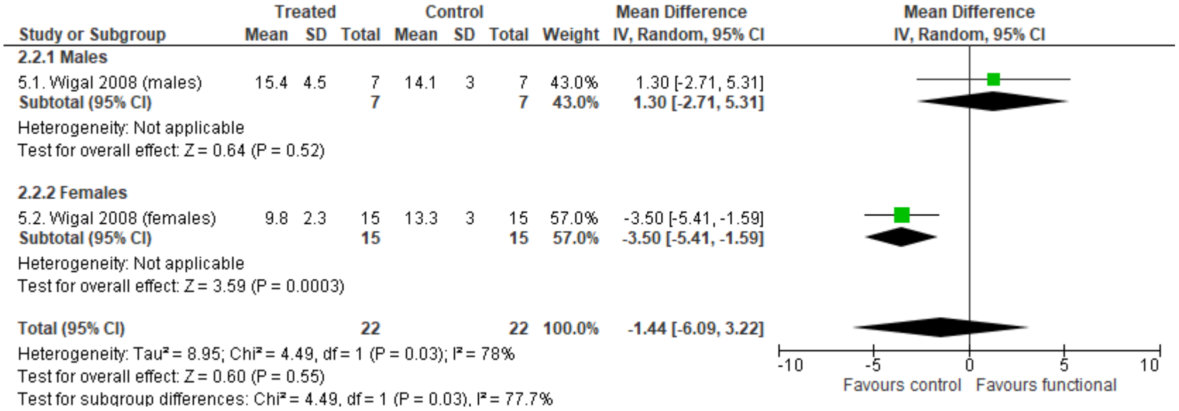

Figure 23. Subgroup analysis based on gender (males, females); Outcome: Co-Gn distance.

Study or Subgroup	Treated Mean	Treated SD	Treated Total	Control Mean	Control SD	Control Total	Weight	Mean Difference IV, Random, 95% CI
**2.3.1 Males**								
5.1. Wigal 2008 (males)	-2.1	1.6	7	0.2	1.2	7	28.2%	-2.30 [-3.78, -0.82]
6. Drosen 2018 (males)	-1.7	1.2	13	-1.3	1.2	13	37.5%	-0.40 [-1.32, 0.52]
**Subtotal (95% CI)**			**20**			**20**	**65.8%**	**-1.26 [-3.11, 0.60]**
Heterogeneity: Tau² = 1.41; Chi² = 4.55, df = 1 (P = 0.03); I² = 78%								
Test for overall effect: Z = 1.33 (P = 0.18)								
**2.3.2 Females**								
5.2. Wigal 2008 (females)	-2.3	1.7	15	-0.3	1.4	15	34.2%	-2.00 [-3.11, -0.89]
**Subtotal (95% CI)**			**15**			**15**	**34.2%**	**-2.00 [-3.11, -0.89]**
Heterogeneity: Not applicable								
Test for overall effect: Z = 3.52 (P = 0.0004)								
**Total (95% CI)**			**35**			**35**	**100.0%**	**-1.48 [-2.72, -0.25]**
Heterogeneity: Tau² = 0.84; Chi² = 6.92, df = 2 (P = 0.03); I² = 71%								
Test for overall effect: Z = 2.35 (P = 0.02)								
Test for subgroup differences: Chi² = 0.45, df = 1 (P = 0.50), I² = 0%								

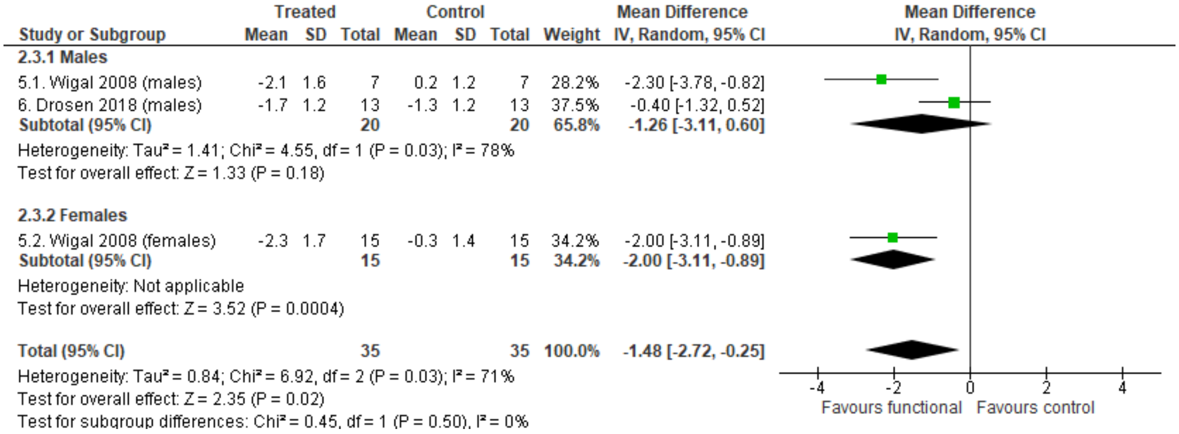

Figure 24. Subgroup analysis based on gender (males, females); Outcome: ANB angle.

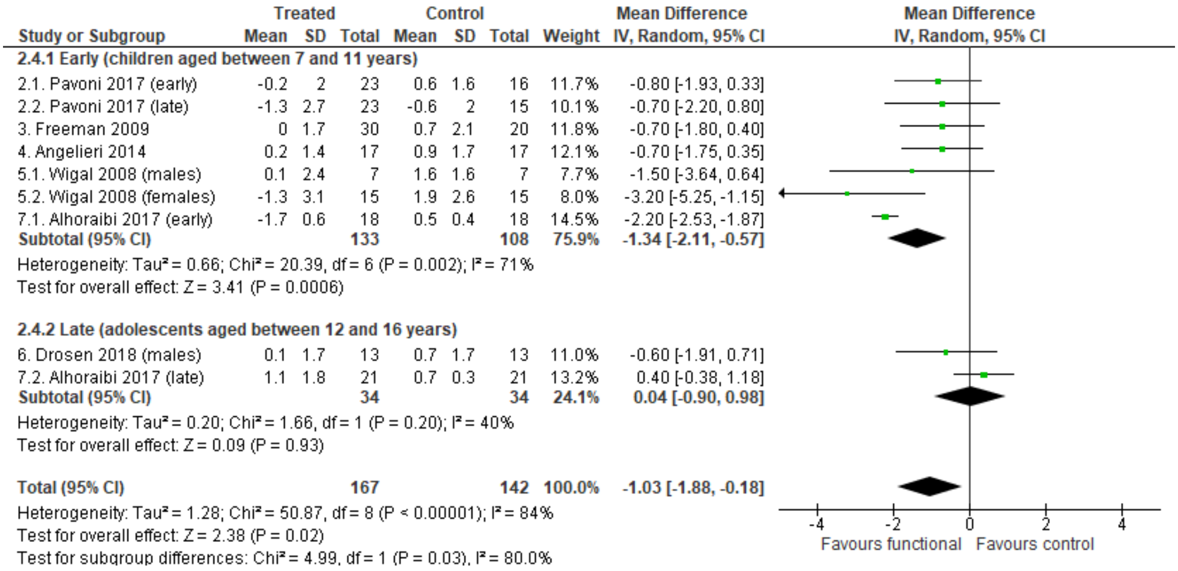

Figure 25. Subgroup analysis based on the beginning of the functional appliance therapy; Outcome: SNA angle. Early treatments, commencing in children aged between 7 and 11 years; late treatments, beginning in adolescents aged between 12 and 16 years.

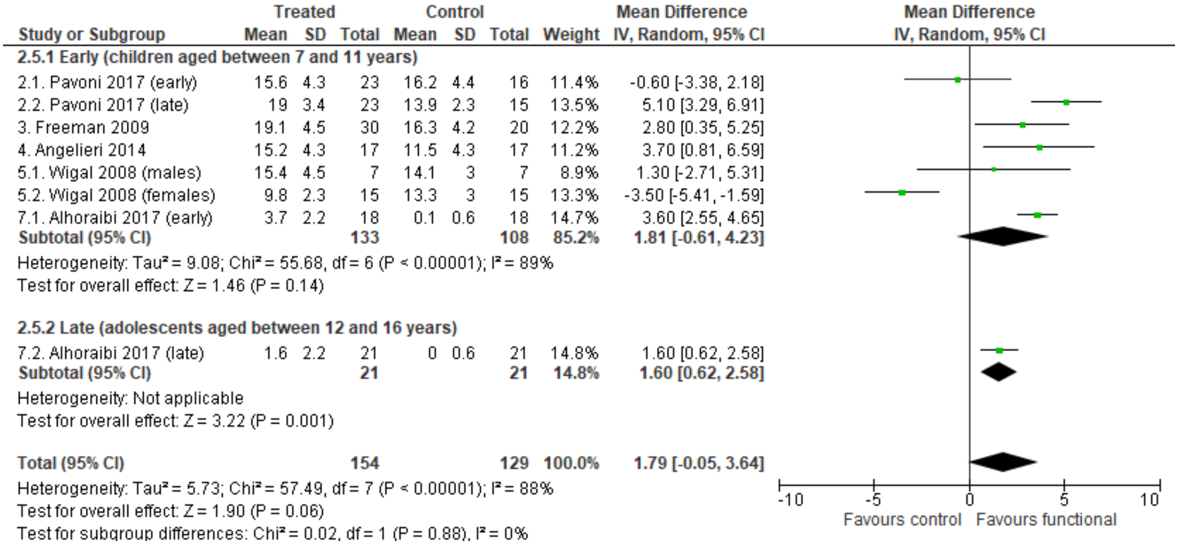

Figure 26. Subgroup analysis based on the beginning of the functional appliance therapy; Outcome: Co-Gn distance. Early treatments, commencing in children aged between 7 and 11 years; late treatments, beginning in adolescents aged between 12 and 16 years.

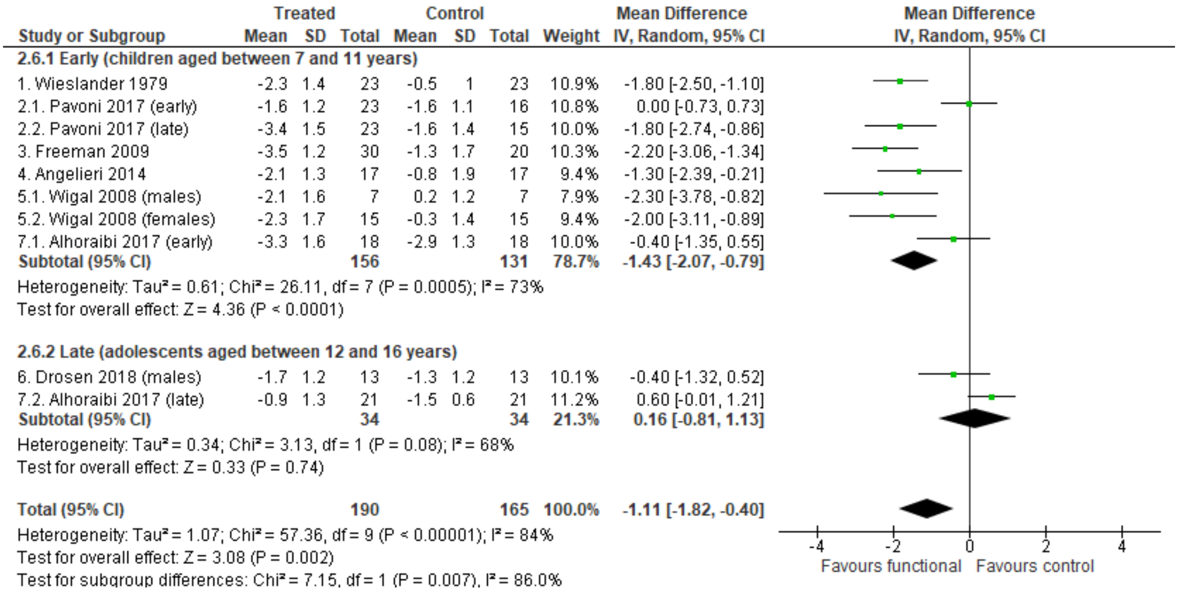

Figure 27. Subgroup analysis based on the beginning of the functional appliance therapy; Outcome: ANB angle. Early treatments, commencing in children aged between 7 and 11 years; late treatments, beginning in adolescents aged between 12 and 16 years.

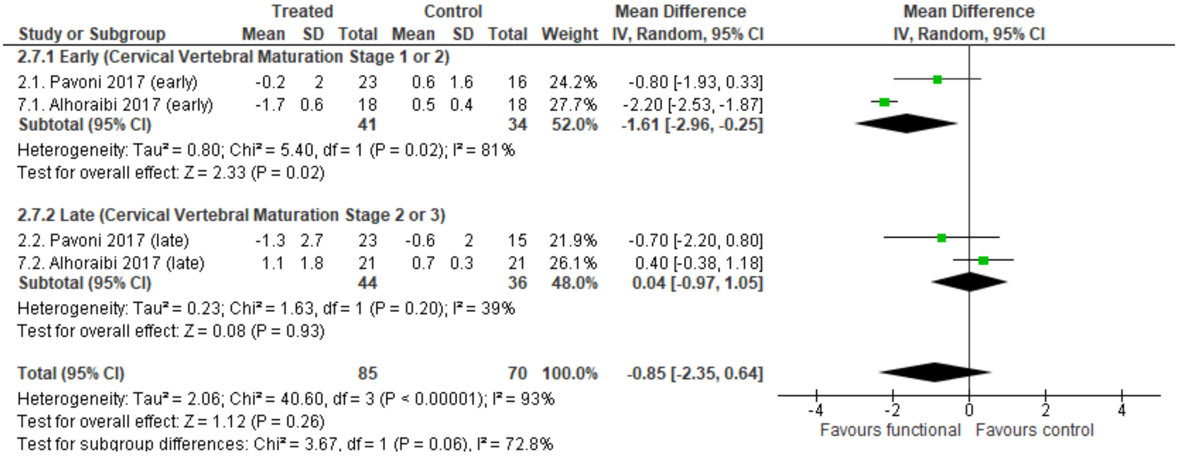

Figure 28. Subgroup analysis based on the start of the treatment; Outcome: SNA angle. Early treatments, with patients presenting with Cervical Vertebral Maturation Stage [CVMS] 1 or 2 at the first observation; late treatments, with subjects presenting with CVMS 2 or 3.

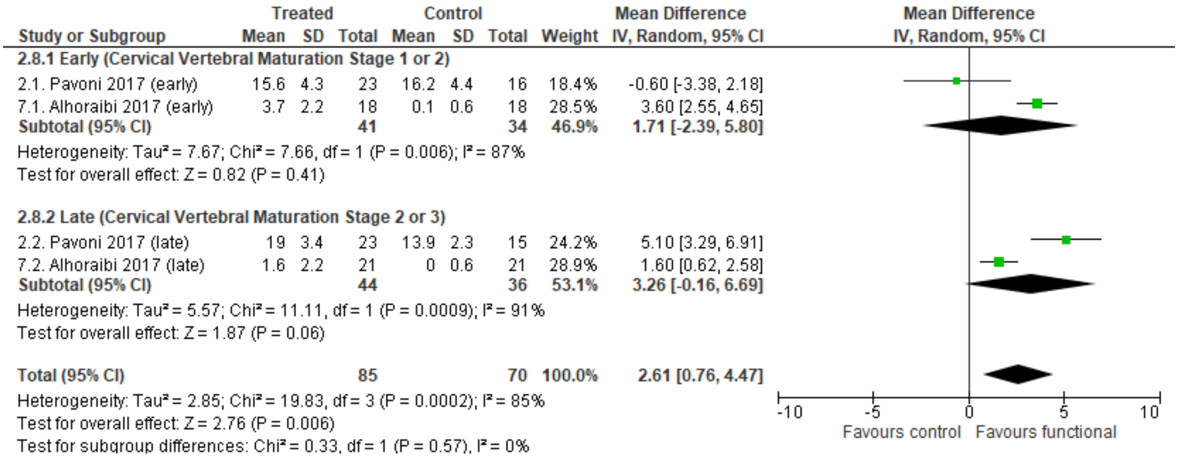

Figure 29. Subgroup analysis based on the start of the treatment; Outcome: Co-Gn distance. Early treatments, with patients presenting with Cervical Vertebral Maturation Stage [CVMS] 1 or 2 at the first observation; late treatments, with subjects presenting with CVMS 2 or 3.

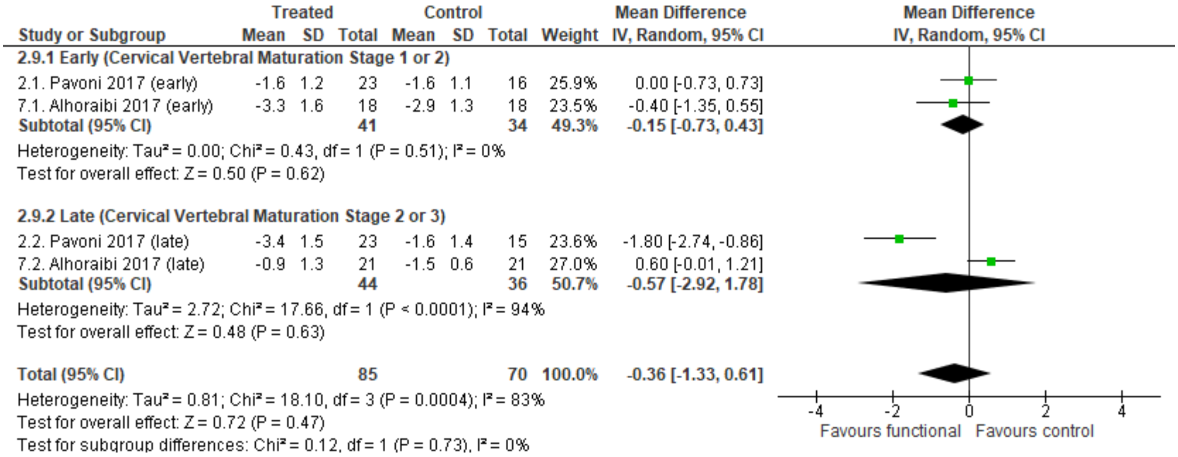

Figure 30. Subgroup analysis based on the start of the treatment; Outcome: ANB angle. Early treatments, with patients presenting with Cervical Vertebral Maturation Stage [CVMS] 1 or 2 at the first observation; late treatments, with subjects presenting with CVMS 2 or 3.

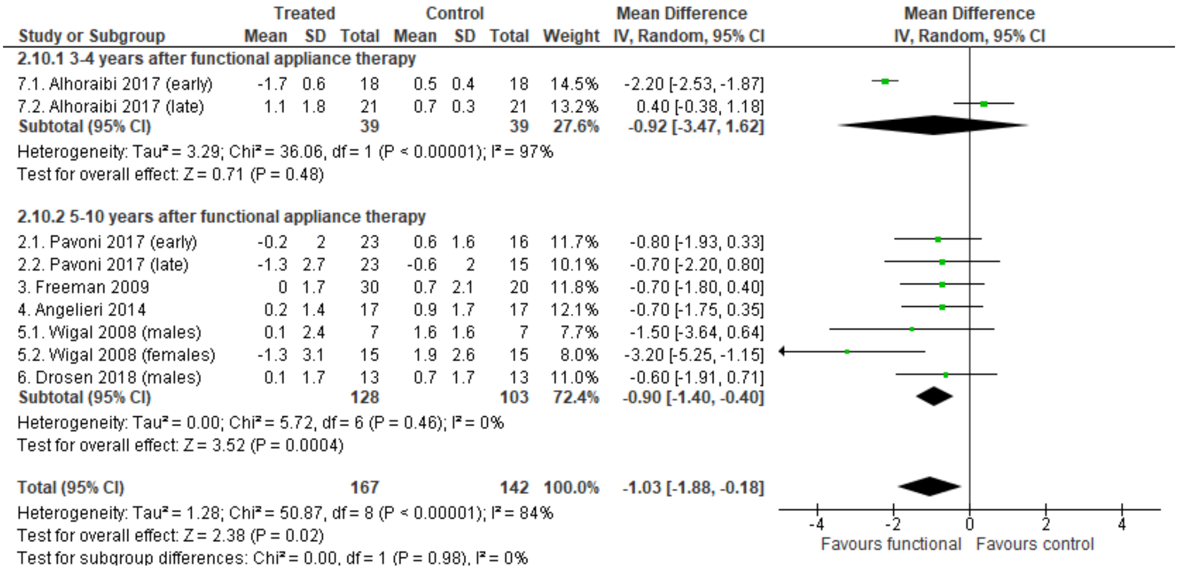

Figure 31. Subgroup analysis based on the post-retention period duration (3-4, 5-10 years after active treatment with functional appliances); Outcome: SNA angle.

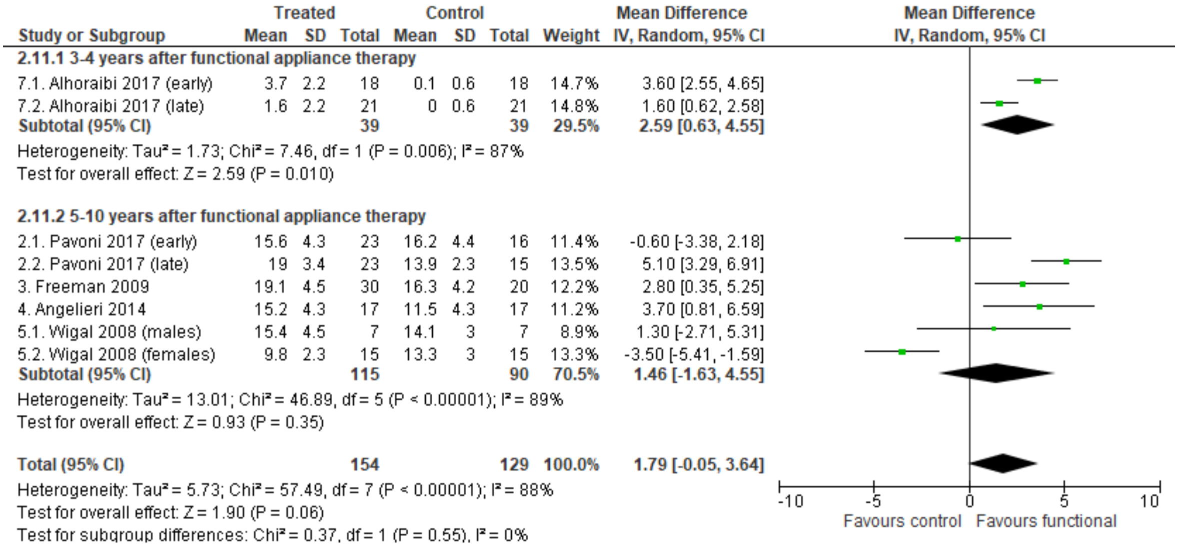

Figure 32. Subgroup analysis based on the post-retention period duration (3-4, 5-10 years after active treatment with functional appliances); Outcome: Co-Gn distance.

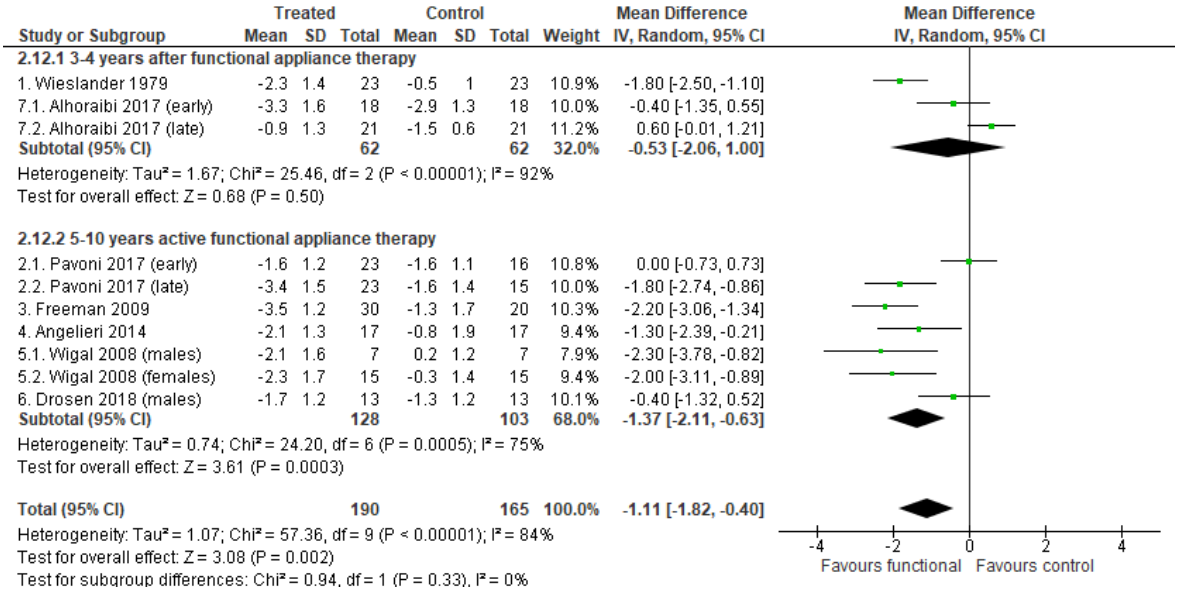

Figure 33. Subgroup analysis based on the post-retention period duration (3-4, 5-10 years after active treatment with functional appliances); Outcome: ANB angle.

Sensitivity analysis

Study or Subgroup	Treated Mean	Treated SD	Treated Total	Control Mean	Control SD	Control Total	Weight	Mean Difference IV, Random, 95% CI
**2.13.1 Low or moderate risk of bias**								
3. Freeman 2009	0	1.7	30	0.7	2.1	20	21.3%	-0.70 [-1.80, 0.40]
5.1. Wigal 2008 (males)	0.1	2.4	7	1.6	1.6	7	15.5%	-1.50 [-3.64, 0.64]
5.2. Wigal 2008 (females)	-1.3	3.1	15	1.9	2.6	15	16.0%	-3.20 [-5.25, -1.15]
7.1. Alhoraibi 2017 (early)	-1.7	0.6	18	0.5	0.4	18	24.3%	-2.20 [-2.53, -1.87]
7.2. Alhoraibi 2017 (late)	1.1	1.8	21	0.7	0.3	21	22.9%	0.40 [-0.38, 1.18]
**Subtotal (95% CI)**			**91**			**81**	**100.0%**	**-1.34 [-2.72, 0.05]**
Heterogeneity: Tau² = 2.03; Chi² = 41.62, df = 4 (P < 0.00001); I² = 90%								
Test for overall effect: Z = 1.89 (P = 0.06)								
**2.13.2 Critical or serious risk of bias**								
2.1. Pavoni 2017 (early)	-0.2	2	23	0.6	1.6	16	28.6%	-0.80 [-1.93, 0.33]
2.2. Pavoni 2017 (late)	-1.3	2.7	23	-0.6	2	15	16.4%	-0.70 [-2.20, 0.80]
4. Angelieri 2014	0.2	1.4	17	0.9	1.7	17	33.5%	-0.70 [-1.75, 0.35]
6. Drosen 2018 (males)	0.1	1.7	13	0.7	1.7	13	21.5%	-0.60 [-1.91, 0.71]
**Subtotal (95% CI)**			**76**			**61**	**100.0%**	**-0.71 [-1.31, -0.10]**
Heterogeneity: Tau² = 0.00; Chi² = 0.05, df = 3 (P = 1.00); I² = 0%								
Test for overall effect: Z = 2.29 (P = 0.02)								

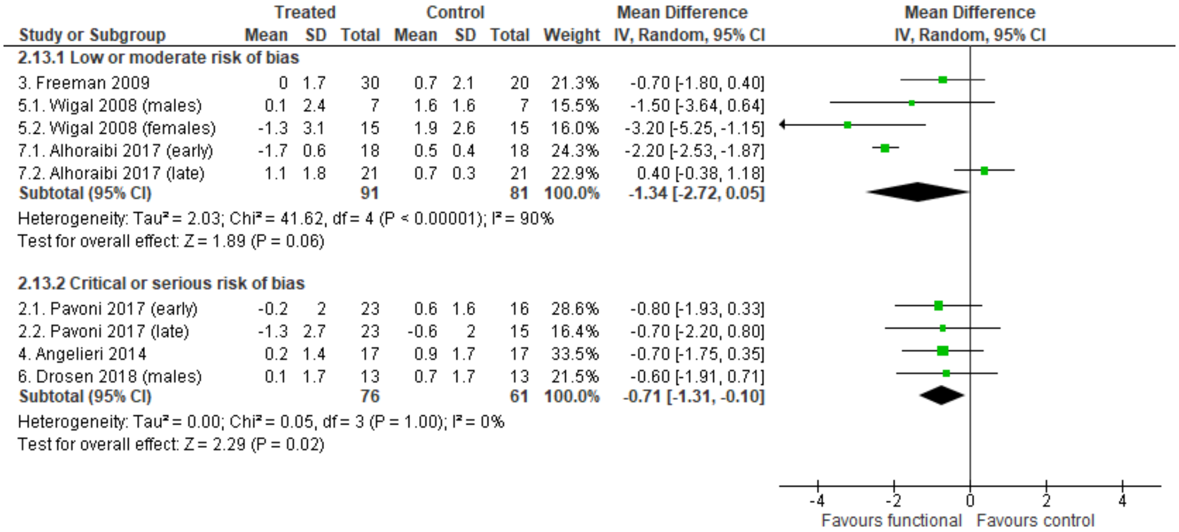

Figure 34. Sensitivity analysis based on the study quality assessment; Outcome: SNA angle.

Study or Subgroup	Treated Mean	Treated SD	Treated Total	Control Mean	Control SD	Control Total	Weight	Mean Difference IV, Random, 95% CI
**2.14.1 Low or moderate risk of bias**								
3. Freeman 2009	19.1	4.5	30	16.3	4.2	20	19.1%	2.80 [0.35, 5.25]
5.1. Wigal 2008 (males)	15.4	4.5	7	14.1	3	7	14.2%	1.30 [-2.71, 5.31]
5.2. Wigal 2008 (females)	9.8	2.3	15	13.3	3	15	20.7%	-3.50 [-5.41, -1.59]
7.1. Alhoraibi 2017 (early)	3.7	2.2	18	0.1	0.6	18	22.9%	3.60 [2.55, 4.65]
7.2. Alhoraibi 2017 (late)	1.6	2.2	21	0	0.6	21	23.1%	1.60 [0.62, 2.58]
**Subtotal (95% CI)**			**91**			**81**	**100.0%**	**1.19 [-1.17, 3.54]**
Heterogeneity: Tau² = 5.99; Chi² = 41.55, df = 4 (P < 0.00001); I² = 90%								
Test for overall effect: Z = 0.99 (P = 0.32)								
**2.14.2 Critical or serious risk of bias**								
2.1. Pavoni 2017 (early)	15.6	4.3	23	16.2	4.4	16	32.0%	-0.60 [-3.38, 2.18]
2.2. Pavoni 2017 (late)	19	3.4	23	13.9	2.3	15	36.5%	5.10 [3.29, 6.91]
4. Angelieri 2014	15.2	4.3	17	11.5	4.3	17	31.5%	3.70 [0.81, 6.59]
**Subtotal (95% CI)**			**63**			**48**	**100.0%**	**2.83 [-0.57, 6.23]**
Heterogeneity: Tau² = 7.39; Chi² = 11.36, df = 2 (P = 0.003); I² = 82%								
Test for overall effect: Z = 1.63 (P = 0.10)								

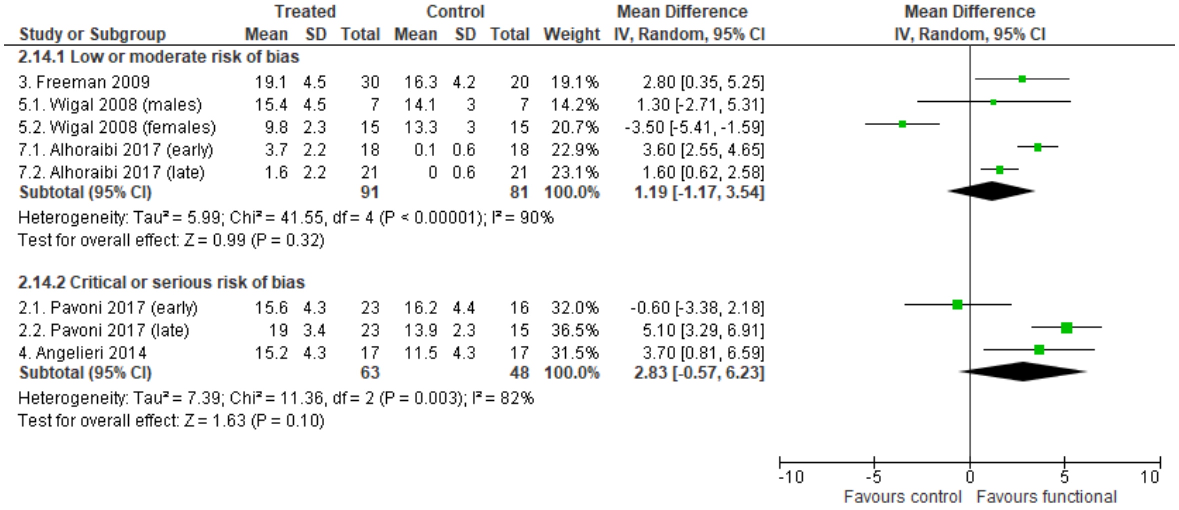

Figure 35. Sensitivity analysis based on the study quality assessment; Outcome: Co-Gn distance.

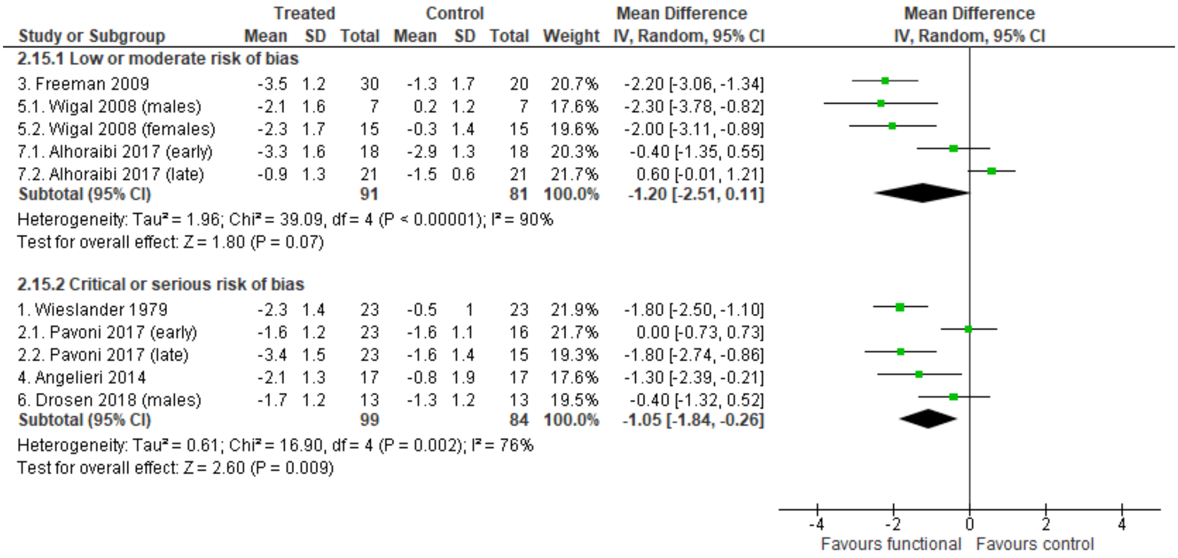

Figure 36. Sensitivity analysis based on the study quality assessment; Outcome: ANB angle.
